# Herbal medicine for the treatment of chronic rhinosinusitis: A systematic review and meta-analysis

**DOI:** 10.3389/fphar.2022.908941

**Published:** 2022-07-18

**Authors:** Boram Lee, Chan-Young Kwon, Man Young Park

**Affiliations:** ^1^ KM Science Research Division, Korea Institute of Oriental Medicine, Daejeon, South Korea; ^2^ Department of Oriental Neuropsychiatry, Dong-eui University College of Korean Medicine, Busan, South Korea; ^3^ Digital Health Research Division, Korea Institute of Oriental Medicine, Daejeon, South Korea

**Keywords:** herbal medicine, chronic rhinosinusitis, systematic review, meta-analysis, East Asian traditional medicine

## Abstract

**Objectives:** Chronic rhinosinusitis (CRS) is a disease with a high prevalence and a high socioeconomic burden. This study aimed to conduct a comprehensive systematic review to update the evidence on the use of herbal medicine (HM) for CRS treatment.

**Methods:** A total of 14 electronic databases for randomized controlled trials (RCTs) evaluating the effects of HM on the treatment of CRS were searched for articles published before July 2021. The primary outcome was CRS severity post-treatment, measured with the Visual Analogue Scale (VAS) and Total Effective Rate (TER). The risk of bias of the included studies and the quality of evidence of the main findings were assessed using the Cochrane Collaboration’s risk of bias tool and the Grading of Recommendations, Assessment, Development, and Evaluations tool.

**Results:** A total of 80 RCTs were included. Compared to placebo, HM significantly improved CRS severity as measured by TER and VAS. When HM was compared with conventional treatment (CT) as monotherapy or adjuvant therapy, CRS severity measured by TER and VAS, quality of life, Lund-Kennedy endoscopy score, Lund-Mackay computed tomography score, and nasal mucociliary function were significantly improved in the HM group. No serious adverse events associated with HM were reported. The risk of bias was generally unclear, and the quality of evidence ranged from moderate to low.

**Conclusion:** This review found some limited clinical evidence that HM or HM combined with CT may be more effective and safer than CT alone in treating CRS. However, the methodological quality of the included studies was generally low, and the quality of the evidence needs to be improved.

## 1 Introduction

Chronic rhinosinusitis (CRS) is diagnosed when two or more of the main symptoms (nasal obstruction, nasal drainage, facial pain/pressure, and hyposmia/anosmia) have been present for at least 12 weeks, with objective evidence from physical examination or radiography (Fokkens et al., 2020). According to reports, the prevalence of CRS in the general population is about 5–12% (Fokkens et al., 2020). CRS is associated with severe morbidity and poor health-related quality of life, and diagnosis places an enormous burden on healthcare services (Mahboubi, 2018; Son et al., 2018; Dietz de Loos et al., 2019). In the United States, the total direct costs associated with CRS range from $10 to $13 billion per year, and the total indirect costs due to reduced labor productivity are estimated to exceed $20 billion per year (Rudmik, 2017). Moreover, it is known that the clinical symptoms of CRS severely impair a person’s quality of life, and CRS is associated with some chronic conditions, such as depression, olfactory dysfunction, fatigue, sleep disturbance, and sexual dysfunction (Rudmik and Smith, 2011).

To date, the first-line conventional treatment (CT) for CRS is topical intranasal corticosteroids and saline irrigation. When CT does not lead to clinical response, oral corticosteroids, antibiotics, and surgical treatment are considered (Walker et al., 2019; Fokkens et al., 2020). However, studies have shown that the risks of these treatments outweigh the benefits, and there are problems with antibiotic resistance and a wide range of adverse reactions ranging from mild (rash, nausea, diarrhea) to life-threatening (anaphylaxis) (Leung et al., 2014; Lux et al., 2020). Refractory CRS, especially CRS accompanied by nasal polyps, is an indication for surgical treatment. Still, compared with intranasal or systemic steroids, there was no significant difference in self-reported symptoms and quality of life (Rimmer et al., 2014). Therefore, due to the limitations of CT and the chronic nature of the disease, patients seek complementary and integrative methods of treatment, and herbal medicine (HM) is one of such options (Rotenberg and Bertens, 2010; Mahboubi, 2018).

HM, one of the representative treatments of East Asian Traditional Medicine (EATM), is widely used to treat various diseases, including CRS, especially in East Asian countries. Recently, CRS has been considered a disease with a complex and multifactorial etiology rather than simple inflammation. Moreover, a holistic approach, which is the core concept of EATM, has been emphasized (Taw et al., 2013). In particular, EATM can contribute to treatment in a multifactorial view of CRS management via the use of a combination of herbs resistant to foreign pathogens and with immunomodulatory effects (Taw et al., 2013). In this regard, a systematic review of HM for the treatment of rhinosinusitis was published in 2006, which concluded that the evidence supporting the benefit of HM for the treatment of rhinosinusitis was limited (Guo et al., 2006). However, this review was focused on both acute and chronic patients and did not include local databases such as those of Korea, China, and Japan, so the effect of HM could not be evaluated comprehensively. In addition, although another systematic review was conducted in 2018 that examined the effectiveness of HM in CRS, it also had the limitation that the database search did not include local databases of East Asian countries where EATM is commonly used (Anushiravani et al., 2018). Given that it is recommended to include databases of traditional medicine when searching for studies of EATM modalities, such as HM or acupuncture, a systematic review on this topic needs to be further strengthened (Wang et al., 2019). Furthermore, many randomized controlled trials (RCTs) have since been published on the effects of various HM treatments (Son et al., 2018). Previous reviews have insufficiently addressed clinical heterogeneity in patients with CRS. For example, there are histopathological features of higher cellularity and more prominent lymphocytic infiltration in pediatric patients with CRS when compared with adults. By contrast, there is stronger eosinophilic infiltration and more prominent glandular hyperplasia in adults with CRS (Mahdavinia and Grammer, 2013; Snidvongs et al., 2020). Additionally, while adenoids play an important role in pediatric patients with CRS, they do not in adult patients (Mahdavinia and Grammer, 2013; Snidvongs et al., 2020). This suggests an age-dependent treatment response in patients with CRS. No previous reviews have clearly explained the influence of age. Moreover, pattern identification or *Zheng*, a characteristic of EATM, may play an important role in the treatment of CRS (Yang et al., 2012).

Therefore, we sought to update the evidence on the use of HM for the treatment of CRS by conducting a comprehensive systematic review and evaluation of the quality of evidence. We also focused on subgroup analysis according to the age of patients and the presence of patten identification.

## 2 Methods

We conducted this systematic review as per the Preferred Reporting Items for Systematic Reviews and Meta-Analyses (PRISMA) guidelines (Page et al., 2021). The protocol of this review was registered in PROSPERO (CRD42022301382).

### 2.1 Eligible criteria

#### 2.1.1 Study design

Only parallel-group RCTs were included in this review, with no restrictions on the language of publication. However, studies that mentioned only that randomization was performed but did not specify the method of randomization were excluded. Crossover studies were excluded to lower the risk of potential bias.

#### 2.1.2 Study participants

The included studies enrolled patients with CRS, regardless of age, sex, or race, in whom concomitant symptoms persisted for at least 12 weeks.

#### 2.1.3 Treatment and control interventions

With regard to treatment interventions, studies using oral HM based on the EATM theory were included. Studies that did not specify the individual constituent herbs of HM were excluded. As for control interventions, placebo HM, no medical treatment, and CTs, such as antibiotics and antihistamines, were allowed. We excluded studies using EATM therapies such as HM, acupuncture, and moxibustion as a control intervention. Studies involving HM combined with CT were also included if the use of CT was the same in both the treatment and control groups.

#### 2.1.4 Outcome measures

The primary outcome was the post-treatment CRS severity, measured using the Visual Analogue Scale (VAS) and Total Effective Rate (TER). TER is determined according to the indicators of improvement in quantified outcomes and symptoms classified as “cured” (*N1*), “markedly improved” (*N2*), “improved” (*N3*), or “non-responder” after treatment. It is calculated using the following formula: TER = (*N1 + N2 + N3*)/total sample size. Secondary outcomes included: 1) post-treatment health-related quality of life, measured using the Sino-Nasal Outcome Test (SNOT)-22 (Kennedy et al., 2013) and SNOT-20 (Piccirillo et al., 2002), 2) post-treatment endoscopy score, measured using the Lund-Kennedy scale (Lund and Kennedy, 1997), 3) post-treatment computed tomography scan score, measured using the Lund-Mackay scale (Lund and Mackay, 1993), 4) post-treatment nasal mucociliary function, measured using mucociliary transport rate (MTR) and mucociliary clearance rate (MCC), 5) frequency of CRS recurrence rate, and 6) incidence of adverse events (AEs) during the study period. For SNOT-22, SNOT-20, Lund-Kennedy scale, and Lund-Mackay scale, lower scores indicate better the quality of life. For MTR and MCC, higher scores indicate better nasal mucociliary function. Symptom and polyp recurrence rates were comprehensively evaluated to assess the CRS recurrence rate.

### 2.2 Information sources and search strategy

A comprehensive search of studies published between inception and July 6, 2021 was conducted in the following electronic databases: Medline (*via* PubMed), EMBASE (*via* Elsevier), Cochrane Central Register of Controlled Trials, Allied and Complementary Medicine Database (*via* EBSCO), Cumulative Index to Nursing and Allied Health Literature (*via* EBSCO), Korean databases (Oriental Medicine Advanced Searching Integrated System, Korean Studies Information Service System, Korean Medical Database, Research Information Sharing Service, and ScienceON), Chinese databases (China National Knowledge Infrastructure, Wanfang data, and Chongqing VIP), and a Japanese database (CiNii). We additionally reviewed the reference lists of included studies and trial registries, such as Clinicaltrials.gov, to include as many eligible studies as possible. Studies published in journals and gray literature, such as theses, dissertations, and conference proceedings, were considered. We set the final search strategy by consulting systematic review experts. The detailed search strategies for each database and the corresponding search results are presented in Supplementary Material S1.

### 2.3 Study selection and data extraction

Studies found in the databases and identified from other sources were imported into EndNote 20 (Clarivate Analytics, Philadelphia, United States). After the duplicates were removed, the titles and abstracts of the studies were reviewed for the first inclusion. Then, the full texts of eligible studies were retrieved and reviewed for final inclusion.

We extracted the following data from the included studies in a pre-tested pilot Excel form: basic study characteristics (the first author’s name and country, publication year, study setting, and funding sources), sample size, participant characteristics, treatment and control intervention, outcome measures, results, and information used to assess the risk of bias. Study selection and data extraction were independently performed by two researchers (BL and CYK), and any discrepancies were resolved by discussion with the corresponding author (MYP). If possible, we contacted the authors of the included studies by e-mail to inquire if the data were missing or insufficient.

### 2.4 Risk of bias assessment

The risk of bias for the included studies was assessed using the Cochrane Collaboration’s risk of bias tool (Higgins et al., 2011). The following domains were evaluated as “low risk,” “unclear risk,” or “high risk” of bias: random sequence generation, allocation concealment, blinding of participants and personnel, blinding of outcome assessors, completeness of outcome data, selective reporting, and other biases. In particular, we assessed the “other biases” domain based on the statistical homogeneity of the baseline clinical characteristics between the treatment and control groups. Two researchers (BL and CYK) independently assessed the risk of bias, and the consensus was reached through discussions with the corresponding author (MYP).

### 2.5 Data analysis and synthesis

For all the included studies, we conducted a descriptive analysis of details regarding participants, interventions, and outcomes. If two or more studies had the same treatment and control groups for our primary and secondary outcome measures, a meta-analysis was performed using the Review Manager (RevMan) software (version 5.4; Cochrane, London, United Kingdom). Continuous and binary results were presented in the form of mean difference (MD) and risk ratio (RR), along with 95% confidence intervals (CIs). Heterogeneity among the studies was assessed using the χ^2^-test and the *I* ^*2*^ statistic, and the *I* ^*2*^ value of ≥50% and ≥75% indicated substantial and considerable heterogeneity, respectively. A fixed-effects model was used if the heterogeneity was not significant (*I* ^*2*^ value < 50%) or if the number of studies included in the meta-analysis was small (Borenstein et al., 2010). Otherwise, a random-effects model was used.

To interpret the cause of the heterogeneity, we conducted a subgroup analysis according to 1) the age of participants (children, adults, and both) and 2) whether the pattern identification of the participants was specified. Sensitivity analysis was planned to determine the robustness of the meta-analysis results by excluding 1) studies with a high risk of bias and 2) numerical outliers. Evidence of publication bias was assessed with the Egger’s test using STATA/MP software version 16.1 (StataCorp LLC, Texas, United States) if ten or more studies were included in each meta-analysis.

### 2.6 Quality of evidence assessment

The quality of evidence for each finding was evaluated using the Grading of Recommendations, Assessment, Development and Evaluations (GRADE) tool. The following domains were assessed using GRADEpro (https://gradepro.org/): the risk of bias, inconsistency, indirectness, imprecision, and publication bias (Guyatt et al., 2008). Overall evaluation results were presented as “very low,” “low,” “moderate,” or “high.” One researcher (BL) performed the assessment, and another researcher (CYK) reviewed the results. Any discrepancies were resolved by discussion between them.

## 3 Results

### 3.1 Study selection

**FIGURE 1:**
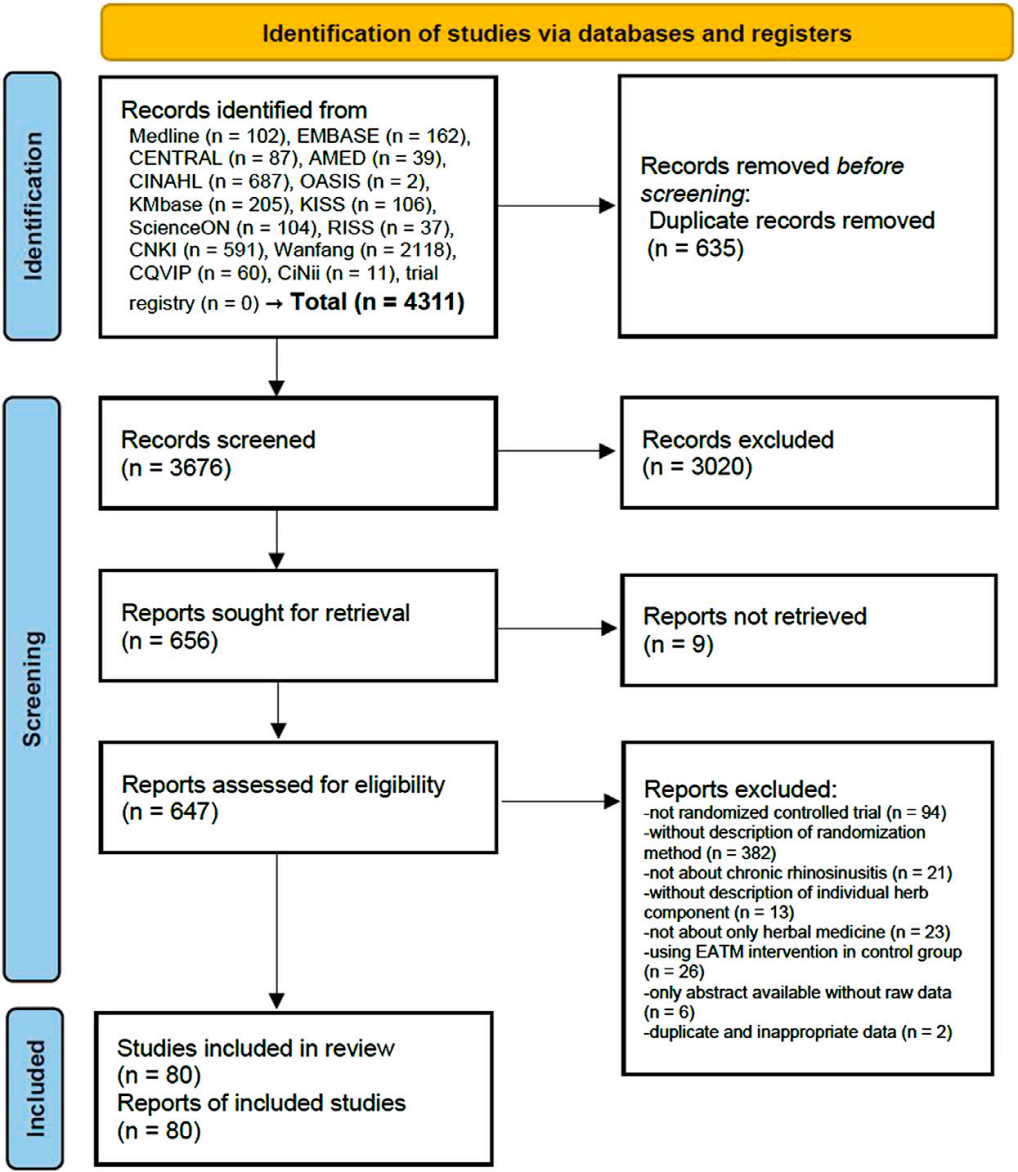
PRISMA 2020 flow diagram. No studies were identified from the search of reference lists.

A total of 4,311 studies were retrieved from the databases, and no additional studies were identified by searching the reference lists. After removing the duplicates, the titles and abstracts of 3,676 studies were screened. Afterward, a full text was searched for 656 suitable studies, and a total of 647 full-text studies were screened for eligibility, while the full text was not retrieved for the remaining 9. A total of 567 studies were excluded for the following reasons: not RCTs (*n* = 94), without a description of the randomization method (*n* = 382), not about CRS (*n* = 21), without a description of an individual herb component (*n* = 13), not about HM only (*n* = 23), using EATM intervention in the control group (*n* = 26), only abstract available without raw data (n = 6), as well as duplicate and inappropriate data (*n* = 2) (Supplementary Material S2). Finally, 80 studies (Liang et al., 2004; Zhang, 2004; Chen, 2005; Zou, 2007; Zhou, 2008; Lin and Qiu, 2010; Xin et al., 2010; Lu, 2011; Ding, 2012; Jiang et al., 2012; Liu et al., 2012; Zhou et al., 2012; Dai et al., 2013; Lin et al., 2013; Shen et al., 2013; Xu, 2013; Yang and Zhu, 2013; Zhou et al., 2013; Zhu et al., 2014; Huang, 2015; Li et al., 2015; Xing, 2015; Zhang, 2015; Cao, 2016; Chen et al., 2016; Du, 2016; Ma, 2016; Wang and Ma, 2016; Yang, 2016; Zhang et al., 2016; Zhang and Gou, 2016; Chen, 2017; Lin and Cao, 2017; Liu, 2017; Sun and Chen, 2017; Wang et al., 2017; Wei, 2017; Xiang et al., 2017; Chai et al., 2018; Hou, 2018; Li, 2018; Li et al., 2018; Liu et al., 2018; Yang, 2018; Zhang, 2018; Chen, 2019; Chen et al., 2019; Hu, 2019; Liu, 2019; Qian et al., 2019; Shao et al., 2019; Xia et al., 2019; Xu, 2019; Yao et al., 2019; Yun, 2019; Zhang, 2019; Zhang et al., 2019; Wang J. Q., 2020; Wang X., 2020; Fan, 2020; Fu and Zhang, 2020; Gou et al., 2020; He et al., 2020; Huang, 2020; Liao, 2020; Lin et al., 2020; Liu and Xu, 2020; Ma et al., 2020; Peng and Zhou, 2020; Shen and Li, 2020; Tang, 2020; Tao and He, 2020; Yang, 2020; Zhang, 2020; Jiang, 2021; Jiang et al., 2021; Li, 2021; Song et al., 2021; Xie et al., 2021; Zhang et al., 2021) were included in the review (Figure 1).

### 3.2 Study characteristics

Seventy-nine studies were conducted in China, and the remaining 1 study (Jiang et al., 2012) was conducted in Taiwan. Twelve studies (Liu et al., 2012; Lin et al., 2013; Zhou et al., 2013; Zhu et al., 2014; Liu et al., 2018; Chen et al., 2019; Zhang et al., 2019; He et al., 2020; Shen and Li, 2020; Tao and He, 2020; Jiang et al., 2021; Zhang et al., 2021) described their funding source, and all studies were supported by the federal or provincial government. Fourteen studies enrolled children, 52 studies enrolled adults, and the remaining 14 enrolled both children and adults. In 22 studies, pattern identification was set as the inclusion criterion, and dual deficiency of the lung-spleen (4 studies) and gallbladder heat depression (4 studies) were the most common, followed by wind-heat invading the lung meridian (3 studies). Two studies compared HM and placebo HM, 14 studies compared HM and CT, 63 studies compared HM plus CT and CT alone, and 1 study compared HM and no treatment. As outcome measures, TER was reported the most frequently (71 studies), followed by VAS (25 studies), Lund-Kennedy endoscopy score (20 studies), Lund-Mackay computed tomography score (15 studies), and MTR and SNOT-20 (12 studies each) (Supplementary Material S3). Prior to initiation, 26 studies (Jiang et al., 2012; Xu, 2013; Chen et al., 2016; Lin and Cao, 2017; Liu, 2017; Sun and Chen, 2017; Wang et al., 2017; Xiang et al., 2017; Chai et al., 2018; Liu et al., 2018; Chen, 2019; Shao et al., 2019; Xia et al., 2019; Gou et al., 2020; He et al., 2020; Liao, 2020; Lin et al., 2020; Liu and Xu, 2020; Peng and Zhou, 2020; Shen and Li, 2020; Tao and He, 2020; Jiang, 2021; Jiang et al., 2021; Li, 2021; Xie et al., 2021; Zhang et al., 2021) were approved by the institutional review board, and 54 studies (Liang et al., 2004; Jiang et al., 2012; Liu et al., 2012; Dai et al., 2013; Xu, 2013; Zhou et al., 2013; Zhu et al., 2014; Huang, 2015; Zhang, 2015; Chen et al., 2016; Du, 2016; Yang, 2016; Zhang et al., 2016; Zhang and Gou, 2016; Lin and Cao, 2017; Liu, 2017; Sun and Chen, 2017; Wang et al., 2017; Wei, 2017; Xiang et al., 2017; Chai et al., 2018; Li et al., 2018; Liu et al., 2018; Zhang, 2018; Chen, 2019; Chen et al., 2019; Liu, 2019; Qian et al., 2019; Shao et al., 2019; Xia et al., 2019; Xu, 2019; Yao et al., 2019; Yun, 2019; Zhang, 2019; Zhang et al., 2019; Wang X., 2020; Fu and Zhang, 2020; Gou et al., 2020; He et al., 2020; Liao, 2020; Lin et al., 2020; Liu and Xu, 2020; Ma et al., 2020; Peng and Zhou, 2020; Shen and Li, 2020; Tang, 2020; Tao and He, 2020; Zhang, 2020; Jiang, 2021; Jiang et al., 2021; Li, 2021; Song et al., 2021; Xie et al., 2021; Zhang et al., 2021) obtained informed consent from the participants.

The included studies used a wide range of HM modalities. Among them, Biyuan tongqiao granule was the most often used (13 studies), followed by Biyuan decoction and Biyuanshu oral liquid (4 studies each). As for the dosage form, the decoction was the most common (47 studies), followed by granules (19 studies) and capsules (6 studies). When examining the individual constituent herbs of HM, *Magnolia denudata* Desr. [Magnoliaceae; Magnoliae Flos] was used the most often (63 studies), followed by *Xanthium strumarium* L. [Asteraceae; Xanthii Fructus] (61 studies), *Angelica dahurica* Benth. et Hooker f. [Apiaceae; Angelicae Dahuricae Radix] (57 studies), *Glycyrrhiza uralensis* Fisch. [Leguminosae; Glycyrrhizae Radix] (51 studies), *Scutellaria baicalensis* Georgi [Labiatae; Scutellariae Radix] (43 studies), *Mentha arvensis* var. *piperascens* Makinv. [Lamiaceae; Menthae Herba] (33 studies), *Ligusticum chuanxiong* Hort [Apiaceae; Ligustici Rhizoma] (31 studies), *Poria cocos* (Schw.) Wolf [Polyporaceae; Poria (Hoelen)] (29 studies), *Astragalus membranaceus* Bunge [Leguminosae; Astragali Radix] (27 studies), *Ephedra sinica* Stapf. [Ephedraceae; Ephedrae Herba] (24 studies), *Platycodon grandiflorum* (Jacq.) A. DC. [Campanulaceae; Platycodi Radix] (23 studies), *Atractylodes macrocepha-la* Koidz [Asteraceae; Atractylodis Rhizoma Alba] (22 studies), and *Saururus chinensis* Baill. [Saururaceae; Houttuyniae Herba] (21 studies). The most common duration of HM administration was 4 weeks (22 studies), followed by 2 weeks (7 studies), 8 weeks (6 studies), 3 months (6 studies), and 12 weeks (6 studies). Twenty-four studies conducted follow-up assessments after the HM treatment period: the most common was a 6-months follow-up (8 studies), followed by a 3-months follow-up (4 studies) (Supplementary Material S4).

### 3.3 Risk of bias assessment

All studies reported on appropriate methods for random sequence generation, such as random number tables. There were only two studies (Liang et al., 2004; Jiang et al., 2012) that reported that group allocation was concealed using opaque sealed envelopes, and the associated risk of bias was evaluated as low. In four studies (Jiang et al., 2012; Liu et al., 2012; Zhou et al., 2013; Lin et al., 2020), participants and personnel were blinded with placebo used as a control group, and in one study (Liang et al., 2004), blinding was not performed due to operational difficulties. All studies did not mention the blinding of outcome assessors, so the detection bias was rated as unclear. One study (Xin et al., 2010) reported having missing values but did not specify the reason and did not perform the intention-to-treat analysis, so the risk of attrition bias was rated as high. In addition, 5 studies (Xin et al., 2010; Shen et al., 2013; Du, 2016; Tang, 2020; Xie et al., 2021) did not report the results of the outcome measures presented under their methods and were rated as having a high risk of reporting bias. Two studies (Chen, 2005; Yang and Zhu, 2013) did not mention the statistical homogeneity of the treatment and control groups at baseline and were rated as having an unclear risk of other biases (Supplementary Material S5).

### 3.4 HM versus placebo

One study (Zhou et al., 2013) compared adult CRS participants of gallbladder heat stagnation pattern identification who received a decoction of Longdan Xiegan versus placebo for 2 weeks. As a result, TER was significantly higher in the HM group after treatment (*p* < 0.05), and SNOT-20 and VAS were significantly improved in the HM group (*p* < 0.05, both). Another study (Lin et al., 2020) compared adult patients with CRS without nasal polyps who received a Lianhuaqingwen granule versus placebo for 4 weeks. After treatment, VAS and SNOT-22 scores significantly improved in the HM group (*p* < 0.01, both), although meta-analysis was not possible because only median and range data were available. According to the reported AEs, one study (Lin et al., 2020) reported that stomachache and diarrhea occurred in 8 cases in the HM group and 3 cases in the placebo group. In another study (Zhou et al., 2013), diarrhea occurred in 1 case in the HM group, and there were no AEs in the placebo group (2 studies, RR 2.71, 95% CI 0.83 to 8.93, *I* ^*2*^ = 0%) (Table 1).

**TABLE 1:** Summary of findings.

Outcomes	Subgroup	No. participants (RCTs)	Anticipated absolute effects (95% CI)		Relative effect [95% CI]	*I* ^*2*^ value (%)	Quality of evidence (GRADE)	Comments
			Risk with control group	Risk with treatment group				
HM vs placebo HM								
Adverse events	Total (adults)	200 (6)	30 per 1,000	81 per 1,000 (25–268)	RR 2.71 [0.83, 8.93]	0	Moderate	Imprecision (-1)
Subgroup 1	PI	60 (1)	0 per 1,000	0 per 1,000 (0 to 0)	RR 3.00 [0.13, 70.83]	NA	Moderate	Imprecision (-1)
	no PI	140 (1)	43 per 1,000	114 per 1,000 (32–413)	RR 2.67 [0.74, 9.64]	NA	Moderate	Imprecision (-1)
**HM vs conventional treatment**								
TER	Total	1,282 (11)	771 per 1,000	925 per 1,000 (855–1,000)	RR 1.20 [1.11, 1.31]	63	Very low	Risk of bias (-1)
								Inconsistency (-1)
								Publication bias (-1)
Subgroup 1	children	180 (2)	742 per 1,000	927 per 1,000 (823–1,000)	RR 1.25 [1.11, 1.42]	0	Moderate	Risk of bias (-1)
	adults	627 (6)	763 per 1,000	915 per 1,000 (839–999)	RR 1.20 [1.10, 1.31]	29	Moderate	Risk of bias (-1)
	both	475 (3)	791 per 1,000	933 per 1,000 (720–1,000)	RR 1.18 [0.91, 1.53]	84	Very low	Risk of bias (-1)
								Inconsistency (-2)
Subgroup 2	PI	411 (4)	794 per 1,000	921 per 1,000 (850–1,000)	RR 1.16 [1.07, 1.26]	9	Moderate	Risk of bias (-1)
	no PI	871 (7)	760 per 1,000	927 per 1,000 (813–1,000)	RR 1.22 [1.07, 1.40]	75	Very low	Risk of bias (-1)
								Inconsistency (-2)
VAS	Total	482 (5)	—	MD 0.73 lower (0.97 to 0.5 lower)	—	81	Very low	Risk of bias (-1)
								Inconsistency (-2)
Subgroup 1	adults	365 (4)	—	MD 1.28 lower (2.85 lower to 0.29 higher)	—	86	Very low	Risk of bias (-1)
								Inconsistency (-2)
								Imprecision (-1)
	both	117 (1)	—	MD 0.78 lower (0.86 to 0.7 lower)	—	NA	Moderate	Risk of bias (-1)
Subgroup 2	PI	317 (3)	—	MD 2.6 lower (5.23 lower to 0.04 higher)	—	79	Moderate	Risk of bias (-1)
	no PI	165 (2)	—	MD 0.02 lower (1.64 lower to 1.6 higher)	—	91	Very low	Risk of bias (-1)
								Inconsistency (-2)
								Imprecision (-1)
Lund-Kennedy endoscopic score	Total (adults)	570 (6)	—	MD 0.7 lower (1.1 to 0.29 lower)	—	93	Moderate	Risk of bias (-1)
Subgroup 1	PI	317 (3)	—	MD 0.86 lower (0.99 to 0.74 lower)	—	0	Moderate	Risk of bias (-1)
	no PI	253 (3)	—	MD 0.62 lower (1.22 to 0.01 lower)	—	95	Moderate	Risk of bias (-1)
Lund-Mackay CT score	Total (adults)	558 (6)	—	MD 1.31 lower (2.41 to 0.22 lower)	—	94	Very low	Risk of bias (-1)
								Inconsistency (-2)
Subgroup 1	PI	310 (3)	—	MD 2.43 lower (3.92 to 0.93 lower)	—	68	Moderate	Risk of bias (-1)
	no PI	248 (3)	—	MD 0.38 lower (2.1 lower to 1.33 higher)	—	93	Very low	Risk of bias (-1)
								Inconsistency (-2)
**HM plus conventional treatment vs conventional treatment alone**								
TER	Total	6,490 (58)	760 per 1,000	936 per 1,000 (913–951)	RR 1.23 [1.20, 1.25]	27	Low	Risk of bias (-1)
								Publication bias (-1)
Subgroup 1	children	1,393 (12)	773 per 1,000	928 per 1,000 (881–966)	RR 1.20 [1.14, 1.25]	39	Low	Risk of bias (-1)
								Publication bias (-1)
	adults	3,698 (35)	733 per 1,000	932 per 1,000 (902–961)	RR 1.27 [1.23, 1.31]	0	Moderate	Risk of bias (-1)
	both	1,399 (11)	819 per 1,000	942 per 1,000 (909–983)	RR 1.15 [1.11, 1.20]	0	Moderate	Risk of bias (-1)
Subgroup 2	PI	1,377 (16)	750 per 1,000	915 per 1,000 (878–968)	RR 1.22 [1.17, 1.29]	19	Low	Risk of bias (-1)
								Publication bias (-1)
	no PI	5,113 (42)	763 per 1,000	939 per 1,000 (916–962)	RR 1.23 [1.20, 1.26]	31	Low	Risk of bias (-1)
								Publication bias (-1)
VAS	Total	993 (11)	—	MD 2.66 lower (3.25 to 2.07 lower)	—	98	Low	Risk of bias (-1)
								Publication bias (-1)
Subgroup 1	children	301 (3)	—	MD 7.91 lower (8.6 to 7.23 lower)	—	0	Moderate	Risk of bias (-1)
	adults	692 (8)	—	MD 1.17 lower (1.5 to 0.83 lower)	—	94	Moderate	Risk of bias (-1)
Subgroup 2	PI	240 (3)	—	MD 0.9 lower (1.03 to 0.77 lower)	—	0	Moderate	Risk of bias (-1)
	no PI	753 (8)	—	MD 3.48 lower (4.38 to 2.59 lower)	—	99	Moderate	Risk of bias (-1)
SNOT-20	Total (adults)	312 (4)	—	MD 2.76 lower (3.04 to 2.48 lower)	—	89	Moderate	Risk of bias (-1)
Subgroup 1	PI	252 (3)	—	MD 3.91 lower (4.45 to 3.37 lower)	—	44	Moderate	Risk of bias (-1)
	no PI	60 (1)	—	MD 2.33 lower (2.66 to 2 lower)	—	NA	Low	Risk of bias (-1)<
								Imprecision (-1)
Lund-Kennedy endoscopic score	Total	1,407 (14)	—	MD 2.11 lower (2.83 to 1.38 lower)	—	98	Low	Risk of bias (-1)
								Publication bias (-1)
Subgroup 1	children	377 (4)	—	MD 3.67 lower (4.44 to 2.91 lower)	—	88	Moderate	Risk of bias (-1)
	adults	836 (9)	—	MD 1.54 lower (2.27 to 0.81 lower)	—	98	Moderate	Risk of bias (-1)
	both	194 (1)	—	MD 1.12 lower (1.61 to 0.63 lower)	—	NA	Moderate	Risk of bias (-1)
Subgroup 2	PI	398 (5)	—	MD 2.35 lower (4.02 to 0.67 lower)	—	98	Moderate	Risk of bias (-1)
	no PI	1,009 (9)	—	MD 1.98 lower (2.8 to 1.15 lower)	—	98	Moderate	Risk of bias (-1)
Lund-Mackay CT score	Total	919 (9)	—	MD 2.56 lower (3.21 to 1.92 lower)	—	96	Moderate	Risk of bias (-1)
Subgroup 1	children	301 (3)	—	MD 5.25 lower (5.92 to 4.59 lower)	—	0	Moderate	Risk of bias (-1)
	adults	538 (5)	—	MD 1.47 lower (2.02 to 0.93 lower)	—	89	Moderate	Risk of bias (-1)
	both	80 (1)	—	MD 1.88 lower (2.04 to 1.72 lower)	—	NA	Low	Risk of bias (-1)
								Imprecision (-1)
Subgroup 2	PI	92 (1)	—	MD 1.63 lower (2.15 to 1.11 lower)	—	NA	Low	Risk of bias (-1)<
								Imprecision (-1)
	no PI	827 (8)	—	MD 2.71 lower (3.42 to 1.99 lower)	—	96	Moderate	Risk of bias (-1)
MTR (mm/min)	Total	1,358 (11)	—	MD 1.68 higher (1.24 to 2.11 higher)	—	97	Moderate	Risk of bias (-1)
Subgroup 1	children	397 (2)	—	MD 1.92 higher (1.81 to 2.04 higher)	—	0	Moderate	Risk of bias (-1)
	adults	647 (7)	—	MD 1.73 higher (1 to 2.46 higher)	—	98	Moderate	Risk of bias (-1)
	both	314 (2)	—	MD 1.17 higher (0.75 to 1.6 higher)	—	59	Moderate	Risk of bias (-1)
Subgroup 2	PI	562 (6)	—	MD 1.67 higher (0.84 to 2.5 higher)	—	97	Moderate	Risk of bias (-1)
	no PI	796 (5)	—	MD 1.68 higher (1.12 to 2.24 higher)	—	97	Moderate	Risk of bias (-1)
MCC (%)	Total	859 (7)	—	MD 9.05 higher (7.46 to 10.63 higher)	—	60	Moderate	Risk of bias (-1)
Subgroup 1	children	397 (2)	—	MD 8.75 higher (2.75 to 14.75 higher)	—	93	Moderate	Risk of bias (-1)
	adults	462 (5)	—	MD 9.18 higher (7.92 to 10.44 higher)	—	0	Moderate	Risk of bias (-1)
Subgroup 2	PI	362 (4)	—	MD 9.17 higher (7.76 to 10.59 higher)	—	0	Moderate	Risk of bias (-1)
	no PI	497 (3)	—	MD 8.89 higher (5.15 to 12.64 higher)	—	86	Moderate	Risk of bias (-1)
Recurrence rate	Total	680 (7)	204 per 1,000	57 per 1,000 (35–92)	RR 0.28 [0.17, 0.45]	0	Low	Risk of bias (-1)
								Imprecision (-1)
Subgroup 1	adults	448 (5)	205 per 1,000	53 per 1,000 (29–96)	RR 0.26 [0.14, 0.47]	0	Low	Risk of bias (-1)
								Imprecision (-1)
	both	232 (2)	204 per 1,000	65 per 1,000 (31–141)	RR 0.32 [0.15, 0.69]	0	Low	Risk of bias (-1)
								Imprecision (-1)
Subgroup 2	PI	115 (2)	286 per 1,000	46 per 1,000 (14–151)	RR 0.16 [0.05, 0.53]	0	Low	Risk of bias (-1)
								Imprecision (-1)
	no PI	565 (5)	190 per 1,000	59 per 1,000 (36–101)	RR 0.31 [0.19, 0.53]	0	Low	Risk of bias (-1)<
								Imprecision (-1)
Adverse events	Total	2,987 (28)	68 per 1,000	41 per 1,000 (31–56)	RR 0.61 [0.46, 0.83]	22	Low	Risk of bias (-1)
								Imprecision (-1)
Subgroup 1	children	318 (4)	25 per 1,000	6 per 1,000 (1–54)	RR 0.25 [0.03, 2.14]	NA	Low	Risk of bias (-1)
								Imprecision (-1)
	adults	2,409 (21)	75 per 1,000	47 per 1,000 (34–64)	RR 0.62 [0.45, 0.85]	30	Low	Risk of bias (-1)
								Imprecision (-1)
	both	260 (3)	54 per 1,000	38 per 1,000 (13–115)	RR 0.71 [0.24, 2.13]	NA	Low	Risk of bias (-1)
								Imprecision (-1)
Subgroup 2	PI	631 (8)	60 per 1,000	57 per 1,000 (31–103)	RR 0.94 [0.52, 1.70]	0	Low	Risk of bias (-1)
								Imprecision (-1)
	no PI	2,356 (20)	70 per 1,000	38 per 1,000 (27–53)	RR 0.54 [0.38, 0.76]	28	Low	Risk of bias (-1)
								Imprecision (-1)

CI, confidence interval; CT, computer tomography; HM, herbal medicine; MCC, mucociliary clearance rate; MD, mean difference; MTR, mucociliary transmission rate; NA, not applicable; PI, pattern identification; RCT, randomized controlled trial; RR, risk ratio; SNOT, sino-nasal outcome test; TER, total effective rate; VAS, visual analogue scale.

### 3.5 HM versus CT

When comparing HM with CT, TER based on the improvement of CRS symptoms was significantly higher in the HM group (11 studies, RR 1.20, 95% CI 1.11 to 1.31, *I* ^*2*^ = 63%), and, as a result of the subgroup analysis, the effect persisted in studies that enrolled children and adults separately. In addition, significant improvements were noted in VAS (5 studies, MD −0.73, 95% CI −0.97 to −0.5, *I* ^*2*^ = 81%), Lund-Kennedy endoscopy score (6 studies, MD −0.7, 95% CI −1.1 to −0.29, *I* ^*2*^ = 93%), and Lund-Mackay computed tomography score (6 studies, MD −1.31, 95% CI −2.41 to −0.22, *I* ^*2*^ = 94%) in the HM group post-treatment compared to the CT group (Table 1). A total of 5 studies (Jiang et al., 2012; Liu et al., 2012; Zhang, 2018; Liu, 2019; Xia et al., 2019) reported the occurrence of AEs, but all studies reported that no AEs occurred during the treatment period. After evaluating the publication bias with the Egger’s test, we revealed the risk of publication bias in the TER outcome (*p* = 0.001).

### 3.6 HM plus CT versus CT alone

When comparing HM plus CT versus CT alone, it was found that the severity of CRS symptoms, assessed by TER (58 studies, RR 1.23, 95% CI 1.20 to 1.25, *I* ^*2*^ = 27%) and VAS (11 studies, MD −2.66, 95% CI −3.25 to −2.07, *I* ^*2*^ = 98%), and health-related quality of life, assessed by SNOT-20 (4 studies, MD −2.76, 95% CI −3.04 to −2.48, *I* ^*2*^ = 89%), were significantly improved in the HM group. In addition, objective indicators, such as the Lund-Kennedy endoscopy score (14 studies, MD −2.11, 95% CI −2.83 to −1.38, *I* ^*2*^ = 98%) and the Lund-Mackay computed tomography score (9 studies, MD −2.56, 95% CI −3.21 to −1.92, *I* ^*2*^ = 96%), were significantly improved in the HM group. Further, nasal mucociliary function evaluated by MTR (11 studies, MD 1.68 mm/min, 95% CI 1.24 to 2.11, *I* ^*2*^ = 97%) and MCC (7 studies, MD 9.05%, 95% CI 7.46 to 10.63, *I* ^*2*^ = 60%) was significantly improved in the HM group. The recurrence rate of CRS (7 studies, RR 0.28, 95% CI 0.17 to 0.45, *I* ^*2*^ = 0%) and the number of AEs during the clinical trial period (28 studies, RR 0.61, 95% CI 0.46 to 0.83, *I* ^*2*^ = 22%) were also significantly lower in the HM group (Table 1). After assessing the publication bias with the Egger’s test, it was found that there was a risk of publication bias in the outcomes of TER (*p* < 0.001), VAS (*p* = 0.001), and Lund-Kennedy endoscopy score (*p* = 0.029) but no risk of publication bias in MTR (*p* = 0.377) or the incidence of AEs (*p* = 0.341).

### 3.7 HM versus No treatment

One study (Lin and Qiu, 2010) compared HM and no treatment, enrolling patients with CRS after functional endoscopic sinus surgery with pattern identification of retained heat invading the lung meridian, dampness-heat in the spleen, stomach, liver, and gallbladder, and dual deficiency of the lung-spleen. After administration of HM for 12 weeks, TER was significantly higher in the HM group (*p* < 0.01). Moreover, epithelialization time (*p* < 0.05), vesicle appearance time (*p* < 0.01), and vesicle duration time (*p* < 0.01) were significantly shorter in the HM group.

### 3.8 Quality of evidence

When comparing HM with placebo HM, the quality of evidence for the main findings was generally moderate due to the imprecision of the meta-analysis results. The quality of the main findings ranged from very low to moderate when comparing HM with CT and from low to moderate when comparing HM plus CT and CT alone. The main reasons for downgrading the quality of evidence were the high risk of bias in the included studies, inconsistency between studies, and imprecision due to small sample size or wide CIs (Table 1).

## 4 Discussion

### 4.1 Summary of evidence

The purpose of this systematic review was to comprehensively and critically review the empirical evidence on HM for CRS. In total, 80 RCTs were included in this review. Two previous systematic reviews that summarized the effects of HM on CRS treatment have been published (Guo et al., 2006; Anushiravani et al., 2018); however, these did not search local databases of East Asian countries where HM is actively used. Only 4 and 10 studies were included, respectively, and a sufficiently comprehensive search was not conducted. In addition, the quality of evidence for key findings that aid clinical decision-making was not evaluated in previous studies. Moreover, attempts to explain the clinical heterogeneity of patients, such as differences in age and pattern identification, were insufficient. In our review, the included studies typically compared HM with CT or HM plus CT with CT alone in patients with CRS. Some RCTs compared the placebo or no treatment groups with the HM group, and although these studies reported the results supporting the therapeutic benefit and safety of HM for CRS, the number of included studies was small. The meta-analysis results suggest that HM and HM plus CT were associated with significantly better improvement in CRS symptoms than CT alone. Additionally, HM and HM plus CT were associated with significantly better results than CT alone on objective indicators, including the Lund-Kennedy endoscopy score, the Lund-Mackay computed tomography score, and nasal mucociliary function assessed by MTR and MCC. Encouragingly, HM plus CT was associated with a significantly lower number of AEs compared to CT alone. Overall, the subgroup analysis revealed no differences in outcomes. This indicates no significant difference in the effect of HM as a monotherapy or adjuvant therapy on CRS according to age or whether pattern identification was applied.

However, the quality of evidence assessed using the GRADE ranged from very low to moderate, and there were no studies with the quality of evidence ranked as high. The limited methodological quality of reviewed studies, which was the main reason for downgrading the quality of evidence, should also be emphasized. Although the included studies used appropriate random sequence generation, only two studies were assessed as having a low risk of bias in allocation concealment. In addition, since only 4 studies confirmed the blinding of participants and personnel, it is difficult to rule out the possibility of selection bias and placebo effects in most of the included studies. Moreover, since all studies did not mention the blinding of outcome assessors, they may be subject to detection bias. Consequently, the results of this review should be interpreted with caution since participants’ beliefs about their treatment in an HM trial may influence their complaints and even symptom improvement (Link et al., 2006). In addition, China is a country where HM based on traditional Chinese medicine is part of the national healthcare system, so there is a possibility that public perception and attitude toward HM are more favorable compared to other countries. Therefore, it is impossible to exclude the possibility that the placebo effect may have been exaggerated compared to other national groups. Finally, the potential publication bias for the main results detected with the Egger’s test is likely attributed to the very high proportion of positive findings associated with HM and HM plus CT that we identified in this review (Joober et al., 2012).

### 4.2 Implications of the results

Given that complementary and integrative treatments, including HM, are being given attention to overcome the limitations of CT for CRS (Rotenberg and Bertens, 2010; Mahboubi, 2018), it is important to critically evaluate the empirical evidence on HM as a potential candidate treatment strategy for CRS. This review revealed some limited clinical evidence that HM and HM combined with CT may be more effective and safer than strategies using CT alone in the treatment of CRS. However, the quality of the evidence needs to be further strengthened, and it is still assessed at an insufficient level of evidence to recommend a revision of the first-line treatment guidelines for CRS. However, the findings of this review highlight the unique therapeutic aspects of HM for CRS that may be reflected in the design of future clinical trials.

The individual herbs most commonly used in the studies reviewed here are *Magnolia denudata* Desr. [Magnoliaceae; Magnoliae Flos], *Xanthium strumarium* L. [Asteraceae; Xanthii Fructus], and *Angelica dahurica* Benth. et Hooker f. [Apiaceae; Angelicae Dahuricae Radix]. According to the EATM theory, it is believed that these three herbs eliminate the ‘wind evil,’ and in the pathological context, the wind, especially the external wind, is associated with colds, flu, and viruses (Dashtdar et al., 2016). Importantly, *Magnolia denudata* Desr. [Magnoliaceae; Magnoliae Flos] and *Xanthium strumarium* L. [Asteraceae; Xanthii Fructus] are frequently used in combination. The former is used to treat sinus congestion and headaches, and the latter is used to treat a viscous runny nose and headaches in CRS (Taw et al., 2013). *Magnolia denudata* Desr. [Magnoliaceae; Magnoliae Flos] has a long history of clinical use for managing rhinitis, CRS, and headache. Its anti-allergic, anti-inflammatory, and antibacterial activity have been reported (Shen et al., 2008). *Xanthium strumarium* L. [Asteraceae; Xanthii Fructus] has also been found to have a wide range of pharmacological effects, such as anti-allergic rhinitis, antitumor, anti-inflammatory, analgesic, insecticidal, antiparasitic, antioxidant, antibacterial, and antidiabetic effects (Fan et al., 2019). According to a recent real-world database study, *Angelica dahurica* Benth. et Hooker f. [Apiaceae; Angelicae Dahuricae Radix] is an herb commonly used for allergic rhinitis (Lu et al., 2020). In other words, the included studies often used some herbs with the effects of eliminating the external wind evil in the classical concept of EATM, as well as with anti-inflammatory, antiviral, and anti-allergic properties in the pharmacological concept of modern medicine.

On the other hand, some studies used herbs, including *Glycyrrhiza uralensis* Fisch. [Leguminosae; Glycyrrhizae Radix], *Poria cocos* (Schw.) Wolf [Polyporaceae; Poria (Hoelen)], *Astragalus membranaceus* Bunge [Leguminosae; Astragali Radix], *Platycodon grandiflorum* (Jacq.) A. DC. [Campanulaceae; Platycodi Radix], and *Atractylodes macrocepha-la* Koidz [Asteraceae; Atractylodis Rhizoma Alba], which are believed to have a nourishing tonic effect according to the EATM theory. These herbs promote immune system development, improve mucosal immune function, and have anti-inflammatory effects (Yue et al., 2012; Buchwald et al., 2020; Chen et al., 2020). As per the EATM theory, combining herbs that resist external pathogens with herbs that have immunomodulatory effects is an essential principle of HM composition, which has the advantage of managing CRS from a complex and multifactorial perspective (Taw et al., 2013). The frequently used herbs in the included studies suggest that the internal immunity of the human body was lowered and external wind invaded, thereby causing CRS. Meanwhile, in the included studies, TER, which reflects the improvement of clinical symptoms, computed tomography scan, and nasal mucociliary function were reviewed as outcomes of interest. Although not the outcomes of interest in our review, some included studies reported secondary markers related to immunity and inflammation, such as interleukins, tumor necrosis factor-alpha (TNF-α), and C-reactive protein (CRP). In particular, TNF-α and CRP are significantly lower after treatment in the HM group (Chen et al., 2016; Hou, 2018; Liu et al., 2018; Huang, 2020; Liu and Xu, 2020; Ma et al., 2020; Yang, 2020; Li, 2021; Song et al., 2021). Given that immunomodulation is currently attracting attention in CT for CRS (Lavigne and Lee, 2018) and that herbs with immunomodulatory effects are already frequently used in HM studies (e.g., *Astragalus membranaceus* Bunge [Leguminosae; Astragali Radix]), future clinical CRS studies on HM may consider focusing on the immunomodulatory effect as the primary trial outcome.

Another interesting observation is the potential effect of HM on the recurrence of CRS. According to a 12-years follow-up study of CRS patients with nasal polyps after endoscopic sinus surgery, the recurrence rate of CRS reached 78.9% (Calus et al., 2019). In other long-term follow-up data from the same population, a recurrence of CRS was found in 40.1% of patients. Additionally, high eosinophilic infiltration and high interleukin-5 expression have been associated with a higher rate of CRS recurrence, suggesting a potential relationship with inflammation and immunomodulation (Rosati et al., 2020). In this meta-analysis, based on 7 RCTs, it was found that the recurrence rate of CRS was significantly lower in the HM plus CT group compared to the CT alone group. Some HM modalities with anti-inflammatory and immunomodulatory effects for CRS, which has a high recurrence rate even after surgical treatment, can be considered a promising candidate for adjuvant therapy. Its practical value should be proven in future clinical studies.

We did not find any significant differences in outcome between subgroups. There is evidence that the histopathological characteristics of CRS may differ depending on patient age (Mahdavinia and Grammer, 2013; Snidvongs et al., 2020); however, another study has reported that treatment outcomes do not differ significantly depending on the age of the participants with CRS (Devillier et al., 2016). Due to the limited number of studies included and weak quality of evidence of the findings, it was not possible to estimate the differences between the subgroups reliably for some outcomes. Therefore, differences in the effectiveness of HM as a monotherapy or adjuvant therapy for CRS according to age or pattern identification remain an open question.

This systematic review provides an up-to-date comprehensive review of the effectiveness and safety of HM for CRS. However, the following limitations should be considered. Firstly, the methodological quality of the included studies was generally low. In particular, compliance with allocation concealment and blinding of participants and personnel has not been confirmed for the reviewed studies, so the results could be subject to selection bias and placebo effects. In addition, the poor methodological quality of the included studies was the main reason for weakening the quality of the evidence for the meta-analysis results. This highlights the need for high-quality, rigorous RCTs in this field. Secondly, the publication bias found in the results of major meta-analyses suggests that the effectiveness and safety of HM as monotherapy or adjuvant therapy should be interpreted with caution. Most of the included studies were conducted in China, which may be attributed to publication bias (Vickers et al., 1998). Considering that EATM is used not only in China but also in other East Asian countries, such as Korea, Japan, and Taiwan, future studies in this field should recruit participants of different nationalities. Third, in most of the meta-analysis results, we found that heterogeneity was not explained by subgroup analysis. This may be due to unexamined subgroup analysis or differences in the interventions used (HM), small-study effects (IntHout et al., 2015), unknown causes, or chance.

## 5 Conclusion

This review revealed some limited clinical evidence that HM or HM combined with CT may be more effective and safer than CT alone in the treatment of CRS. However, the methodological quality of the included studies was generally low, and future high-quality RCTs need to be conducted.

## Data Availability

The original contributions presented in the study are included in the article/Supplementary Material, further inquiries can be directed to the corresponding author.

## Abbreviations

AMED, Allied and Complementary Medicine Database; CENTRAL, Cochrane Central Register of Controlled Trials; CIM, Complementary and Integrative Medicine; CNKI, China National Knowledge Infrastructure; CQVIP, Chongqing VIP; EATM, East Asian Traditional Medicine; KISS, Koreanstudies Information Service System; KMbase, Korean Medical Database; OASIS, Oriental Medicine Advanced Searching Integrated System.
