# Supplementary material for: Herbal medicine for the treatment of chronic rhinosinusitis: A systematic review and meta-analysis
Source: Front Pharmacol. 2022 Jul 18;13:908941. doi: 10.3389/fphar.2022.908941 (PMC9341451; doi:10.3389/fphar.2022.908941)
Supplement: Supplementary file 1 [file Table5.PDF]

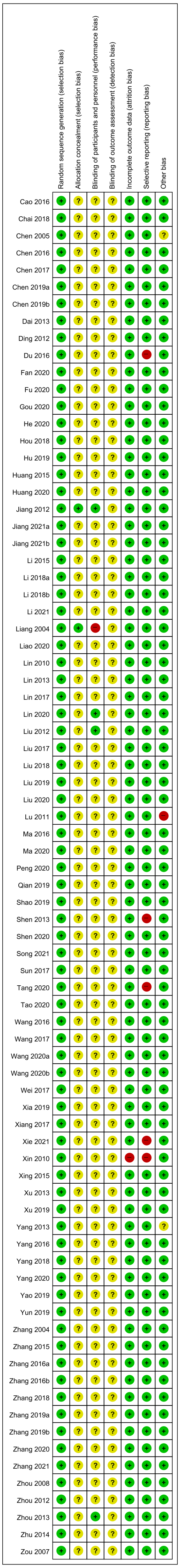

Random sequence generation (selection bias)
Allocation concealment (selection bias)
Blinding of participants and personnel (performance bias)
Blinding of outcome assessment (detection bias)
Incomplete outcome data (attrition bias)
Selective reporting (reporting bias)
Other bias
Cao 2016
Chai 2018
Chen 2005
Chen 2016
Chen 2017
Chen 2019a
Chen 2019b
Dai 2013
Ding 2012
Du 2016
Fan 2020
Fu 2020
Gou 2020
He 2020
Hou 2018
Hu 2019
Huang 2015
Huang 2020
Jiang 2012
Jiang 2021a
Jiang 2021b
Li 2015
Li 2018a
Li 2018b
Li 2021
Liang 2004
Liao 2020
Lin 2010
Lin 2013
Lin 2017
Lin 2020
Liu 2012
Liu 2017
Liu 2018
Liu 2019
Liu 2020
Lu 2011
Ma 2016
Ma 2020
Peng 2020
Qian 2019
Shao 2019
Shen 2013
Shen 2020
Song 2021
Sun 2017
Tang 2020
Tao 2020
Wang 2016
Wang 2017
Wang 2020a
Wang 2020b
Wei 2017
Xia 2019
Xiang 2017
Xie 2021
Xin 2010
Xing 2015
Xu 2013
Xu 2019
Yang 2013
Yang 2016
Yang 2018
Yang 2020
Yao 2019
Yun 2019
Zhang 2004
Zhang 2015
Zhang 2016a
Zhang 2016b
Zhang 2018
Zhang 2019a
Zhang 2019b
Zhang 2020
Zhang 2021
Zhou 2008
Zhou 2012
Zhou 2013
Zhu 2014
Zou 2007
