# Supplementary material for: Herbal medicine for the treatment of chronic rhinosinusitis: A systematic review and meta-analysis
Source: Front Pharmacol. 2022 Jul 18;13:908941. doi: 10.3389/fphar.2022.908941 (PMC9341451; doi:10.3389/fphar.2022.908941)
Supplement: Supplementary file 2 [file Table1.DOCX]

**Supplement 1**. Search terms used in each database and results

**MEDLINE via PubMed**

|  | Searches | Results |
| --- | --- | --- |
| #1 | Sinusitis[MH] OR sinusitis[TIAB] OR rhinosinusitis[TIAB] OR “rhino sinusitis”[TIAB] OR nasosinusitis[TIAB] OR “naso sinusitis”[TIAB] OR “nasal sinusitis”[TIAB] OR pansinusitis[TIAB] OR “pan sinusitis”[TIAB] OR ethmoiditis[TIAB] OR antritis[TIAB] OR sphenoiditis[TIAB] OR “sinus infection”[TIAB] OR “paranasal inflammation”[TIAB] OR “paranasal infection”[TIAB] OR (inflammation NEAR sinus) | 31,679 |
| #2 | “Plants, Medicinal”[MH] OR “Drugs, Chinese Herbal”[MH] OR “Medicine, Chinese Traditional”[MH] OR “Medicine, Kampo”[MH] OR “Medicine, Korean Traditional”[MH] OR “Herbal Medicine”[MH] OR “traditional Korean medicine”[TIAB] OR “traditional Chinese medicine”[TIAB] OR “traditional oriental medicine”[TIAB] OR “Kampo medicine”[TIAB] OR herb*[TIAB] OR decoction*[TIAB] OR botanic*[TIAB] | 228,910 |
| #3 | “randomized controlled trial”[PT] OR “controlled clinical trial”[PT] OR randomized[TIAB] OR placebo[TIAB] OR “drug therapy”[SH] OR randomly[TIAB] OR trial[TIAB] OR groups[TIAB] | 5,125,545 |
| #4 | animals[MH] NOT humans[MH] | 4,856,894 |
| #5 | #1 AND #2 AND #3 NOT #4 | **102** |

**EMBASE via Elsevier**

|  | Searches | Results |
| --- | --- | --- |
| #1 | sinusitis/exp OR sinusitis:ab,ti OR rhinosinusitis/exp OR rhinosinusitis:ab,ti OR ‘rhino sinusitis’:ab,ti OR nasosinusitis:ab,ti OR ‘naso sinusitis’:ab,ti OR ‘nasal sinusitis’:ab,ti OR  pansinusitis:ab,ti OR ‘pan sinusitis’:ab,ti OR ethmoiditis:ab,ti OR antritis:ab,ti OR sphenoiditis:ab,ti OR ‘sinus infection’:ab,ti OR ‘paranasal inflammation’:ab,ti OR ‘paranasal infection’:ab,ti OR ‘sinus inflammation’:ab,ti | 54,974 |
| #2 | ‘medicinal plant’/exp OR ‘medicinal plant’:ab,ti OR ‘herbaceous agent’/exp OR ‘herbaceous agent’:ab,ti OR ‘chinese medicine’/exp OR ‘chinese medicine’:ab,ti OR ‘kampo medicine’/exp OR ‘kampo medicine’:ab,ti OR ‘kampo medicine (drug)’/exp OR ‘kampo medicine (drug)’:ab,ti OR ‘korean medicine’/exp OR ‘korean medicine’:ab,ti OR ‘herbal medicine’/exp OR ‘herbal medicine’:ab,ti OR ‘oriental medicine’/exp OR ‘oriental medicine’:ab,ti OR herb/exp OR herb*:ab,ti OR decoction*:ab,ti OR botanic*:ab,ti | 460,649 |
| #3 | 'crossover procedure':de OR 'double-blind procedure':de OR 'randomized controlled trial':de OR 'single-blind procedure':de OR (random* OR factorial* OR crossover* OR cross NEXT/1 over* OR placebo* OR doubl* NEAR/1 blind* OR singl* NEAR/1 blind* OR assign* OR allocat* OR volunteer*):de,ab,ti | 2,772,428 |
| #4 | #1 AND #2 AND #3 | **162** |

**CENTRAL**

|  | Searches | Results |
| --- | --- | --- |
| #1 | MeSH descriptor: [Sinusitis] explode all trees | 1,055 |
| #2 | (sinusitis OR rhinosinusitis OR “rhino sinusitis” OR nasosinusitis OR “naso sinusitis” OR “nasal sinusitis” OR pansinusitis OR “pan sinusitis” OR ethmoiditis OR antritis OR sphenoiditis OR “sinus infection” OR “paranasal inflammation” OR “paranasal infection” OR "sinus inflammation"):ti,ab,kw | 3,851 |
| #3 | #1 OR #2 | 3,851 |
| #4 | MeSH descriptor: [Plants, Medicinal] explode all trees | 946 |
| #5 | MeSH descriptor: [Drugs, Chinese Herbal] explode all trees | 3,665 |
| #6 | MeSH descriptor: [Medicine, Chinese Traditional] explode all trees | 1,222 |
| #7 | MeSH descriptor: [Medicine, Kampo] explode all trees | 46 |
| #8 | MeSH descriptor: [Medicine, Korean Traditional] explode all trees | 33 |
| #9 | MeSH descriptor: [Herbal Medicine] explode all trees | 63 |
| #10 | (“traditional Korean medicine” OR “traditional Chinese medicine” OR “traditional oriental medicine” OR “Kampo medicine” OR herb* OR decoction* OR botanic*):ti,ab,kw | 18,892 |
| #11 | #4 OR #5 OR #6 OR #7 OR #8 OR #9 OR #10 | 20,045 |
| #12 | (#3 AND #11) in Trials | **87** |

**AMED via EBSCO**

|  | Searches | Results |
| --- | --- | --- |
| #1 | Sinusitis[SU] OR sinusitis[TX] OR rhinosinusitis[TX] OR “rhino sinusitis”[TX] OR nasosinusitis[TX] OR “naso sinusitis”[TX] OR “nasal sinusitis”[TX] OR pansinusitis[TX] OR “pan sinusitis”[TX] OR ethmoiditis[TX] OR antritis[TX] OR sphenoiditis[TX] OR “sinus infection”[TX] OR “paranasal inflammation”[TX] OR “paranasal infection”[TX] OR “sinus inflammation”[TX] | 177 |
| #2 | “Plants, Medicinal”[SU] OR “Drugs, Chinese Herbal”[SU] OR “Medicine, Chinese Traditional”[SU] OR “Medicine, Kampo”[SU] OR “Medicine, Korean Traditional”[SU] OR “Herbal Medicine”[SU] OR “traditional Korean medicine”[TX] OR “traditional Chinese medicine”[TX] OR “traditional oriental medicine”[TX] OR “Kampo medicine”[TX] OR herb*[TX] OR decoction*[TX] OR botanic*[TX] | 34,774 |
| #3 | #1 AND #2 | **39** |

**CINAHL via EBSCO**

|  | Searches | Results |
| --- | --- | --- |
| #1 | Sinusitis[MH] OR sinusitis[TX] OR rhinosinusitis[TX] OR “rhino sinusitis”[TX] OR nasosinusitis[TX] OR “naso sinusitis”[TX] OR “nasal sinusitis”[TX] OR pansinusitis[TX] OR “pan sinusitis”[TX] OR ethmoiditis[TX] OR antritis[TX] OR sphenoiditis[TX] OR “sinus infection”[TX] OR “paranasal inflammation”[TX] OR “paranasal infection”[TX] OR “sinus inflammation”[TX] | 9,702 |
| #2 | “Plants, Medicinal”[MH] OR “Drugs, Chinese Herbal”[MH] OR “Medicine, Chinese Traditional”[MH] OR “Medicine, Kampo”[MH] OR “Medicine, Korean Traditional”[MH] OR “Herbal Medicine”[MH] OR “traditional Korean medicine”[TX] OR “traditional Chinese medicine”[TX] OR “traditional oriental medicine”[TX] OR “Kampo medicine”[TX] OR herb*[TX] OR decoction*[TX] OR botanic*[TX] | 120,723 |
| #3 | #1 AND #2 | **687** |

**OASIS**

|  | Searches | Results |
| --- | --- | --- |
| #1 | (부비동염\|비부비동염\|부비강염\|축농증\|코곁굴염) (한약\|약초\|본초\|탕\|환\|산) | **2** |

**KMbase**

|  | Searches | Results |
| --- | --- | --- |
| #1 | (((([ALL=부비동염] OR [ALL=비부비동염]) OR [ALL=부비강염]) OR [ALL=축농증]) OR [ALL=코곁굴염]) | 525 |
| #2 | ((((([ALL=한약] OR [ALL=약초]) OR [ALL=본초]) OR [ALL=탕]) OR [ALL=환]) OR [ALL=산]) | 113,659 |
| #3 | #1 AND #2 | **205** |

**KISS**

|  | Searches | Results |
| --- | --- | --- |
| #1 | 초록=(부비동염\|비부비동염\|부비강염\|축농증\|코곁굴염) AND 초록=(한약\|약초\|본초\|탕\|환\|산) | **106** |

**ScienceON**

|  | Searches | Results |
| --- | --- | --- |
| #1 | (부비동염\|비부비동염\|부비강염\|축농증\|코곁굴염) (한약\|약초\|본초\|탕\|환\|산) | **104** |

**RISS**

|  | Searches | Results |
| --- | --- | --- |
| #1 | (부비동염\|비부비동염\|부비강염\|축농증\|코곁굴염) (한약\|약초\|본초\|탕\|환\|산) | **37** |

**CNKI**

|  | Searches | Results |
| --- | --- | --- |
| #1 | (SU='鼻窦炎'+'窦炎'+'鼻渊'+‘脑漏'+‘鼻道炎'+'鼻痈'+'蓄脓症'+'蓄脓'+'副鼻腔炎') AND (SU='中医药'+'中医'+'中西医结合'+'中药'+'汤'+'丸'+'散'+'饮'+'颗粒'+'胶囊'+'自拟') AND (SU='随机'+'对照'+'随意'+'试验'+'安慰') | **591** |

**Wanfang data**

|  | Searches | Results |
| --- | --- | --- |
| #1 | (主题:鼻窦炎 OR 主题:窦炎 OR 主题:鼻渊 OR 主题:脑漏 OR 主题:鼻道炎 OR 主题:鼻痈 OR 主题:蓄脓症 OR 主题:蓄脓 OR 主题:副鼻腔炎) AND (主题:中医药 OR 主题:中医 OR 主题:中西医结合 OR 主题:中药 OR 主题:汤 OR 主题:丸 OR 主题:散 OR 主题:饮 OR 主题:颗粒 OR 主题:胶囊 OR 主题:自拟) AND (主题:随机 OR 主题:对照 OR 主题:随意 OR 主题:试验 OR 主题:安慰) | **2,118** |

**VIP.**

|  | Searches | Results |
| --- | --- | --- |
| #1 | (M=(鼻窦炎 OR 窦炎 OR 鼻渊 OR 脑漏 OR 鼻道炎 OR 鼻痈 OR 蓄脓症 OR 蓄脓 OR 副鼻腔炎) AND M=(中医药 OR 中医 OR 中西医结合 OR 中药 OR 汤 OR 丸 OR 散 OR 饮 OR 颗粒 OR 胶囊 OR 自拟) AND M=(随机 OR 对照 OR 随意 OR 试验 OR 安慰)) | **60** |

**CiNii**

|  | Searches | Results |
| --- | --- | --- |
| #1 | (副鼻腔炎 OR 蓄膿症 OR じょうみゃくどうえん OR せいふくびくうえん OR 上顎洞炎 OR じょうがくどうえん OR 前頭洞炎 OR ぜんとうどうえん OR 静脈洞炎) AND (漢方薬 OR ハーブ OR 散 OR 汤 OR 丸) AND (ランダム化比較試験 OR 対照臨床試験 OR ランダム OR 無作為 OR 対照 OR 試験 OR 偽薬) | **11** |
