# Supplementary material for: Herbal medicine for the treatment of chronic rhinosinusitis: A systematic review and meta-analysis
Source: Front Pharmacol. 2022 Jul 18;13:908941. doi: 10.3389/fphar.2022.908941 (PMC9341451; doi:10.3389/fphar.2022.908941)
Supplement: Supplementary file 3 [file Table2.DOCX]

**Supplement 2. Excluded studies after full-text reviewing**

**1. not randomized controlled trial (n = 94)**

Liu, Y. C. (1986). [Observation on clinical experiments in the treatment of chronic nasal sinusitis with ping zhi tablets]. *Zhong Xi Yi Jie He Za Zhi*, *6*(10), 596-598, 580.

Su, C. Z. (1984). [Treatment of nasal sinusitis with qin jie tang and changes in IgG levels]. *Zhong Xi Yi Jie He Za Zhi*, *4*(12), 730-731.

耿亚, 田宏俊, & 马永明. (2013). 菊芩苍耳合剂治疗急性鼻窦炎临床研究

Clinical research on treating acute sinusitis by the Juqin Xanthium mixture. *中医临床研究*(20), 11-12. https://doi.org/10.3969/j.issn.1674-7860.2013.20.005

郭吉健. (2012). 中药配合盐水清洗鼻法治疗慢性鼻窦炎的效果观察. *中国卫生产业*, *9*(31), 174.

郭艳梅, 李永伟, 孙麦青, 李泳文, 牛小斌, & 刘严严. (2019). 通窍鼻炎丸治疗慢性鼻窦炎疗效及ECP、TIgE、hs-CRP等因子影响研究

Effect of Tongqiao Rhinitis Pill on Levels of Inflammatory Factors, Eosinophil Cationic Protein(ECP) in Serum and Nasal Cavity in Patients with Chronic Sinusitis. *中华中医药学刊*, *37*(2), 406-409. https://doi.org/10.13193/j.issn.1673-7717.2019.02.037

弓玲玲. (2019). 清窦汤治疗鼻窦炎的临床观察探讨. *首都食品与医药*, *26*(19), 192-193. https://doi.org/10.3969/j.issn.1005-8257.2019.19.161

宫静. (2017). 中医护理干预对慢性鼻窦炎围手术期的效果. *内蒙古中医药*, *36*(14), 156-157. https://doi.org/10.3969/j.issn.1006-0979.2017.14.153

金佳益. (2015). *清肺通窍方对慢性鼻窦炎术后鼻腔黏膜形态和功能恢复的疗效观察* [硕士, 南京中医药大学]. https://d.wanfangdata.com.cn/thesis/ChJUaGVzaXNOZXdTMjAyMTA1MTkSCFkyODc1OTkzGgg0bjR0d3czdg%3D%3D

金攀, & 姚士红. (2017). 鼻渊通窍颗粒联合曲安奈德鼻喷雾剂治疗慢性鼻-鼻窦炎的临床价值探究

Research on Clinical Value of Biyuan Tongqiao Granule Combined With Triamcinolone Acetonide Nasal Spray in the Treatment of Chronic Rhinosinusitis. *中国卫生标准管理*, *8*(2), 111-112. https://doi.org/10.3969/j.issn.1674-9316.2017.02.070

唐成定, & 朱志芳. (2009). 银花通窍鼻炎汤治疗慢性鼻窦炎50例. *河南中医*, *29*(11), 1101-1102. https://d.wanfangdata.com.cn/periodical/ChlQZXJpb2RpY2FsQ0hJTmV3UzIwMjEwNjE2Eg1obnp5MjAwOTExMDM2GghjZzR0MWxpMQ%3D%3D

唐爱华. (2017). 鼻渊汤随证加减联合罗红霉素治疗急性鼻窦炎的疗效和安全性分析. *中国中西医结合耳鼻咽喉科杂志*, *25*(06), 426-428+435.

戴飞. (2013). *中药逐渊汤与克拉霉素对慢性鼻-鼻窦炎术后疗效的对比观察* [硕士, 复旦大学]. https://d.wanfangdata.com.cn/thesis/ChJUaGVzaXNOZXdTMjAyMTA1MTkSCFkyNzAxNTg2GghjajlqNnY2Nw%3D%3D

罗海霞. (2009). *中医辨证施治综合治疗儿童慢性鼻窦炎优势初探* [硕士, 浙江中医药大学]. https://d.wanfangdata.com.cn/thesis/ChJUaGVzaXNOZXdTMjAyMTA1MTkSB0Q3ODgwMTAaCGx0bXJvOGI2

刘可普. (2014). *鼻渊合剂联合大环内酯类抗生素对术后鼻窦黏膜上皮化的影响* [硕士, 南京中医药大学]. https://d.wanfangdata.com.cn/thesis/ChJUaGVzaXNOZXdTMjAyMTA1MTkSCFkyNjczNTI0GghsdG1ybzhiNg%3D%3D

刘国新, & 蔡可可. (2018). 千金苇茎汤联合西药治疗鼻窦炎(痰瘀阻滞)随机平行对照研究. *实用中医内科杂志*, *32*(10), 60-63.

刘枚. (2012). 慢性上颌窦炎临床治疗效果观察. *中国中医药咨讯*, *4*(5), 276. https://d.wanfangdata.com.cn/periodical/ChlQZXJpb2RpY2FsQ0hJTmV3UzIwMjEwNjE2EhB6Z3p5eXp4MjAxMjA1MjQyGghsdG1ybzhiNg%3D%3D

刘文君, 刘芳琼, 黄凌飞, & 李湘宇. (2006). 中西医结合治疗鼻内镜术后32例疗效观察. *湖南中医杂志*, *22*(5), 27,29. https://doi.org/10.3969/j.issn.1003-7705.2006.05.016

刘春宜. (2016). 鼻窦炎应用中西医结合治疗的有效性探究. *中西医结合心血管病电子杂志*, *4*(30), 172.

李道广. (2011). 中西医结合保守治疗儿童慢性鼻窦炎60例. *健康必读（下旬刊）*(11), 325-325,312. https://d.wanfangdata.com.cn/periodical/ChlQZXJpb2RpY2FsQ0hJTmV3UzIwMjEwNjE2Eg9qa2JkLXgyMDExMTE0NjUaCGNnNHQxbGkx

李头清. (2016). 中西医结合治疗慢性鼻窦炎30例. *现代中医药*, *36*(03), 39-40.

李亮. (2019). 黄芩汤合苍耳子散为主对慢性鼻窦炎的应用价值分析. *中国保健营养*, *17*(17), 354-355. https://d.wanfangdata.com.cn/periodical/ChlQZXJpb2RpY2FsQ0hJTmV3UzIwMjEwNjE2EhJ6Z2JqeXkta3AyMDE5MTc0ODcaCGNqOWo2djY3

李蓓, & 洪钱江. (2013). *慢性鼻窦炎中西医综合干预与疗效的临床研究* 中国中西医结合学会耳鼻咽喉科专业委员会第十三次学术会议暨浙江省中西医结合学会耳鼻咽喉科专业委员会第十一次学术会议论文集, 浙江嘉兴. https://d.wanfangdata.com.cn/conference/ChZDb25mZXJlbmNlTmV3UzIwMjEwNTIxEgc4MjU1NTg1GghsdG1ybzhiNg%3D%3D

李斯斯, 张凌浩, 张泉波, 张悦, & 贾明辉. (2018). 苍耳子鼻炎胶囊联合改良鼻腔冲洗方案对急性鼻窦炎患者临床疗效的影响

Effect of Xanthium sibiricum capsule combined with modified nasal irrigation on clinical efficacy in patients with acute sinusitis. *中国中西医结合急救杂志*, *25*(4), 434-436. https://doi.org/10.3969/j.issn.1008-9691.2018.04.024

李松华. (2017). 中药联合鼻内窥镜手术治疗慢性鼻窦炎对照观察. *临床心身疾病杂志*, *23*(3). http://qikan.cqvip.com/Qikan/Article/Detail?id=672533453

李青松. (2019). 自拟醒窍汤联合鼻内镜手术治疗慢性鼻-鼻窦炎患者的疗效分析. *黑龙江中医药*, *48*(06), 78-79.

李惠君, 吕楠, & 王晓梅. (2017). 不同中医护理干预方式对鼻窦炎术后患者疼痛的影响评价. *母婴世界*(20), 134. https://doi.org/10.3969/j.issn.1671-2242.2017.20.129

林良志. (2014). 鼻渊汤配合西药治疗儿童慢性鼻窦炎效果观察. *中国乡村医药*, *21*(12), 77-78.

林玲玲, 林少雄, & 程耿斌. (2013). 慢性鼻-鼻窦炎内窥镜鼻窦手术术后中医辨证治疗的主客观疗效初探

Discussion on syndrome differentiation of traditional Chinese medicine's objective and subjective curative effect of chronic rhinosinusitis after endoscopic sinus surgery. *国际医药卫生导报*, *19*(22), 3383-3386. https://doi.org/10.3760/cma.j.issn.1007-1245.2013.22.001

马少民, 马海艳, 梁续伟, 孟宇青, & 栾玉. (2018). 鼻渊通窍颗粒联合西药治疗慢性鼻—鼻窦炎的临床疗效及对患儿生活质量的影响. *贵州医药*, *42*(11), 1327-1328.

苗莉, 侯瑾, & 王霞. (2018). 鼻渊舒口服液联合罗红霉素胶囊治疗慢性鼻窦炎的疗效及安全性. *世界最新医学信息文摘*, *18*(15), 124+127.

房耿浩. (2015). 解郁祛湿建中汤治疗湿热型慢性鼻窦炎临床观察. *新中医*, *47*(5), 225-226. https://doi.org/10.13457/j.cnki.jncm.2015.05.107

樊辉, & 兰家群. (2020). 鼻内镜手术结合玉屏风颗粒治疗慢性鼻窦炎伴鼻息肉的疗效及对免疫功能的影响研究. *康颐*(2), 184. https://d.wanfangdata.com.cn/periodical/ChlQZXJpb2RpY2FsQ0hJTmV3UzIwMjEwNjE2EhpRS0JKQkQyMDIwMjAyMTAzMDUwMDAwNTU5ORoIY2o5ajZ2Njc%3D

谢坤. (2017). 鼻渊通窍颗粒联合曲安奈德鼻喷雾剂治疗慢性 鼻-鼻窦炎的临床疗效及安全性. *心理医生*, *23*(11), 127-128. https://d.wanfangdata.com.cn/periodical/ChlQZXJpb2RpY2FsQ0hJTmV3UzIwMjEwNjE2Eg94bHlzLXgyMDE3MTEwOTcaCGNqOWo2djY3

徐端, & 李旭. (2009). 中西医结合治疗儿童慢性鼻窦炎53例临床观察. *辽宁中医杂志*, *36*(4), 596-597. https://d.wanfangdata.com.cn/periodical/ChlQZXJpb2RpY2FsQ0hJTmV3UzIwMjEwNjE2Eg9sbnp5enoyMDA5MDQwNTcaCGNnNHQxbGkx

石姗妮, & 郭瑞红. (2004). 中西医结合治疗儿童慢性鼻窦炎. *中原医刊*, *31*(18), 60. https://doi.org/10.3760/cma.j.issn.1674-4756.2004.18.062

石越颖. (2016). 半夏天麻汤对慢性鼻窦炎患者术后的影响

Effects of Banxia Tianma Decoction on Patients with Chronic Sinusitis. *光明中医*, *31*(12), 1687-1689. https://doi.org/10.3969/j.issn.1003-8914.2016.12.005

宋翠叶. (2014). 中西医结合治疗老年慢性鼻窦炎. *光明中医*(6), 1269-1270. https://doi.org/10.3969/j.issn.1003-8914.2014.06.081

宋红梅, 龚小丽, & 熊大经. (2004). 健脾益气联合鼻渊舒口服液治疗儿童慢性鼻窦炎. *四川中医*, *22*(2), 70-71. https://doi.org/10.3969/j.issn.1000-3649.2004.02.049

岳延琴. (2018). 中西医结合在儿童急性鼻窦炎治疗中的疗效研究. *医药前沿*, *8*(5), 178. https://doi.org/10.3969/j.issn.2095-1752.2018.05.144

安永卿. (2012). 中药汤剂治疗慢性鼻窦炎对照研究

Traditional Chinese Medicine Decoction Treatment of Chronic Sinusitis in Case-control Study. *实用中医内科杂志*(17), 51,53. https://d.wanfangdata.com.cn/periodical/ChlQZXJpb2RpY2FsQ0hJTmV3UzIwMjEwNjE2Eg5RSzIwMTIwNjEzMDIyNRoIY2o5ajZ2Njc%3D

杨九一. (2012). *功能性鼻内窥镜术后应用三和通窍开玄汤近期疗效观察研究* [硕士, 成都中医药大学].

杨洁. (2019). 用加味温胆汤治疗胆腑郁热型急性鼻-鼻窦炎的效果观察. *当代医药论丛*, *17*(10), 208-209. https://d.wanfangdata.com.cn/periodical/ChlQZXJpb2RpY2FsQ0hJTmV3UzIwMjEwNjE2Eg9kZHl5bGMyMDE5MTAxNTMaCGNqOWo2djY3

杨迪. (2019). 鼻渊通窍颗粒治疗急性鼻窦炎的疗效观察. *中国医药指南*, *17*(07), 162-163.

余凤慈, 梁淑贞, 邱晓云, & 李振安. (2014). 中药健脾疗法治疗儿童慢性鼻窦炎的临床效果. *中国中西医结合耳鼻咽喉科杂志*, *22*(02), 130-131.

吴立明, 郝芬兰, & 程晓卫. (2006). 三花鼻康胶囊治疗慢性鼻窦炎400例. *陕西中医*, *27*(8), 915-916. https://doi.org/10.3969/j.issn.1000-7369.2006.08.013

吴随记, & 吴学敬. (2008). 清窦饮治疗鼻渊症144例. *陕西中医*, *29*(7), 867. https://doi.org/10.3969/j.issn.1000-7369.2008.07.084

敖勇, & 牛良君. (2019). 辛芳鼻炎胶囊联合罗红霉素治疗慢性鼻窦炎的临床研究. *现代药物与临床*, *34*(05), 1418-1421.

吴印娟. (2017). 论述鼻渊舒口服液联合罗红霉素胶囊治疗慢性鼻窦炎的疗效及安全性. *世界最新医学信息文摘*, *17*(36), 157+159.

阮杰坚. (2019). 鼻渊通窍颗粒联合曲安奈德鼻喷雾剂治疗慢性鼻-鼻窦炎的效果分析. *黑龙江中医药*, *48*(03), 88-89.

王江, 王易, & 王庆. (2018). 鼻渊通窍颗粒联合曲安奈德鼻喷雾剂治疗慢性鼻-鼻窦炎的临床价值. *饮食保健*, *5*(40), 30-31. https://doi.org/10.3969/j.issn.2095-8439.2018.40.028

王勇, & 贾乐. (2016). 慢性鼻窦炎中医治疗与内窥镜手术的比较. *包头医学院学报*, *32*(10), 87-89.

王浩, & 张敏. (2021). 清窦汤治疗鼻窦炎的临床研究

Clinical observation on treating nasosinusitis with the Qingdou decoction. *中医临床研究*, *13*(1), 99-101. https://doi.org/10.3969/j.issn.1674-7860.2021.01.034

熊静, 周振峰, & 吴婷. (2017). 龙胆通窍丸对湿热型慢性鼻窦炎患者血清IL-6和IL-8水平的影响

Study on the effect of Longdan Tongqiao pills on serum IL-6 and IL-8 level in patients with chronic sinusitis of damp heat type. *中国中西医结合耳鼻咽喉科杂志*, *25*(4), 241-243,249. https://doi.org/10.16542/j.cnki.issn.1007-4856.2017.04.001

袁龙, & 贾全凡. (2016). 葛根汤加减联合鼻内镜手术治疗难治性鼻窦炎临床观察

Clinical Observation of Using Modified Gegen Decoction Combined with Nasal Endoscopic Surgery in the Treatment of Refractory Sinusitis. *四川中医*, *34*(12), 106-108. https://d.wanfangdata.com.cn/periodical/ChlQZXJpb2RpY2FsQ0hJTmV3UzIwMjEwNjE2Eg1zY3p5MjAxNjEyMDQwGghjZzR0MWxpMQ%3D%3D

魏庭文, & 孙清琴. (2006). 左氧氟沙星注射液穿刺冲洗结合鼻渊汤治疗上颌窦炎. *河南职工医学院学报*, *18*(4), 310-311. https://doi.org/10.3969/j.issn.1008-9276.2006.04.031

张德玉, 张靖, & 冯念玲. (2007). 中西医结合治疗上颌窦炎. *中国伤残医学*, *15*(5), 51-52. https://doi.org/10.3969/j.issn.1673-6567.2007.05.040

张新日, 张伟, 邹广华, 龚建齐, 盛国强, & 徐红霞. (2019). 慢性鼻窦炎患者内镜术后中医辨证治疗的效果探讨. *医药前沿*, *9*(8), 224-225. https://d.wanfangdata.com.cn/periodical/ChlQZXJpb2RpY2FsQ0hJTmV3UzIwMjEwNjE2Eg55aXlxeTIwMTkwODE3OBoIY2c0dDFsaTE%3D

张卫东. (2015). 鼻炎康汤在胆腑郁热证慢性鼻-鼻窦炎治疗中的应用价值. *现代养生B*(10), 83-83. https://d.wanfangdata.com.cn/periodical/ChlQZXJpb2RpY2FsQ0hJTmV3UzIwMjEwNjE2Eg94ZHlzLWIyMDE1MTAwODAaCGx0bXJvOGI2

张海凤. (2014). 解析温阳通窍汤治疗慢性鼻窦炎Ⅱ型1期的临床应用. *医学信息*(13), 421-422. https://doi.org/10.3969/j.issn.1006-1959.2014.13.517

全梅玉, & 陈可香. (2017). 中西医结合治疗慢性鼻窦炎疗效分析. *海峡药学*, *29*(8), 76-77. https://doi.org/10.3969/j.issn.1006-3765.2017.08.033

田昌书. (2018). 用鼻渊舒口服液治疗儿童慢性鼻窦炎的效果探析. *当代医药论丛*, *16*(06), 181.

郑海明. (2018). 中西医结合治疗成年人慢性鼻窦炎临床研究. *新中医*, *50*(02), 72-74.

赵增强. (2018). 中西医结合治疗小儿慢性鼻窦炎临床观察. *糖尿病天地*, *15*(6), 126. https://doi.org/10.3969/j.issn.1672-7851.2018.06.120

赵倩倩, & 梁乐平. (2020). 鼻炎苍菊饮联合头孢拉定对感染性鼻窦炎患者症状及免疫功能的影响

Effect of Cangju decoction combined with Cefradine on symptoms and immune function in patients with infectious sinusitis. *海南医学*, *31*(14), 1797-1799. https://doi.org/10.3969/j.issn.1003-6350.2020.14.008

曹晓辉. (2019). 清热通窍汤联合西药治疗慢性鼻-鼻窦炎的临床观察

Clinical Observation on Qingre Tongqiao Decoction Combined with Western Medicine in Treating Chronic Rhinosinusitis. *光明中医*, *34*(5), 781-783. https://doi.org/10.3969/j.issn.1003-8914.2019.05.052

钟艳萍, & 陈俊曦. (2007). 中药干预对慢性鼻窦炎鼻息肉术后疗效的影响. *中外健康文摘·医药月刊*, *4*(5), 24-25. https://d.wanfangdata.com.cn/periodical/ChlQZXJpb2RpY2FsQ0hJTmV3UzIwMjEwNjE2EhR6d2prd3oteXl5azIwMDcwNTAxORoIbHRtcm84YjY%3D

钟永晓. (2007). 辛荑苍耳子散治疗马属动物慢性鼻窦炎临床试验观察. *中兽医学杂志*(05), 10.

钟义均, & 王艳峰. (2021). 应用中医综合手段联合鼻内镜鼻窦微创手术治疗慢性鼻-鼻窦炎的临床疗效. *内蒙古中医药*, *40*(03), 110-111.

朱文憬, & 张玉萍. *中西医结合治疗慢性鼻窦炎鼻内窥镜术后47例* 第四届全国中西医结合耳鼻咽喉科学术会论文汇编, 贵阳. https://d.wanfangdata.com.cn/conference/ChZDb25mZXJlbmNlTmV3UzIwMjEwNTIxEgc2MDI2MjM2GghsdG1ybzhiNg%3D%3D

周振峰, 熊静, & 卢娅. (2013). 龙胆通窍丸治疗湿热型慢性鼻窦炎的临床观察

Clinical observation on treating chronic sinusitis with the Longdan Tongqiao pills. *中医临床研究*(16), 27-28. https://doi.org/10.3969/j.issn.1674-7860.2013.16.012

周振峰, 熊静, & 吴婷. (2015). 藿胆鼻渊汤治疗湿热型鼻窦炎的临床观察. *饮食保健*, *2*(10), 38. https://d.wanfangdata.com.cn/periodical/ChlQZXJpb2RpY2FsQ0hJTmV3UzIwMjEwNjE2Eg95aW5zYmoyMDE1MTAwNDIaCGNqOWo2djY3

朱晓朴. (2018). *醒窍汤在鼻窦炎围手术期的疗效观察及对MMP-2、7、9表达影响研究* [硕士, 南京中医药大学].

朱钦源, & 肖红英. (2018). 肺炎支原体感染性慢性鼻-鼻窦炎患者接受自拟中药鼻渊汤联合阿奇霉素肠溶片治疗的效果评价

The Efficacy of Biyuan Decoction Combined With Azithromycin Enteric-coated Tablets in With the Treatment of Mycoplasma Pneumoniae Infective Chronic Rhinosinusitis. *药品评价*, *15*(24), 55-57. https://doi.org/10.3969/j.issn.1672-2809.2018.24.017

仲崇玉, & 仲欣. (2020). 中药合剂治疗慢性鼻窦炎的疗效观察. *中国保健营养*, *30*(36), 338-339. https://d.wanfangdata.com.cn/periodical/ChlQZXJpb2RpY2FsQ0hJTmV3UzIwMjEwNjE2EhJ6Z2JqeXkta3AyMDIwMzYzMTcaCGNqOWo2djY3

陈可香, & 全梅玉. (2015). 中西医结合治疗慢性鼻窦炎疗效分析. *海峡药学*, *27*(10), 119-120. https://doi.org/10.3969/j.issn.1006-3765.2015.10.054

陈小宁, & 李金风. (2007). 清热化湿通窍汤在慢性鼻窦炎围手术期的应用体会. *中国中西医结合耳鼻咽喉科杂志*, *15*(4), 290-291,287. https://doi.org/10.3969/j.issn.1007-4856.2007.04.014

陈月婵. (2000). 海步香菊片治疗慢性化脓性上颌窦炎178例疗效观察

Effective Observation on 178 Cases Chronic Pyogenic Maxillary Sinusitis Treated by Hai Bu Xiang Ju Table. *贵阳中医学院学报*, *22*(2), 28. https://doi.org/10.3969/j.issn.1002-1108.2000.02.021

陈正辉. (2010). 健脾通窍汤在慢性鼻窦炎Ⅱ型围手术期的应用

Application of Jianpi Tongqiao decoction in type Ⅱ chronic sinusitis perioperative. *中医临床研究*, *2*(15), 70-71. https://doi.org/10.3969/j.issn.1674-7860.2010.15.043

陈璀璀, 李永磊, & 芦二永. (2018). 通窍鼻炎方对慢性鼻窦炎患者体内Foxa2、黏蛋白MUC5AC、HIF-1α水平的影响

Tongqiao Biyan recipe in Foxa2 patients with chronic sinusitis and MUC5AC protein levels of HIF-1 alpha change. *陕西中医*, *39*(8), 1105-1108. https://doi.org/10.3969/j.issn.1000-7369.2018.08.035

陈平. (2007). 中西医结合治疗急性鼻窦炎116例. *河南中医*, *27*(10), 58-59. https://doi.org/10.3969/j.issn.1003-5028.2007.10.043

陈平. (2010). 中西医结合治疗慢性鼻窦炎39例. *山东中医杂志*, *29*(8), 556-557. https://d.wanfangdata.com.cn/periodical/ChlQZXJpb2RpY2FsQ0hJTmV3UzIwMjEwNjE2Eg9zZHp5enoyMDEwMDgwMzAaCGNqOWo2djY3

蔡娥. (1997). 中西医结合治疗鼻窦炎140例. 世界中西医结合大会, 中国北京.

彭易坤, 王菊, 熊世珍, 漆一飞, 聂敏, 杨洋, 张良春, & 任娟娟. (2008). 香菊胶囊在慢性鼻-鼻窦炎鼻息肉内镜术后的临床应用

A clinical observation on the therapeutic effect of Xiangju capsules on chronic sinusitis and nasnal polyps following endoscopic sinus surgery. *中国实用医药*, *3*(17), 24-25. https://doi.org/10.3969/j.issn.1673-7555.2008.17.015

鲍玲. (2013). 中西药联合治疗儿童慢性鼻-鼻窦炎. *现代中西医结合杂志*, *22*(33), 3697-3699. https://doi.org/10.3969/j.issn.1008-8849.2013.33.019

何良如, 刘俊, 黄炳锋, & 陈伟. (2018). 鼻渊通窍颗粒联合曲安奈德鼻喷雾剂治疗慢性鼻-鼻窦炎的疗效观察

Clinical efficacy and safety of Biyuan Tongqiao granule combined with triamcinolone acetonide nasal spray in the treatment of chronic rhinosinusitis. *中国基层医药*, *25*(12), 1584-1588. https://doi.org/10.3760/cma.j.issn.1008-6706.2018.12.023

何顺芬, 刘铁陵, & 邓世明. (2013). 超声雾化配合中药内服治疗鼻渊78例疗效分析. *实用中医药杂志*, *29*(03), 161-162.

韩瑞华. (2018). 辨证分型联合西药治疗慢性鼻窦炎随机平行对照研究

Randomized Parallel Control Study on Syndrome Differentiation and Western Medicine in Treatment of Chronic Sinusitis. *实用中医内科杂志*, *32*(8), 48-51. https://doi.org/10.13729/j.issn.1671-7813.Z20180232

解礼杰, 张笑芳, & 甄甄. (2006). 慢性鼻窦炎中西药治疗对照观察76例

Effect of Traditional Medicine and Western medicine on Chronic nasosinusitis - a randomized controlled clinical trial. *中华实用中西医杂志*, *19*(2), 144-145. https://d.wanfangdata.com.cn/periodical/ChlQZXJpb2RpY2FsQ0hJTmV3UzIwMjEwNjE2EhJ6aHN5enh5enoyMDA2MDIwMTUaCGx0bXJvOGI2

叶谋华, & 张泳佳. (2014). 中西医结合治疗小儿慢性鼻-鼻窦炎30例效果分析. *当代医学*(22), 153-153. https://doi.org/10.3969/j.issn.1009-4393.2014.22.117

洪树鹏. (2007). 中西医结合治疗慢性鼻窦炎213例临床分析

A Clinical Analysis on the Therapeutic Effect of Integrative Medicine Therapy on Chronic Sinusitis Among 213 Cases. *中国医疗前沿*, *2*(18), 5,2. https://doi.org/10.3969/j.issn.1673-5552.2007.18.003

黄建, & 徐光宇. (2005). 自拟鼻窦炎汤治疗慢性鼻窦炎200例疗效观察. *国医论坛*, *20*(1), 31-32. https://doi.org/10.3969/j.issn.1002-1078.2005.01.025

黄桂锋. (2018). 黄芩汤合苍耳子散为主治疗慢性鼻窦炎的临床观察

Clinical Observation on Huangqin Decoction Combined with Fructus Xanthii Powder in the Treatment of Chronic Sinusitis. *光明中医*, *33*(14), 1998-2000. https://doi.org/10.3969/j.issn.1003-8914.2018.14.007

黄春江. (2014). 脾气虚弱型慢性鼻窦炎的临床诊治分析. *临床医药文献电子杂志*(13), 2436-2436. https://d.wanfangdata.com.cn/periodical/ChlQZXJpb2RpY2FsQ0hJTmV3UzIwMjEwNjE2EhFsY3l5ZHp6ejIwMTQxMzA5NBoIbHRtcm84YjY%3D

侯小兵, 夏晶晶, 王君, & 肖淑珍. (2017). 难治性鼻窦炎鼻内镜手术联合中药"苍耳子散"应用78例疗效观察. *中国中西医结合耳鼻咽喉科杂志*, *25*(2), 150-152. https://doi.org/10.16542/j.cnki.issn.1007-4856.2017.02.022

**2. without description of randomization method (n = 382)**

Ediriweera, E., Rathnayaka, R., Premakeerthi, W., & Weerasinghe, K. (2010). Efficacy of sri lankan traditional decoction of katuwelbatu deduru katukadi in treatment of kaphaja shira shula (chronic sinusitis). *Ayu*, *31*(1), 58‐61. https://www.cochranelibrary.com/central/doi/10.1002/central/CN-00856796/full

Jing, L., Chunquan, Z., Hai, L., Chen, Y., Siyuan, G., Yi, W., & Honggang, D. (2018). Effect of Zhu-yuan decoction in patients with chronic rhinosinusitis after functional endoscopic sinus surgery [Journal Article]. *Journal of Traditional Chinese Medicine*, *38*(1), 83-88. http://search.ebscohost.com/login.aspx?direct=true&db=amed&AN=5000552&site=ehost-live

强建华. (2011). 鼻渊合剂治疗慢性鼻-鼻窦炎30例临床观察

Clinical observation of Biyuan mixture on the treatment of chronic rhinosinusitis patients. *河北中医*, *33*(5), 668-670. https://doi.org/10.3969/j.issn.1002-2619.2011.05.012

江福山, & 马芳. (2015). 清热通窍汤联合西药治疗慢性鼻-鼻窦炎疗效观察

@@. *中国中医药科技*, *22*(5), 591-592. https://d.wanfangdata.com.cn/periodical/ChlQZXJpb2RpY2FsQ0hJTmV3UzIwMjEwNjE2EhB6Z3p5eWtqMjAxNTA1MDY5GghjZzR0MWxpMQ%3D%3D

郏雪峰. (2011). 内窥镜手术结合中药治疗鼻窦炎疗效分析. *中国医药科学*, *1*(17), 72.

高荣先, & 徐丽棉. (2014). 解毒法在治疗慢性鼻窦炎的临床应用效果分析. *医学信息*(15), 583-583. https://doi.org/10.3969/j.issn.1006-1959.2014.15.739

高志妹, & 陈磊. (2016). 通窍鼻炎方联合鼻腔冲洗治疗小儿急性鼻窦炎53例. *陕西中医*, *37*(09), 1219-1220.

高合增. (2016). 中西医结合治疗慢性化脓性鼻窦炎的方法探讨. *世界临床医学*, *10*(7), 166. https://d.wanfangdata.com.cn/periodical/ChlQZXJpb2RpY2FsQ0hJTmV3UzIwMjEwNjE2Eg9zamxjeXgyMDE2MDcxMzgaCGNqOWo2djY3

曲灵美, 刘峰, 金宏林, 于洋, & 徐光达. (2013). 中西医结合治疗儿童急性鼻窦炎的疗效观察. *中国中西医结合耳鼻咽喉科杂志*, *21*(2), 129-130. https://doi.org/10.3969/j.issn.1007-4856.2013.02.020

曲中源, & 孙海波. (2011). *慢性鼻窦炎鼻息肉鼻内镜术后应用菊花通圣汤临床观察* 世界中联耳鼻喉口腔专业委员会换届大会及第三次学术年会暨中华中医药学会耳鼻喉科分会第十七次学术交流会暨广东省中医及中西医结合学会耳鼻喉科学术交流会论文汇编, 中国广东广州. https://d.wanfangdata.com.cn/conference/ChZDb25mZXJlbmNlTmV3UzIwMjEwNTIxEg5IWTAwMDAwMzAxNTM3NxoING40dHd3M3Y%3D

龚明杰, & 邹嘉平. (2012). 辨证分型结合鼻内镜规范治疗慢性鼻窦炎鼻息肉60例. *陕西中医*, *33*(11), 1496-1497. https://doi.org/10.3969/j.issn.1000-7369.2012.11.040

孔婷婷, 王乐秋, 张爱华, & 付晴. (2017). 龙胆泻肝汤治疗胆腑郁热型慢性鼻-鼻窦炎的临床观察

Clinical Observation on Longdan Xiegan Decoction in Treatingof chronic rhinosinusitis. *中国保健营养*, *27*(31), 42-43. https://doi.org/10.3969/j.issn.1004-7484.2017.31.052

孔雋. (2014). 小儿鼻窦炎患者临床诊治分析. *吉林医学*(28), 6315-6315,6316. https://doi.org/10.3969/j.issn.1004-0412.2014.28.062

龚兴宏, 符晓, & 李用真. (2009). 中西医结合治疗慢性鼻窦炎62例临床观察. *现代中西医结合杂志*, *18*(2), 139-140. https://doi.org/10.3969/j.issn.1008-8849.2009.02.014

郭宏, 周永霞, 黄玲, 温映萍, & 寇丽霞. (2005). 鼻窦康颗粒治疗慢性鼻窦炎61例疗效观察. *新中医*, *37*(6), 22-23. https://doi.org/10.3969/j.issn.0256-7415.2005.06.009

郭国平. (2015). 鼻渊通窍颗粒治疗急性鼻窦炎的疗效观察. *临床合理用药杂志*, *8*(02), 89.

郭明刚. (2018). 曲安奈德鼻喷雾剂联合鼻渊通窍颗粒治疗慢性鼻-鼻窦炎临床观察. *实用中医药杂志*, *34*(10), 1233. https://doi.org/10.3969/j.issn.1004-2814.2018.10.076

郭靖. (2012). 连花清瘟胶囊治疗慢性鼻窦炎疗效观察. *中国社区医师(医学专业)*, *14*(4), 224-225. https://doi.org/10.3969/j.issn.1007-614x.2012.04.221

郭青, & 曹永贺. (2006). 鼻炎康汤治疗慢性鼻窦炎64例. *四川中医*, *24*(9), 86-87. https://doi.org/10.3969/j.issn.1000-3649.2006.09.059

郭泽举, & 王建平. (2011). 置换法并苍耳子散加味治疗儿童慢性鼻窦炎52例临床观察. *新中医*, *43*(1), 73-74. https://d.wanfangdata.com.cn/periodical/ChlQZXJpb2RpY2FsQ0hJTmV3UzIwMjEwNjE2Egx4enkyMDExMDEwMzYaCGNnNHQxbGkx

官佳意, & 陶波. (2016). 解郁祛湿建中汤治疗湿热型慢性鼻窦炎临床观察. *世界最新医学信息文摘（连续型电子期刊）*, *16*(68), 116-116,111. https://doi.org/10.3969/j.issn.1671-3141.2016.68.095

乔世安, 高俊安, & 朱优立. (2008). 中西医结合治疗急性鼻窦炎的临床观察. *中国民康医学（上半月）*, *20*(21), 2575. https://doi.org/10.3969/j.issn.1672-0369.2008.21.072

邱录斌, 杨见明, 梅金玉, & 朱永军. (2015). 疏风解毒胶囊治疗急性鼻窦炎55例疗效评价. *中国药业*, *24*(20), 58-60.

祁顺来, & 朵德龙. (2020). 中医辨证施治联合鼻喷糖皮质激素治疗慢性鼻—鼻窦炎伴鼻息肉的临床疗效. *中国保健营养*, *30*(25), 57. https://d.wanfangdata.com.cn/periodical/ChlQZXJpb2RpY2FsQ0hJTmV3UzIwMjEwNjE2EhJ6Z2JqeXkta3AyMDIwMjUwODMaCGNqOWo2djY3

金娜, 张桂杰, 刁建华, & 陈丽华. (2001). 中西医结合治疗慢性上颌窦炎168例

168 cases of chronic maxillitis treated by chinese medicine and foreign medicine. *中国疗养医学*, *10*(2), 18-19. https://doi.org/10.3969/j.issn.1005-619X.2001.02.010

覃冠锻, 彭清华, 莫炼, & 侯涛. (2011). 托里排脓法调治鼻窦炎术后患者的临床研究

Clinical observation on the therapeutic effect of treating method of Internal Expulsion Pus-Expelling on patients with chronic sinusitis following the treatment of endoscopic sinus surgery. *中国中西医结合耳鼻咽喉科杂志*, *19*(4), 252-254,259. https://doi.org/10.3969/j.issn.1007-4856.2011.04.010

唐成定, & 焦河玲. (2008). 辛芳通窍汤治疗慢性鼻窦炎40例. *河南中医*, *28*(10), 57-58. https://doi.org/10.3969/j.issn.1003-5028.2008.10.031

唐英, 李晓旭, & 柏杉. (2015). 加减甘露消毒冲剂配合鼻腔负压置换疗法治疗脾胃湿热型鼻渊. *现代中医药*, *35*(2), 25-27. https://doi.org/10.13424/j.cnki.mtcm.2015.02.012

戴润芝, & 马飞. (2015). 鼻渊舒口服液联合克拉霉素对慢性鼻窦炎患者症状及 Lund －kennedy 评分的影响

Effect of Symptoms and Lund - Kennedy Score on Treating Chronic Sinusitis Patients with Bi yuanshu Oral Liquid Combined C larithromycin Capsules. *湖北中医药大学学报*(3), 24-26. https://doi.org/10.3969/j.issn.1008-987x.2015.03.07

董韶昱, 李志刚, 胡少争, & 景朝丽. (2008). 中西医结合治疗儿童慢性鼻窦炎的临床观察

Clinical Research on Children's Chronic Rhinitis Treating with Combination of China Traditional Medicine and Western Medicine. *辽宁中医杂志*, *35*(12), 1889-1890. https://d.wanfangdata.com.cn/periodical/ChlQZXJpb2RpY2FsQ0hJTmV3UzIwMjEwNjE2Eg9sbnp5enoyMDA4MTIwNTQaCGNnNHQxbGkx

仝照全. (2015). 鼻内镜规范治疗配合中药内服对慢性鼻窦炎鼻息肉的临床疗效评价. *中国医药指南*(20), 115-115,116. https://d.wanfangdata.com.cn/periodical/ChlQZXJpb2RpY2FsQ0hJTmV3UzIwMjEwNjE2Eg96Z3l5em4yMDE1MjAwODYaCGx0bXJvOGI2

董洪飞. (2021). 自拟辛夷鼻炎汤治疗鼻渊的临床疗效. *特别健康*(16), 84. https://d.wanfangdata.com.cn/periodical/ChlQZXJpb2RpY2FsQ0hJTmV3UzIwMjEwNjE2Eg1qdGJqMjAyMTE2MDkxGghjajlqNnY2Nw%3D%3D

杜经纬, 冯俊, 彭涛, & 李志勇. (2016). 鼻窦炎口服液对 CRS 患者炎症因子及鼻腔 Lund-kennedy 评分的影响

The effects of sinusitis oral liquid on the inflammatory factors and nasal Lund-kennedy score in patients with CRS. *陕西中医*, *37*(8), 968-970. https://doi.org/10.3969/j.issn.1000-7369.2016.08.014

杜丽. (2006). 中西医结合治疗慢性鼻窦炎疗效观察

Therapeutic Observations on Treatment of Chronic Nasosinusitis by Combination of Chinese and Western Medicine. *吉林中医药*, *26*(2), 36-36. https://doi.org/10.3969/j.issn.1003-5699.2006.02.026

邓同民, & 李小艳. (2014). 慢性鼻-鼻窦炎围手术期应用通窍清窦汤的临床效果分析. *中国现代药物应用*, *8*(18), 164-165.

邓文娴, 刘元献, 翁镌凌, 蒋凡, & 黄廉鑫. (2019). 中医辨证施治联合鼻喷糖皮质激素治疗慢性鼻——鼻窦炎伴鼻息肉的临床观察. *广州中医药大学学报*, *36*(09), 1320-1325.

邓志峰. (2009). 自拟益气活血通窍汤联合阿奇霉素治疗慢性鼻窦炎48例疗效观察. *临床合理用药杂志*, *2*(02), 42.

邓清红. (2016). 中西医结合治疗慢性鼻－鼻窦炎疗效及对鼻纤毛传输功能的影响. *现代中西医结合杂志*(6), 626-628. https://doi.org/10.3969/j.issn.1008-8849.2016.06.019

罗辉, & 刘树春. (2008). 温阳通窍汤在慢性鼻窦炎Ⅱ型围手术期的应用. *中国中西医结合耳鼻咽喉科杂志*, *16*(5), 358-360. https://doi.org/10.3969/j.issn.1007-4856.2008.05.015

骆桂秋, 顾析玲, & 宋宪华. (2005). 苍耳子汤配合西药治疗小儿急性鼻窦炎73例--附单纯西药治疗62例对照. *浙江中医杂志*, *40*(7), 289. https://doi.org/10.3969/j.issn.0411-8421.2005.07.009

梁承志, & 黄彦. (2014a). “参苓白术散”的慢性鼻窦炎术后应用研究

"Three Atractylodes Ginseng Powder" in the Application Research on Chronic Sinusitis Surgery. *亚太传统医药*, *10*(8), 109-110. https://d.wanfangdata.com.cn/periodical/ChlQZXJpb2RpY2FsQ0hJTmV3UzIwMjEwNjE2Eg95dGN0eXkyMDE0MDgwNTkaCGNnNHQxbGkx

梁承志, & 黄彦. (2014b). 参苓白术散在慢性鼻窦炎术后的临床应用研究

"Three atractylodes ginseng powder" in the Application Research on Chronic Sinusitis Surgery. *亚太传统医药*, *10*(9), 102-104. https://d.wanfangdata.com.cn/periodical/ChlQZXJpb2RpY2FsQ0hJTmV3UzIwMjEwNjE2Eg95dGN0eXkyMDE0MDkwNTQaCGNnNHQxbGkx

梁云燕. (2012). 参苓白术散加减方对Ⅱ型鼻窦炎术后促进黏膜上皮化疗效观察. *新中医*, *44*(12), 75-76.

黎英锐. (2013). 中西医结合治疗小儿慢性鼻窦炎的疗效观察

Observation of integrated traditional Chinese and western medicine in the treatment of children with chronic sinusitis. *中国临床新医学*, *6*(2), 136-138. https://doi.org/10.3969/j.issn.1674-3806.2013.02.14

吕唯. (2013). 中西医结合治疗鼻窦炎疗效观察. *内蒙古中医药*, *32*(03), 81-82.

廖礼兵, 甘忠, & 吴仕九. (2007). 清香散加味治疗湿热型鼻窦炎60例. *中国中医药科技*, *14*(3), 封4. https://doi.org/10.3969/j.issn.1005-7072.2007.03.070

廖伟, & 郭新铭. (2011). 鼻渊通窍颗粒治疗慢性鼻窦炎的临床评价. *中国老年保健医学*, *9*(3), 36-38. https://doi.org/10.3969/j.issn.1672-4860-B.2011.03.016

龙益兴, 陈豪, & 朱能. (2015). 鼻渊汤配合西药治疗儿童慢性鼻窦炎疗效观察. *新中医*, *47*(03), 187-188.

刘建强. (2016). 鼻渊通窍颗粒联合阿莫西林治疗急性鼻窦炎49例

Biyuan Tongqiao Granule combined with Amoxicillin in the Treatment of Acute Sinusitis for 49 Cases. *中国中医药现代远程教育*, *14*(1), 86-87. https://doi.org/10.3969/j.issn.1672-2779.2016.01.045

刘国磊, 李春义, & 陈淼. (2018). 鼻炎康汤治疗慢性鼻-鼻窦炎胆腑郁热证疗效及对鼻黏膜纤毛传输速率的影响. *现代中西医结合杂志*, *27*(18), 1984-1986.

刘乃斌, 尹春雷, 宗涛, & 徐建华. (2019). 龙胆泻肝汤联合西药治疗慢性鼻-鼻窦炎不伴鼻息肉的临床探讨. *健康之友*(17), 19. https://d.wanfangdata.com.cn/periodical/ChlQZXJpb2RpY2FsQ0hJTmV3UzIwMjEwNjE2Eg1qa3p5MjAxOTE3MDI2GghjZzR0MWxpMQ%3D%3D

刘丽媛. (2015). 中西医结合治疗小儿慢性鼻-鼻窦炎. *心理医生*(3), 106-106. https://d.wanfangdata.com.cn/periodical/ChlQZXJpb2RpY2FsQ0hJTmV3UzIwMjEwNjE2Eg94bHlzLXgyMDE1MDMwNzAaCGNnNHQxbGkx

刘万忠. (2015). 香菊胶囊在慢性鼻-鼻窦炎患者鼻内镜术后的应用效果. *亚太传统医药*, *11*(17), 127-128.

刘彬. (2019). 辛芳鼻炎胶囊联合罗红霉素治疗慢性鼻窦炎的临床价值探讨. *健康必读*(25), 77. https://d.wanfangdata.com.cn/periodical/ChlQZXJpb2RpY2FsQ0hJTmV3UzIwMjEwNjE2EhBqa2JkMDAxMjAxOTI1MTIwGghjajlqNnY2Nw%3D%3D

刘小云. (2013). 中西医结合治疗鼻窦炎100例. *中国中医药现代远程教育*, *11*(18), 83-83. https://doi.org/10.3969/j.issn.1672-2779.2013.18.064

刘毅, & 戴筱杰. (2016). 鼻渊软胶囊联合布地奈德鼻喷雾剂治疗慢性鼻窦炎的效果探析

Discussion on the Effect of Biyuan Soft Capsule combined with Budesonide Nasal Spray in the Treatment of Chronic Sinusitis. *中国中医药现代远程教育*, *14*(12), 100-102. https://doi.org/10.3969/j.issn.1672-2779.2016.12.043

刘仁辉. (2011). 鼻窦炎鼻内窥镜围手术期的中西医结合治疗临床分析

Endoscopic sinus surgery around the Integrative Medicine. *中国中医药咨讯*, *3*(18), 139-140. https://d.wanfangdata.com.cn/periodical/ChlQZXJpb2RpY2FsQ0hJTmV3UzIwMjEwNjE2EhB6Z3p5eXp4MjAxMTE4MDkzGghjZzR0MWxpMQ%3D%3D

刘全. (2013). 中西医结合治疗儿童慢性鼻窦炎临床疗效观察

Chinical observation on the therapeutic effect of integrative medicine therapy onchronic sinusitis in children. *中国保健营养（中旬刊）*(8), 471-471. https://d.wanfangdata.com.cn/periodical/ChlQZXJpb2RpY2FsQ0hJTmV3UzIwMjEwNjE2EhJ6Z2JqeXktejIwMTMwODA3MDkaCGNqOWo2djY3

刘晶. (2013). 鼻渊通窍颗粒治疗儿童慢性鼻窦炎疗效观察. *临床医药实践*, *22*(11), 833-834. https://d.wanfangdata.com.cn/periodical/ChlQZXJpb2RpY2FsQ0hJTmV3UzIwMjEwNjE2Eg9zeGxjeXkyMDEzMTEwMTUaCGNqOWo2djY3

刘静, 唐艺芬, & 朱佳. (2016). 鼻渊舒口服液合西医常规治疗儿童慢性鼻窦炎53例临床观察. *中医药导报*, *22*(05), 89-91.

刘增, 孔祥春, & 潘尚战. (2019). 苍耳子鼻炎胶囊联合克拉霉素治疗慢性鼻窦炎的临床研究

Clinical study on Cangerzi Biyan Capsules combined with clarithromycin in treatment of chronic sinusitis. *现代药物与临床*, *34*(3), 798-801. https://doi.org/10.7501/j.issn.1674-5515.2019.03.048

刘倩, 周舟, & 叶少清. (2011). 常规+鼻渊通窍治疗慢性鼻窦炎伴鼻息肉术后的疗效分析

Curative effect of chronic sinusitis with nasal polyps after endoscopic sinus surgery by Conventional + Biyuan Tongqiao treatment. *中外健康文摘*, *8*(19), 45-46. https://doi.org/10.3969/j.issn.1672-5085.2011.19.032

刘春松, 邱宝珊, & 王士贞. (2009). 功能性鼻窦内窥镜手术后应用中药辨证治疗的临床观察. *辽宁中医药大学学报*, *11*(7), 95-96. https://d.wanfangdata.com.cn/periodical/ChlQZXJpb2RpY2FsQ0hJTmV3UzIwMjEwNjE2EhFsbnp5eHl4YjIwMDkwNzA1MxoIbHRtcm84YjY%3D

刘宪宾. (2017). 不同中医辨证分型慢性鼻-鼻窦炎患者鼻内窥镜术后中医药治疗的临床分析. 国际数字医学会数字中医药分会年会暨第二届数字中医药学术交流会, 中国广东广州.

刘现云. (2011). 窦舒汤治疗湿热型慢性鼻窦炎63例疗效观察. *河北中医*, *33*(12), 1793-1794. https://doi.org/10.3969/j.issn.1002-2619.2011.12.012

刘洪珍. (2002). 中西药治疗慢性上颌窦炎的效果评价. *职业与健康*, *18*(1), 102-103. https://doi.org/10.3969/j.issn.1004-1257.2002.01.093

李庆国. (2017). 探讨中西医治疗肺炎支原体感染性慢性鼻-鼻窦炎的临床疗效. *养生保健指南*(34), 48. https://doi.org/10.3969/j.issn.1006-6845.2017.34.043

李果丽, 符晓, 龚兴宏, & 陈志勇. (2018). 益气通窍汤治疗肺脾气虚型儿童慢性鼻窦炎70例. *中国民族民间医药*, *27*(16), 98-100. https://d.wanfangdata.com.cn/periodical/ChlQZXJpb2RpY2FsQ0hJTmV3UzIwMjEwNjE2EhN6Z216bWp5eXp6MjAxODE2MDM1GghjZzR0MWxpMQ%3D%3D

李巧凤. (2004). 苍耳子系列方治疗儿童及青少年慢性鼻窦炎106例--附单纯西药治疗62例对照. *浙江中医杂志*, *39*(8), 338. https://doi.org/10.3969/j.issn.0411-8421.2004.08.011

李娜, 梁巍, 张楠, 徐鑫, 朱晶, & 史霁. (2016). 分析中西医结合治疗小儿慢性鼻窦炎的临床效果. *中国保健营养*, *26*(30), 369-370. https://doi.org/10.3969/j.issn.1004-7484.2016.30.607

李娜, 朱晶, 张楠, 梁巍, 徐鑫, & 杨雪. (2016). 补虚托毒通窍汤治疗小儿慢性鼻窦炎的临床疗效分析. *中国保健营养*, *26*(33), 380-380,381. https://doi.org/10.3969/j.issn.1004-7484.2016.33.631

李丹青, 路广余, 张亚琴, 张琪, 钟旻轶, 苏俊, & SHEN, H. (2013). 头孢丙烯与玉屏风散联合治疗急性细菌感染性鼻窦炎的临床研究. *中华医院感染学杂志*, *23*(24), 6106-6108.

李玲珑. (2017). *鼻渊舒口服液抑制慢性鼻—鼻窦炎表皮葡萄球菌细菌生物膜调控基因luxS、icaA表达的研究* [硕士, 成都中医药大学].

李美爱. (2009). 健脾化浊通窍汤治疗鼻窦炎术后流粘涕的体会. *中外医疗*, *28*(32), 95. https://doi.org/10.3969/j.issn.1674-0742.2009.32.078

李博聿. (2015). 薏苡附子败酱散合千金苇茎汤加桔梗治疗慢性鼻窦炎54例. *内蒙古中医药*, *34*(1), 31-32. https://doi.org/10.3969/j.issn.1006-0979.2015.01.030

李雪梅. (2018). 中医护理干预对慢性鼻窦炎围手术期的效果分析. *养生保健指南*(8), 203. https://doi.org/10.3969/j.issn.1006-6845.2018.08.187

李少华. (2008). *慢性鼻窦炎内镜术后中医综合治疗及证型转归的初步研究* [硕士, 广州中医药大学]. https://d.wanfangdata.com.cn/thesis/ChJUaGVzaXNOZXdTMjAyMTA1MTkSCUQwMTMyMjQ0MhoIbHRtcm84YjY%3D

李秀娜. (2014). *珍芪汤对慢性鼻-鼻窦炎术后恢复影响的临床观察* [硕士, 成都中医药大学]. https://d.wanfangdata.com.cn/thesis/ChJUaGVzaXNOZXdTMjAyMTA1MTkSB0Q3MDg4MDMaCGNnNHQxbGkx

李树广, & 马丽华. (2003). 鼻窦炎口服液的临床应用与疗效观察. *中国煤炭工业医学杂志*, *6*(6), 573-574. https://doi.org/10.3969/j.issn.1007-9564.2003.06.081

李深良. (2014). 升麻解毒汤治疗慢性鼻窦炎25例. *中国中医药现代远程教育*, *12*(14), 49-50. https://doi.org/10.3969/j.issn.1672-2779.2014.14.028

李安平. (2019). 鼻渊丸对慢性鼻-鼻窦炎术后鼻黏膜恢复的效果观察. *中国现代药物应用*, *13*(05), 141-142.

李爱贵. (2019). 中西医结合治疗慢性鼻窦炎的临床效果观察. *世界临床医学*, *13*(4), 197. https://d.wanfangdata.com.cn/periodical/ChlQZXJpb2RpY2FsQ0hJTmV3UzIwMjEwNjE2Eg9zamxjeXgyMDE5MDQxNTMaCGNqOWo2djY3

李延清. (2011). 小儿鼻炎鼻窦炎的治疗体会. *吉林医学*, *32*(27), 5644.

李玉翠. (2020). 小儿鼻炎鼻窦炎的保守治疗效果观察. *母婴世界*(23), 80. https://d.wanfangdata.com.cn/periodical/ChlQZXJpb2RpY2FsQ0hJTmV3UzIwMjEwNjE2Eg1teXNqMjAyMDIzMDc1GghjajlqNnY2Nw%3D%3D

李友勋. (2001). 鼻康丸的制配及疗效观察. *海峡药学*(04), 19-20.

李韵霞, 杨志刚, & 李浩. (2014). 中西医结合治疗鼻息肉术后患者的临床观察

@@. *中国中西医结合耳鼻咽喉科杂志*, *22*(5), 379-381,375. https://doi.org/10.3969/j.issn.1007-4856.2014.05.021

李伟, 徐源, & 刘海燕. (2003). 慢性鼻窦炎鼻息肉鼻内镜手术后应用中药治疗的临床观察

Clinical observation on the therapeutic effect of integrated therapy with ESS and SOL following the operation on chronic Sinusitis. *中国中西医结合耳鼻咽喉科杂志*, *11*(5), 228-230. https://doi.org/10.3969/j.issn.1007-4856.2003.05.006

李银仓. (2008). 中西医结合治疗儿童慢性鼻窦炎疗效观察

The Curative Effect of the Combination of Traditional Chinese and Western Medicine on Chronic Sinusitis in Children. *中国现代医生*, *46*(22), 36-37. https://doi.org/10.3969/j.issn.1673-9701.2008.22.017

李静, 郑春泉, 林海, 杨晨, & 汪毅. (2015). *"逐渊汤"用于鼻窦炎FESS术后的疗效观察* 2015年全国鼻科年会暨第七届鼻部感染与变态反应疾病专题学术会议论文集, 天津. https://d.wanfangdata.com.cn/conference/ChZDb25mZXJlbmNlTmV3UzIwMjEwNTIxEgc4NzU2ODExGghjajlqNnY2Nw%3D%3D

李俊秀. (2016). 克拉霉素联合香菊胶囊治疗慢性鼻窦炎的疗效及安全性观察. *世界最新医学信息文摘*(16), 143+142.

李中原. (2013). 中西医结合治疗慢性鼻窦炎68例. *中国中医药现代远程教育*, *11*(14), 39-40. https://doi.org/10.3969/j.issn.1672-2779.2013.14.028

李春杰. (2016). 鼻窦内窥镜手术联合辨证施药对慢性鼻窦炎临床观察

@@. *四川中医*, *34*(4), 176-178. https://d.wanfangdata.com.cn/periodical/ChlQZXJpb2RpY2FsQ0hJTmV3UzIwMjEwNjE2Eg1zY3p5MjAxNjA0MDc3Ggg0bjR0d3czdg%3D%3D

李平. (2013). 香菊胶囊治疗慢性鼻-鼻窦炎的临床观察. *中国现代药物应用*, *7*(21), 167-168. https://d.wanfangdata.com.cn/periodical/ChlQZXJpb2RpY2FsQ0hJTmV3UzIwMjEwNjE2EhF6Z3hkeXl5eTIwMTMyMTE0NRoIbHRtcm84YjY%3D

李霞, 李炜, & 郑华平. (2010). 鼻内镜术合中西药治疗慢性鼻窦炎60例临床观察

Clinical Observation on Treatment of 60 Cases of Chronic Sinusitis with Chinese and Western Medicine. *江苏中医药*, *42*(8), 34-35. https://doi.org/10.3969/j.issn.1672-397X.2010.08.022

李许娜. (2016). 慢性鼻窦炎患者内镜术后中医辨证治疗的疗效分析. *中医临床研究*, *8*(12), 75-76.

利显民. (2001). 鼻窦炎口服液治疗慢性鼻窦炎117例临床观察. *广东医学院学报*, *19*(3), 208-209. https://doi.org/10.3969/j.issn.1005-4057.2001.03.025

李慧, 韩跃峰, 张明洁, 王晓敏, 陈德尚, & 马士崟. (2018). 藿胆片在慢性鼻-鼻窦炎围手术期治疗的应用研究

The applied research of Huodan tablet in chronic nasal-sinusitis perioperative treatment. *中国中西医结合耳鼻咽喉科杂志*, *26*(6), 440-443,447. https://doi.org/10.16542/j.cnki.issn.1007-4856.2018.06.010

李红. (2014). 中医鼻渊方治疗鼻窦炎100例临床观察. *中医临床研究*, *6*(21), 76-77.

李红. (2020). 布地奈德鼻喷雾剂联合鼻渊通窍颗粒对老年慢性鼻-鼻窦炎患者术后康复的影响观察. *首都食品与医药*, *27*(12), 86. https://d.wanfangdata.com.cn/periodical/ChlQZXJpb2RpY2FsQ0hJTmV3UzIwMjEwNjE2Eg1zZHl5MjAyMDEyMDc4GghjajlqNnY2Nw%3D%3D

李洪锋. (2020). 鼻内镜手术结合葛根汤加减治疗难治性鼻窦炎临床分析. *实用中医药杂志*, *36*(3), 272-273. https://d.wanfangdata.com.cn/periodical/ChlQZXJpb2RpY2FsQ0hJTmV3UzIwMjEwNjE2EhBzeXp5eXp6MjAyMDAzMDAzGghjajlqNnY2Nw%3D%3D

李辉. (2015). 鼻渊汤治疗慢性鼻窦炎疗效观察

Nasosinusitis Relieving Decoction treatment of chronic sinusitis curative effect observation. *内蒙古中医药*(7), 36-37. https://doi.org/10.3969/j.issn.1006-0979.2015.07.038

蔺林, 戴飞, & 程雷. (2015). 连花清瘟颗粒对成人非复杂性细菌性急性鼻-鼻窦炎的治疗作用

The treatment of Lianhuaqing wen for uncomplicated acute bacterial rhinosinusitis. *中国中西医结合耳鼻咽喉科杂志*, *23*(6), 414-419,431. https://doi.org/10.16542/j.cnki.issn.1007-4856.2015.06.004

林君. (2014). 鼻渊通窍颗粒联合克拉霉素颗粒治疗小儿慢性鼻窦炎的疗效观察

The effect of Biyuan Tongqiao granule combined clarithromycin granules on children with chronic sinusitis. *中国现代医生*, *52*(32), 34-36. https://d.wanfangdata.com.cn/periodical/ChlQZXJpb2RpY2FsQ0hJTmV3UzIwMjEwNjE2EhZ6d2tqemxtbC15eXdzMjAxNDMyMDExGghjajlqNnY2Nw%3D%3D

林丹娜. (2007). 中西医结合治疗慢性鼻窦炎急性发作50例观察. *实用中医药杂志*(03), 168.

林玲玲. (2006). *慢性鼻窦炎内窥镜鼻窦手术术后中医辨证治疗的疗效观察* [硕士, 广州中医药大学].

林玲玲, 林少雄, 程耿斌, & 蔡晓敏. (2016a). 不同中医辨证分型慢性鼻-鼻窦炎患者鼻内窥镜术后中医药治疗的临床研究

Clinical Study on Traditional Chinese Medicine of Syndrome Differentiation and Treatment on Chronic Nasal Sinusitis after Endoscopic Sinus Surgery. *中国医学创新*, *13*(17), 101-103. https://doi.org/10.3969/j.issn.1674-4985.2016.17.028

林玲玲, 林少雄, 程耿斌, & 蔡晓敏. (2016b). 中医辨证治疗对慢性鼻－鼻窦炎术后生活质量影响

Influence of TCM Syndrome Differentiation on the Quality of Life of Patients with Chronic Nasal Sinusitis. *光明中医*, *31*(19), 2768-2770. https://doi.org/10.3969/j.issn.1003-8914.2016.19.005

林玲玲, 林少雄, 程耿斌, & 蔡晓敏. (2016c). 中医药辨证治疗慢性鼻-鼻窦炎患者术后远期疗效评价

The evaluation for the long -term efficacy of TCM differential method in the treatment of chronic rhinosinusitis after surgery. *现代诊断与治疗*, *27*(18), 3333-3334,3335. https://d.wanfangdata.com.cn/periodical/ChlQZXJpb2RpY2FsQ0hJTmV3UzIwMjEwNjE2EhB4ZHpkeXpsMjAxNjE4MDAxGghjajlqNnY2Nw%3D%3D

林伦清, & 陈翀. (2009). 中西医结合治疗慢性鼻-鼻窦炎86例临床观察

The Clinical Observation of 86 Patients Suffering from Chronic Rhinitis or Sinusitis but Treated with Chinese-Western Medical Treatment. *中国现代医生*, *47*(32), 142-143. https://doi.org/10.3969/j.issn.1673-9701.2009.32.078

林驰. (2014). 苍耳子鼻炎滴丸治疗慢性鼻窦炎的临床疗效观察

@@. *四川中医*, *32*(10), 170-172. https://d.wanfangdata.com.cn/periodical/ChlQZXJpb2RpY2FsQ0hJTmV3UzIwMjEwNjE2Eg1zY3p5MjAxNDEwMDc2GghsdG1ybzhiNg%3D%3D

马庆华, & 郭守明. (2011). 香菊胶囊治疗儿童慢性鼻窦炎疗效观察

Xiangju capsule for children with chronic sinusitis clinical observation. *中国医学创新*, *8*(13), 47-48. https://doi.org/10.3969/j.issn.1674-4985.2011.13.026

马勤贞. (2010). 中西医结合治疗慢性鼻窦炎急性发作25例疗效观察. *中国社区医师(医学专业)*, *12*(4), 93. https://doi.org/10.3969/j.issn.1007-614x.2010.04.123

马生莲. (2010). 中西医结合治疗小儿急性鼻窦炎52例疗效观察. *中国中医药科技*, *17*(2), 105. https://doi.org/10.3969/j.issn.1005-7072.2010.02.065

马成, 刘炜明, 杨光远, & 王永国. (2018). 验方鼻窦炎丸治疗慢性鼻窦炎的临床研究

Clinical study of prescription sinusitis pills in the treatment of chronic nasosinusitis. *中国社区医师*, *34*(5), 89-90. https://doi.org/10.3969/j.issn.1007-614x.2018.5.53

马世明. (2017). 苍耳子散加减治疗难治性慢性鼻窦炎169例疗效观察. *内蒙古医学杂志*, *49*(6), 690-691. https://doi.org/10.16096/J.cnki.nmgyxzz.2017.49.06.018

马少民, 马海艳, 梁续伟, 孟宇青, & 栾玉. (2019). 布地奈德鼻喷雾剂联合鼻渊通窍颗粒治疗 儿童慢性鼻-鼻窦炎的效果

Clinical Study on Budesonide Nasal Spray Combined with Biyuan Tongqiao Granule in the Treatment of Chronic Rhinosinusitis, Upper Respiratory and Orbital Complications in Children. *宁夏医科大学学报*, *41*(5), 522-526. https://doi.org/10.16050/j.cnki.issn1674-6309.2019.04.021

马荧雪. (2017). 鼻渊舒联合头孢地尼分散片治疗小儿慢性鼻窦炎的疗效刍议

Clinical Analysis of Nasosinusitis Relieving Oral Liquid Combined with Cef-dinir Dispersible Tablets on Children with Chronic Sinusitis. *中外医疗*, *36*(29), 106-108. https://doi.org/10.16662/j.cnki.1674-0742.2017.29.106

马晓军. (2016). 甘露消毒丹加减配合鼻腔负压置换疗法治疗脾胃湿热型鼻渊的疗效评价. *陕西中医*, *37*(8), 980-981. https://doi.org/10.3969/j.issn.1000-7369.2016.08.019

莫凌凌. (2012). 香菊胶囊治疗慢性鼻窦炎60例的疗效观察

Clinical observation on treating 60 cases of chronic sinusitis with Xiangju capsule. *中医临床研究*, *4*(6), 82-83. https://doi.org/10.3969/j.issn.1674-7860.2012.06.051

万吴, & 汉徽. (2015). *香菊胶囊联合莫西沙星治疗急性鼻窦炎患者的临床疗效和安全性分析* 2015年全国鼻科年会暨第七届鼻部感染与变态反应疾病专题学术会议论文集, 天津. https://d.wanfangdata.com.cn/conference/ChZDb25mZXJlbmNlTmV3UzIwMjEwNTIxEgc4NzU2NzYxGghsdG1ybzhiNg%3D%3D

孟亚军. (2014). 鼻渊舒口服液预防鼻腔术后粘连疗效观察. *陕西中医*(10), 1347-1347,1348. https://doi.org/10.3969/j.issn.1000-7369.2014.10.038

孟亚军. (2020). 参苓白术散合苍耳子散治疗慢性化脓性鼻窦炎疗效观察. *养生保健指南*(29), 249. https://d.wanfangdata.com.cn/periodical/ChlQZXJpb2RpY2FsQ0hJTmV3UzIwMjEwNjE2EhF5c2Jqem4teDIwMjAyOTI0NhoIY2o5ajZ2Njc%3D

毛承深, & 胡娟. (2001). 中西医结合治疗慢性鼻窦炎178例疗效观察. *中国中西医结合杂志*, *21*(9), 712-713. https://d.wanfangdata.com.cn/periodical/ChlQZXJpb2RpY2FsQ0hJTmV3UzIwMjEwNjE2Eg56eHlqaDIwMDEwOTAyORoIY2c0dDFsaTE%3D

毛承深, 胡娟, 刘盛林, & 孙阳. (2002). 苍辛汤对鼻内窥镜手术后疗效的影响

A clinical observation on the influence of administered LXFMB on the post-operative thera-peutic effect of ESS. *中国中西医结合耳鼻咽喉科杂志*, *10*(5), 219-221. https://doi.org/10.3969/j.issn.1007-4856.2002.05.005

毛新龙. (2007). 自拟鼻渊宁口服液治疗慢性鼻窦炎55例临床观察. *海南医学*, *18*(12), 141. https://doi.org/10.3969/j.issn.1003-6350.2007.12.093

毛贺娟, 蒋蓉, 赵倩, 颜光堂, 程奇, 王建洪, & 肖世强. (2014). 驱风舒渊汤联合罗红霉素治疗青少年慢性鼻窦炎37例临床观察

The Clinical Efficacy of Combining Qufeng Shuyuan Decoction with Roxithromycin on Adolescents with Chronic sinusitis: A Clinical Observation of 37 Cases. *中医药导报*, *20*(8), 40-41,45. https://d.wanfangdata.com.cn/periodical/ChlQZXJpb2RpY2FsQ0hJTmV3UzIwMjEwNjE2EhBobnp5eWRiMjAxNDA4MDEzGghjajlqNnY2Nw%3D%3D

蒙成星. (2013). 鼻窦炎鼻息肉术后68例临床疗效分析. *世界最新医学信息文摘（电子版）*(4), 422-422,419. https://doi.org/10.3969/j.issn.1671-3141.2013.04.321

文景爱, 陈宏, 高照渝, & 丛军兹. (2008). 中医辨证配合西医常规护理对慢性鼻窦炎鼻内镜手术疗效的影响

The Influence of Integrated Traditional Chinese and Western Medical Nursing on the Therapeutic Effects of Chronic Sinusitis with Nasal Endoscopic Surgery. *国际中医中药杂志*, *30*(6), 478-479. https://doi.org/10.3760/cma.j.issn.1673-4246.2008.06.041

潘文剑. (2016). 香菊胶囊治疗儿童慢性鼻窦炎的临床疗效观察. *世界最新医学信息文摘*, *16*(78), 110.

潘应霞. (2012). 小儿鼻炎鼻窦炎保守治疗临床分析. *中国卫生产业*, *9*(17), 108.

方玲, & 方向. (2014). 中西医结合治疗鼻窦炎65例

@@. *河南中医*, *34*(9), 1821-1822. https://d.wanfangdata.com.cn/periodical/ChlQZXJpb2RpY2FsQ0hJTmV3UzIwMjEwNjE2Eg1obnp5MjAxNDA5MDkxGghjZzR0MWxpMQ%3D%3D

柏雪坤, & 殷华荣. (2003). 加味玉屏风散治疗慢性副鼻窦炎的疗效观察. *现代中西医结合杂志*, *12*(4), 365-365. https://doi.org/10.3969/j.issn.1008-8849.2003.04.018

樊玉敏. (2005). 鼻渊通消丸治疗青少年儿童慢性鼻窦炎临床观察. *中国中西医结合耳鼻咽喉科杂志*, *13*(6), 349. https://doi.org/10.3969/j.issn.1007-4856.2005.06.020

樊治军. (2013). 内窥镜手术结合中药治疗鼻窦炎疗效分析

Analysis of the Therapeutic Effectiveness of Treating Nasosinusitis by Endoscopy Operation Combined with Chinese Medicine. *辽宁医学院学报*, *34*(5), 21-23. https://doi.org/10.3969/j.issn.1674-0424.2013.05.008

樊治军. (2014). 中西医结合治疗变应性真菌性鼻窦炎临床观察

Clinical Observation of Integrated Chinese-western Therapy on Allergic Fungal Sinusitis. *湖北中医药大学学报*, *16*(3), 88-89. https://doi.org/10.3969/j.issn.1008-987x.2014.03.31

范成平. (2018). 通窍固表汤配合鼻内镜手术治疗慢性鼻窦炎的临床分析

Clinical Analysis of Tongqiao Gubiao Decoction Combined with Endoscopic Sinus Surgery for Chronic Sinusitis. *智慧健康*, *4*(5), 54-55,65. https://doi.org/10.19335/j.cnki.2096-1219.2018.05.024

范绍翀, 潘永, 叶浩昕, 钟晓声, 黄桢, & 黄莉美. (2012). 鼻渊通窍颗粒治疗儿童慢性鼻窦炎的疗效观察. *中药材*, *35*(5), 841-842. https://d.wanfangdata.com.cn/periodical/ChlQZXJpb2RpY2FsQ0hJTmV3UzIwMjEwNjE2Egx6eWMyMDEyMDUwNDcaCGNqOWo2djY3

宝全, & 那仁满都拉. (2013). 蒙药尼达哈召日丸治疗慢性鼻窦炎临床观察. *中国民族医药杂志*, *19*(12), 5-6.

付高尚, 徐艳霞, 魏艳艳, 许莹, & 韩富根. (2020). 通窍鼻炎颗粒联合头孢地尼对慢性鼻窦炎患儿症状改善及炎症指标的影响. *内蒙古医学杂志*, *52*(08), 955-957.

付选香, & 陈建平. (2015). 鼻渊通窍颗粒治疗内窥镜下鼻窦手术后并发症临床研究

Clinical Study on Application of Biyuan Tongqiao Granules After Endoscopic Sinus Surgery. *河南中医*, *35*(10), 2550-2551. https://doi.org/10.16367/j.issn.1003-5028.2015.10.1092

傅时伟. (2015). 鼻内镜手术联合霍胆丸治疗慢性化脓性鼻窦炎疗效观察

@@. *现代中西医结合杂志*, *24*(12), 1313-1315. https://doi.org/10.3969/j.issn.1008-8849.2015.12.022

付云朝. (2004). 中西医结合治疗小儿鼻窦炎134例疗效观察. *现代中西医结合杂志*, *13*(5), 589-590. https://doi.org/10.3969/j.issn.1008-8849.2004.05.020

傅云朝. (2011). 慢性鼻窦炎的中西医结合治疗观察. *实用心脑肺血管病杂志*, *19*(3), 458-459. https://doi.org/10.3969/j.issn.1008-5971.2011.03.080

史凤珍, & 董调红. (2016). 中西医结合治疗慢性鼻窦炎疗效观察. *山西中医*, *32*(02), 33+37.

史振铎. (2005). 自拟清窦散治疗副鼻窦炎临床观察 --附 1500例资料分析. *中国中医急症*, *14*(5), 437-438. https://doi.org/10.3969/j.issn.1004-745X.2005.05.025

史春和. (2011). 中西医结合治疗慢性鼻窦炎116例临床观察. *江苏中医药*, *43*(9), 53-54. https://doi.org/10.3969/j.issn.1672-397X.2011.09.035

沙海滨. (2009). 鼻内窥镜鼻窦手术配合中药治疗慢性鼻窦炎鼻息肉

Clinical Observation on Treatment of 65 Cases of Chronic Sinusitis and Nasal Polyps with Endoscopic Sinus Surgery Combined with Chinese Medicine. *国际中医中药杂志*, *31*(4), 337. https://doi.org/10.3760/cma.j.issn.1673-4246.2009.04.027

谢洁. (2007). 黄芪菖蒲泽泻汤治疗慢性鼻窦炎86例. *陕西中医*, *28*(12), 1633-1634. https://doi.org/10.3969/j.issn.1000-7369.2007.12.038

谢兴笔, & 刘文军. (2015). 清鼻丸对鼻窦炎鼻内窥镜术后黏膜的影响研究

Effect of Qingbi Pills on Mucosa of Patients with Sinusitis after Endoscopic Sinus Surgery. *河南中医*, *35*(7), 1692-1694. https://doi.org/10.16367/j.issn.1003-5028.2015.07.0716

徐可慰. (2013). 清鼻丸治疗急性鼻窦炎的临床疗效. *求医问药(下半月)*, *11*(11), 151.

徐开睿, & 黄庆琳. (2015). 中药配合鼻内镜手术治疗鼻窦炎鼻息肉疗效观察. *新中医*, *47*(02), 131-132.

徐庆文, 孙一帆, 凌娅娅, 卢标清, 周小军, 彭宏彬, & 戴滨泉. (2005). 中西医结合治疗慢性鼻窦炎92例疗效观察. *新中医*, *37*(11), 59-60. https://doi.org/10.3969/j.issn.0256-7415.2005.11.028

徐巧. (2015). 中西医结合在儿童急性鼻窦炎治疗中的疗效研究. *母婴世界*(6), 68-69. https://d.wanfangdata.com.cn/periodical/ChlQZXJpb2RpY2FsQ0hJTmV3UzIwMjEwNjE2Eg1teXNqMjAxNTA2MDY4GghjajlqNnY2Nw%3D%3D

徐丽娜, & 孙海波. (2020). 补气助阳汤治疗小儿肺气虚寒型慢性鼻窦炎临床研究

Clinical Study on the Treatment of Chronic Sinusitis of Lung Qi Deficiency and Cold Type in Children with Buqi Zhuyang Decoction. *亚太传统医药*, *16*(1), 136-138. https://doi.org/10.11954/ytctyy.202001047

舒柳青, & 朱健. (2020). 用头孢克肟联合藿胆片治疗急性化脓性鼻窦炎的临床效果

Effect of cefoxime combined with Epimedium bile tablets in the treatment of acute suppurative sinusitis. *当代医药论丛*, *18*(12), 195-197. https://doi.org/10.3969/j.issn.2095-7629.2020.12.124

徐保平. (2012). 香菊胶囊治疗小儿慢性鼻窦炎的临床观察. *中国实用医药*, *7*(14), 181-182. https://doi.org/10.3969/j.issn.1673-7555.2012.14.141

徐艳. (2018). 鼻渊汤治疗慢性鼻窦炎疗效观察. *饮食保健*, *5*(14), 93. https://doi.org/10.3969/j.issn.2095-8439.2018.14.109

徐艳芳. (2014). 通窍化痰清窦方治疗儿童鼻窦炎30例观察. *实用中医药杂志*, *30*(11), 1018.

徐英霞, 于磊, & 李会英. (2010). 鼻渊舒口服液在鼻窦内窥镜手术前后辅助治疗作用观察

Clinical observation of the accessory treatment effects of Nasosinusitis Relieving Oral Liquid before and after endoscopic surgery of sinus paranasales. *河北中医*, *32*(7), 1049-1051. https://doi.org/10.3969/j.issn.1002-2619.2010.07.054

徐豪杰, 苏梅, & 刘强. (2014). 观察中西医结合治疗小儿慢性鼻-鼻窦炎的临床疗效. *临床医药文献电子杂志*(8), 624-624,625. https://d.wanfangdata.com.cn/periodical/ChlQZXJpb2RpY2FsQ0hJTmV3UzIwMjEwNjE2EhFsY3l5ZHp6ejIwMTQwODE3NxoIY2c0dDFsaTE%3D

成月芹, & 麻晓晶. (2001). 中西药治疗慢性鼻窦炎的对比研究

Control Study on Traditional Chinese Medicine and Western Medicine Treating Chronic Nasosinusitis. *菏泽医专学报*, *13*(3), 60-62. https://doi.org/10.3969/j.issn.1008-4118.2001.03.027

苏东. (2016). 中西医结合在儿童急性鼻窦炎治疗中的疗效研究. *东方食疗与保健*(8), 40. https://doi.org/10.3969/j.issn.1672-5018.2016.08.031

关山越, 李卫红, 罗天飞, 李金奇, & 何植洲. (2009a). 慢性鼻窦炎鼻息肉鼻内镜术后中药辅助治疗的疗效观察. *中国实用医药*, *4*(23), 123-124. https://doi.org/10.3969/j.issn.1673-7555.2009.23.087

关山越, 李卫红, 罗天飞, 李金奇, & 何植洲. (2009b). 鼻渊汤治疗慢性鼻窦炎临床观察

Clinical Observation on the Nasosinusitis Relieving Decoction in the Treatment of Chronic Sinusitis Among 117 Cases. *亚太传统医药*, *5*(4), 64-65. https://d.wanfangdata.com.cn/periodical/ChlQZXJpb2RpY2FsQ0hJTmV3UzIwMjEwNjE2Eg95dGN0eXkyMDA5MDQwMzQaCGNqOWo2djY3

苏玉玲, 白莉, & 刘小亚. (2009). 苍耳子鼻炎胶囊配合西药治疗青少年鼻窦炎96例. *陕西中医*, *30*(4), 407-408. https://doi.org/10.3969/j.issn.1000-7369.2009.04.016

邵瑜. (2013). 鼻渊舒口服液治疗鼻窦炎的临床效果. *中外医学研究*(22), 72-72,73. https://doi.org/10.3969/j.issn.1674-6805.2013.22.045

邵长宝, 罗安平, 王立娟, & 邵腾皓. (2012). 中西医结合治疗慢性鼻-鼻窦炎疗效观察. *人民军医*, *55*(8), 736-737. https://d.wanfangdata.com.cn/periodical/ChlQZXJpb2RpY2FsQ0hJTmV3UzIwMjEwNjE2Eg1ybWp5MjAxMjA4MDI4GghjZzR0MWxpMQ%3D%3D

苏宗柳, 杨玉川, & 周和首. (2012). "苍耳子散加味"治疗儿童鼻窦炎86例临床观察. *江苏中医药*, *44*(8), 42-43. https://doi.org/10.3969/j.issn.1672-397X.2012.08.026

苏浩, & 李翠欣. (2002). 中西医结合治疗儿童慢性鼻窦炎100例临床分析. *山东医药*, *42*(16), 39. https://doi.org/10.3969/j.issn.1002-266X.2002.16.064

孙剑光. (2009). 中西医结合治疗慢性鼻窦炎162例分析. *当代医学*, *15*(2), 150-151. https://doi.org/10.3969/j.issn.1009-4393.2009.02.121

孙国军. (2014). 川芎茶调散加减治疗慢性上颌窦炎86例. *内蒙古中医药*, *33*(6), 8. https://d.wanfangdata.com.cn/periodical/ChlQZXJpb2RpY2FsQ0hJTmV3UzIwMjEwNjE2Eg9ubWd6eXkyMDE0MDYwMDkaCGNnNHQxbGkx

孙麦青, 蔡继堂, 张红伟, & 叶放蕾. (2006). 鼻舒乐丸治疗儿童慢性鼻窦炎疗效观察. *辽宁中医杂志*, *33*(3), 331-332. https://doi.org/10.3969/j.issn.1000-1719.2006.03.053

孙树军, 张爱玲, & 孙安琪. (2021). 疏风解毒胶囊联合鼻腔冲洗对鼻窦炎术后黏膜转归的影响. *中国处方药*, *19*(03), 114-115.

宋丽, & 高翠芳. (2004). 中西医结合治疗慢性鼻窦炎50例. *中国民间疗法*, *12*(9), 7. https://doi.org/10.3969/j.issn.1007-5798.2004.09.005

宋玉红, 赵雯, & 崔国玲. (2001). 中西医结合治疗急慢性鼻窦炎260例疗效观察. *山东医药*, *41*(16), 51-52. https://doi.org/10.3969/j.issn.1002-266X.2001.16.048

宋任婴. (2007). 鼻窦康复方治疗急、慢性鼻窦炎63例临床观察

Clinical Observation on the Treatment of 63 Cases of Acute or Chronic Sinusitis with Sinus Nasalis Recovery Prescription. *中医药导报*, *13*(6), 56,70. https://doi.org/10.3969/j.issn.1672-951X.2007.06.027

宋姝娇. (2010). 中西医结合治疗小儿慢性鼻窦炎临床疗效观察. *长治医学院学报*, *24*(5), 381-382. https://doi.org/10.3969/j.issn.1006-0588.2010.05.027

修世国, 叶辉, 白尚杰, & 姜宪. (2007). 鼻渊舒胶囊对功能性鼻窦内窥镜术后术腔黏膜修复的影响

Effect of BiYuanShu Capsule on Patients after Functional Endoscopic Sinus Surgery for Operated Area. *时珍国医国药*, *18*(5), 1195-1196. https://doi.org/10.3969/j.issn.1008-0805.2007.05.102

柴龙, 杨凯, 杜波, 卢家奉, 唐光俊, & 杨梅. (2019). 中药对慢性鼻-鼻窦炎患者术后恢复的影响研究. *中国保健营养*, *29*(27), 76. https://d.wanfangdata.com.cn/periodical/ChlQZXJpb2RpY2FsQ0hJTmV3UzIwMjEwNjE2EhJ6Z2JqeXkta3AyMDE5MjcwNDgaCGNqOWo2djY3

施正贤. (2017). 中西医结合治疗慢性鼻窦炎及合并眼部疾病临床观察. *新中医*, *49*(03), 101-103.

杨碧, & 赵萍. (2014). 玉屏风散加减合阿莫西林治疗鼻窦炎50例临床观察. *中国医药指南*(12), 270-271. https://d.wanfangdata.com.cn/periodical/ChlQZXJpb2RpY2FsQ0hJTmV3UzIwMjEwNjE2Eg96Z3l5em4yMDE0MTIyMTEaCGx0bXJvOGI2

杨宝琴, & 王天中. (2007). 苍耳子汤与麻杏石甘汤加减配合西药治疗鼻窦炎31例. *陕西中医*, *28*(8), 958-959. https://doi.org/10.3969/j.issn.1000-7369.2007.08.017

杨玉真. (2016). 鼻渊汤辨证加减治疗慢性鼻-鼻窦炎疗效观察. *中国处方药*, *14*(10), 97-98. https://doi.org/10.3969/j.issn.1671-945X.2016.10.071

杨静. (2012). 苍辛五苓散加减治疗鼻渊40例的临床观察

Clincal Abservation of Forty Cases of Nasosinusitis Therapy by Rexible application of the Recipe of Cang Xin Wu Ling San. *求医问药（学术版）*, *10*(2), 265-266. https://d.wanfangdata.com.cn/periodical/ChlQZXJpb2RpY2FsQ0hJTmV3UzIwMjEwNjE2EhFxeXd5LXhzYjIwMTIwMjI4MBoIY2c0dDFsaTE%3D

杨学文, & 邱波. (2009). 中西医结合治疗鼻炎临床观察. *山西中医*, *25*(7), 31-31. https://doi.org/10.3969/j.issn.1000-7156.2009.07.017

杨焕. (2016). 中医治疗慢性鼻窦炎的临床体会. *大家健康（中旬版）*, *10*(10), 30-31. https://d.wanfangdata.com.cn/periodical/ChlQZXJpb2RpY2FsQ0hJTmV3UzIwMjEwNjE2Eg9kamprLXoyMDE2MTAwMzkaCGNnNHQxbGkx

杨欣欣. (2017). 鼻渊通窍颗粒联合曲安奈德鼻喷雾剂对慢性鼻窦炎鼻纤毛传输功能的影响分析. *航空航天医学杂志*, *28*(08), 988-989.

严啸天, & 王旭. (2019). 补肺益脾通窍汤联合抗生素治疗慢性鼻窦炎的临床疗效. *中国实用医药*, *14*(05), 144-145.

严浩, & 谢桂华. (2017). 鼻窦炎应用中西医结合治疗的有效性探究. *中国社区医师*, *33*(04), 100+102.

余亚明. (2012). 藿胆丸配合鼻腔冲洗治疗慢性鼻窦炎68例. *河南中医*, *32*(05), 603-604.

余秋焕, & 杨春晓. (2009). 千金苇茎汤联合西药治疗儿童慢性鼻窦炎75例. *陕西中医*, *30*(7), 806-807. https://doi.org/10.3969/j.issn.1000-7369.2009.07.032

闫玲, & 周筱荣. (2013). 通窍汤治疗慢性上颌窦炎40例. *中医临床研究*, *5*(01), 64.

倪志军, 马文波, & 董丽婷. (2012). 慢性鼻窦炎术后服用鼻窦舒合剂的临床研究. *陕西中医学院学报*, *35*(1), 31-32. https://d.wanfangdata.com.cn/periodical/ChlQZXJpb2RpY2FsQ0hJTmV3UzIwMjEwNjE2EhFzeHp5eHl4YjIwMTIwMTAxNBoING40dHd3M3Y%3D

吴君, & 马殿伟. (2011). 苍耳子散合除湿汤加减对真菌性鼻窦炎术后的临床观察. *实用中西医结合临床*, *11*(3), 51,53. https://doi.org/10.3969/j.issn.1671-4040.2011.03.032

吴筱莉, 陆兴, 陈望燕, & 李永军. (2001). 中西医结合治疗儿童慢性上颌窦炎. *湖北中医杂志*, *23*(4), 18-19. https://doi.org/10.3969/j.issn.1000-0704.2001.04.013

吴小娟, 缪馨, 黄河银, & 唐泺. (2015). 白虎汤加味治疗急性鼻窦炎临床观察

@@. *四川中医*, *33*(8), 147-148. https://d.wanfangdata.com.cn/periodical/ChlQZXJpb2RpY2FsQ0hJTmV3UzIwMjEwNjE2Eg1zY3p5MjAxNTA4MDcyGghsdG1ybzhiNg%3D%3D

吴延涛, 封彦蕾, 张建新, & 梁月俏. (2017). 阿奇霉素合苍耳子鼻炎滴丸治疗儿童慢性鼻窦炎30例

To Observe the Curative Effect of Chronic Sinusitis in Children Treated with Azithromycin Xanthium Rhinitis Pills on 30 Cases. *中国民族民间医药*, *26*(10), 107-108. https://d.wanfangdata.com.cn/periodical/ChlQZXJpb2RpY2FsQ0hJTmV3UzIwMjEwNjE2EhN6Z216bWp5eXp6MjAxNzEwMDM3GghjZzR0MWxpMQ%3D%3D

吴周强, 田瑾, 马秉渊, 王文尊, & 李兴民. (1989). 香菊片治疗鼻窦炎的临床研究. *中成药*(8), 22-23. https://d.wanfangdata.com.cn/periodical/ChlQZXJpb2RpY2FsQ0hJTmV3UzIwMjEwNjE2Eg5RSzAwMDAwMzcwOTkyOBoING40dHd3M3Y%3D

吴志学, 郭伟, & 陈玉. (2000). *中西医结合治疗鼻窦炎(附122例疗效观察)(摘要)* 第三届第二次全国中西医结合耳鼻咽喉科学术大会论文汇编, 中国浙江宁波. https://d.wanfangdata.com.cn/conference/ChZDb25mZXJlbmNlTmV3UzIwMjEwNTIxEg5IWTAwMDAwMjAyMDU2OBoIY2c0dDFsaTE%3D

吴泽幼, 包思, 梁敬, & 许俊藩. (2017). 鼻渊通窍颗粒联合莫西沙星治疗急性鼻窦炎的临床研究. *现代药物与临床*, *32*(04), 657-660.

吴通照. (2013). 32例慢性鼻窦炎的中西医结合治疗效果观察. *中国医药指南*(18), 665-666. https://doi.org/10.3969/j.issn.1671-8194.2013.18.523

吴惠玲. (2011). 中西医结合治疗急性鼻窦炎60例疗效观察. *新中医*, *43*(11), 33. https://d.wanfangdata.com.cn/periodical/ChlQZXJpb2RpY2FsQ0hJTmV3UzIwMjEwNjE2Egx4enkyMDExMTEwMTcaCGNnNHQxbGkx

温富春. (2017). 西医结合治疗真菌性鼻窦炎疗效观察. *广西中医药*, *40*(1), 54-56. https://doi.org/10.3969/j.issn.1003-0719.2017.01.021

王锦辉. (2017). 鼻炎康汤治疗胆腑郁热型慢性鼻-鼻窦炎36例临床研究. *亚太传统医药*, *13*(4), 133-134. https://doi.org/10.11954/ytctyy.201704059

王令, 高宁, 孟英超, 姜少华, & 王树卯. (2015). 通窍固表汤配合鼻内镜手术治疗慢性鼻窦炎的临床观察. *河北医药*(16), 2505-2506. https://doi.org/10.3969/j.issn.1002-7386.2015.16.036

王利利. (2002). 中西医结合治疗慢性鼻窦炎48例. *山西中医*, *18*(1), 31. https://doi.org/10.3969/j.issn.1000-7156.2002.01.019

王利利. (2007). 上清丸治疗慢性鼻窦炎的疗效观察. *中国中西医结合耳鼻咽喉科杂志*, *15*(3), 222. https://doi.org/10.3969/j.issn.1007-4856.2007.03.025

王李民. (2010). 五味消毒饮加味治疗慢性鼻窦炎103例临床观察. *江苏中医药*, *42*(9), 37. https://doi.org/10.3969/j.issn.1672-397X.2010.09.025

王霖. (2014). 香菊胶囊联合罗红霉素治疗慢性鼻窦炎76例效果分析. *中国保健营养（下旬刊）*, *24*(7), 4190-4191. https://doi.org/10.3969/j.issn.1004-7484(x).2014.07.641

汪普, 赵俐菁, 张志利, & 付勇. (2015). 玉屏风散对老年慢性鼻窦炎患者炎症因子水平及免疫功能的影响. *中国老年学杂志*, *35*(08), 2134-2136.

王保霞, & 卢新阁. (2012). 小儿清肺化痰颗粒治疗儿童慢性鼻窦炎90例疗效观察. *河北中医*, *34*(8), 1211-1212. https://doi.org/10.3969/j.issn.1002-2619.2012.08.057

王凤志. (2012). 辛芩颗粒治疗100例儿童慢性鼻窦炎的疗效观察. *中国现代药物应用*, *6*(21), 71-71. https://doi.org/10.3969/j.issn.1673-9523.2012.21.059

王小华. (2011). 中西医结合治疗小儿慢性鼻-鼻窦炎49例. *中医药导报*, *17*(2), 40-42. https://doi.org/10.3969/j.issn.1672-951X.2011.02.019

王亚君. (2015). 鼻渊通窍颗粒辅助鼻内镜手术治疗慢性鼻－鼻窦炎临床研究. *现代中西医结合杂志*(24), 2678-2679,2684. https://doi.org/10.3969/j.issn.1008-8849.2015.24.021

王岩, 刘文忠, & 张晓明. (2008). 鼻炎汤在慢性鼻窦炎鼻息肉术后应用观察. *中国医疗前沿*, *3*(6), 93. https://doi.org/10.3969/j.issn.1673-5552.2008.06.068

王悦. (2018). 慢性鼻窦炎应用鼻渊通窍颗粒的治疗效果及对鼻纤毛传输功能的影响. *养生保健指南*(48), 247. https://doi.org/10.3969/j.issn.1006-6845.2018.48.239

汪月红, 徐盈, 程志娟, & 汪俊. (2008). 辛夷散治疗儿童鼻窦炎80例临床观察. *浙江中医杂志*, *43*(1), 42. https://doi.org/10.3969/j.issn.0411-8421.2008.01.025

王卫国. (2013). 中西医结合治疗慢性鼻窦炎的疗效分析. *健康之路*, *12*(10), 543-544. https://doi.org/10.3969/j.issn.1671-8801.2013.10.641

汪宁波. (2008). 托里消毒散治疗慢性溶水性上颌窦炎46例. *新中医*, *40*(1), 83-84. https://doi.org/10.3969/j.issn.0256-7415.2008.01.051

王宗杰. (2010). *中药鼻渊冲剂治疗急性鼻窦炎的临床疗效观察* [硕士, 黑龙江中医药大学].

王宗英. (2003). 中西医结合治疗慢性上颌窦炎45例疗效观察. *四川中医*, *21*(8), 93. https://doi.org/10.3969/j.issn.1000-3649.2003.08.071

王俊杰, 蔡纪堂, 李静波, 王慧敏, & 陈文明. (2020). 参苏温肺汤加减对慢性鼻-鼻窦炎患者的临床疗效

Clinical effects of Modified Sensu Wenfei Decoction on patients with chronic rhinosinusitis. *中成药*, *42*(2), 356-360. https://doi.org/10.3969/j.issn.1001-1528.2020.02.016

王中霞. (2008). *中药联合鼻窦负压置换治疗儿童肺脾气虚型慢性鼻窦炎的临床研究* [硕士, 福建中医学院].

王中霞. (2009). 中药治疗儿童慢性鼻窦炎的临床研究. *甘肃科技*, *25*(22), 161-162+119.

王中霞. (2016). 鼻渊丸对慢性鼻-鼻窦炎术后鼻黏膜恢复的影响

The Influence ofBiYuanPills on Nasal Mucous Membrane Recovery of the Patients after the Operation of Chronic Rhinosinusitis. *西部中医药*, *29*(5), 90-91,92. https://doi.org/10.3969/j.issn.1004-6852.2016.05.028

王志刚, & 吕荣林. (2013). 仙露贝联合通窍鼻炎胶囊治疗儿童慢性鼻窦炎疗效观察. *中国实用乡村医生杂志*, *20*(7), 53-54. https://doi.org/10.3969/j.issn.1672-7185.2013.07.038

王振鹏. (2014). 中西医结合治疗儿童慢性鼻窦炎临床观察. *光明中医*(9), 1914-1915. https://doi.org/10.3969/j.issn.1003-8914.2014.09.060

王振兴. (2017). 鼻内镜手术配合八珍汤加减方治疗慢性鼻-鼻窦炎临床研究. *实用中医药杂志*, *33*(12), 1394. https://doi.org/10.3969/j.issn.1004-2814.2017.12.038

王超仁. (2015). 加减参苓白术散治疗小儿慢性鼻窦炎的临床疗效. *北方药学*(5), 100-100. https://d.wanfangdata.com.cn/periodical/ChlQZXJpb2RpY2FsQ0hJTmV3UzIwMjEwNjE2Eg1iZnl4MjAxNTA1MDg1GghsdG1ybzhiNg%3D%3D

王春芳. (2019). 中药通窍鼻炎颗粒治疗急性鼻窦炎的临床效果分析. *健康之友*(11), 49-50. https://d.wanfangdata.com.cn/periodical/ChlQZXJpb2RpY2FsQ0hJTmV3UzIwMjEwNjE2Eg1qa3p5MjAxOTExMDU4GghjajlqNnY2Nw%3D%3D

王贤云. (2016). 观察中医体质辨证护理对慢性鼻窦炎患者的影响. *临床医药文献电子杂志*, *3*(22), 4425-4425,4428. https://d.wanfangdata.com.cn/periodical/ChlQZXJpb2RpY2FsQ0hJTmV3UzIwMjEwNjE2EhFsY3l5ZHp6ejIwMTYyMjA2NxoIbHRtcm84YjY%3D

王晓丽, & 刘乃锋. (2016). 分析中西医结合护理对慢性鼻窦炎患者鼻内镜术后恢复的影响. *中西医结合心血管病电子杂志*, *4*(23), 174. https://doi.org/10.3969/j.issn.2095-6681.2016.23.142

王晓燕, & 陈莉. (2020). 通窍鼻炎颗粒治疗儿童慢性鼻窦炎的效果探讨. *养生保健指南*(25), 245. https://d.wanfangdata.com.cn/periodical/ChlQZXJpb2RpY2FsQ0hJTmV3UzIwMjEwNjE2EhF5c2Jqem4teDIwMjAyNTI0MBoIY2o5ajZ2Njc%3D

王兴鸣. (2004). 中西医结合治疗慢性化脓性上颌窦炎90例

Chronic Suppurative Sinusitis Treated by Integrated TCM and WM. *陕西中医*, *25*(6), 519-519. https://doi.org/10.3969/j.issn.1000-7369.2004.06.029

姚期, 张艳萍, 鲍韦瑾, & 赵芳芳. (2016). 健脾化浊通窍汤治疗难治性慢性鼻-鼻窦炎80例的疗效观察. *中国地方病防治杂志*, *31*(06), 714+716.

姚岱君, 胡志红, 周蓓, & 叶茂果. (2007). 中西医结合治疗慢性鼻窦炎50例总结. *湖南中医杂志*, *23*(4), 35-36. https://doi.org/10.3969/j.issn.1003-7705.2007.04.018

姚望. (2016). 香菊胶囊治疗儿童慢性鼻窦炎90例疗效观察. *浙江中医杂志*, *51*(6), 423. https://doi.org/10.3969/j.issn.0411-8421.2016.06.019

姚秀丽. (2017). 加减鼻渊汤联合西医治疗慢性鼻-鼻窦炎的临床观察. *光明中医*, *32*(3), 411-412. https://doi.org/10.3969/j.issn.1003-8914.2017.03.048

姚稳根. (2013). 中西医结合治疗功能性内窥镜鼻窦炎手术后患者59例. *湖南中医杂志*, *29*(01), 68-69.

姚向东. (2010). 内镜鼻窦手术加中药治疗慢性鼻窦炎. *四川中医*, *28*(6), 104. https://d.wanfangdata.com.cn/periodical/ChlQZXJpb2RpY2FsQ0hJTmV3UzIwMjEwNjE2Eg1zY3p5MjAxMDA2MDU5GghsdG1ybzhiNg%3D%3D

牛玲, 周汝环, & 李武芬. (2014). 鼻渊通窍颗粒联合小儿诺通治疗儿童慢性鼻窦炎的疗效观察. *中医药临床杂志*, *26*(12), 1250-1251. https://d.wanfangdata.com.cn/periodical/ChlQZXJpb2RpY2FsQ0hJTmV3UzIwMjEwNjE2EhFhaHp5bGN6ejIwMTQxMjAyMBoIY2o5ajZ2Njc%3D

于凤英, 李长英, 王鹏善, & 薛世萍. (2020). 解表清里法联合西药治疗儿童急性鼻窦炎的疗效观察

Clinical Observation on Exterior-relieving Interior-clearing Therapeutic Method Combined with Western Medicine in the Treatment for Acute Pediatric Rhinitis-nasosinusitis. *西部中医药*, *33*(1), 119-121. https://doi.org/10.12174/j.issn.1004-6852.2020.01.31

于枫. (2019). 鼻渊汤加减与西药用于慢性鼻-鼻窦炎患者治疗中的临床效果. *中国现代药物应用*, *13*(11), 169-170.

郁再强, & 姚志源. (2016). 鼻渊丸治疗慢性鼻窦炎101例临床观察. *临床医学研究与实践*, *1*(23), 111,113. https://d.wanfangdata.com.cn/periodical/ChlQZXJpb2RpY2FsQ0hJTmV3UzIwMjEwNjE2EhJsY3l4eWp5c2oyMDE2MjMwNjMaCGNqOWo2djY3

熊慧. (2018). 藿胆片联合西药治疗急性鼻窦炎的临床观察. *健康必读*(15), 115. https://d.wanfangdata.com.cn/periodical/ChlQZXJpb2RpY2FsQ0hJTmV3UzIwMjEwNjE2EhBqa2JkMDAxMjAxODE1MTIyGghsdG1ybzhiNg%3D%3D

苑明茹. (2008). 中西医结合治疗慢性鼻窦炎62例疗效观察. *中外医疗*(13), 82.

喻青. (2004). *中西结合治疗慢性鼻窦炎148例* 2004年全国中西医结合耳鼻咽喉科学术会论文汇编, 南昌. https://d.wanfangdata.com.cn/conference/ChZDb25mZXJlbmNlTmV3UzIwMjEwNTIxEgc1ODAyMzcwGgg0bjR0d3czdg%3D%3D

张键铭. (2013). 鼻渊通窍颗粒治疗鼻窦炎66例. *中国药业*, *22*(4), 58-58. https://doi.org/10.3969/j.issn.1006-4931.2013.04.036

张珺珺, 忻耀杰, 滕磊, & 高旭青. (2012). 鼻内镜手术结合"加减八珍汤"治疗慢性鼻-鼻窦炎53例临床观察. *江苏中医药*, *44*(8), 41-42. https://doi.org/10.3969/j.issn.1672-397X.2012.08.025

张冬林. (2015). 儿童慢性鼻窦炎鼻内镜手术联合中药治疗效果观察. *中医临床研究*, *7*(21), 73-75.

张丽琴, 朱瑞先, 孙力, & 龙丽萍. (2009). 鼻渊通窍颗粒治疗儿童鼻及鼻窦炎的临床观察. *中国现代医生*, *47*(6), 67,69. https://doi.org/10.3969/j.issn.1673-9701.2009.06.034

张丽丽, & 刘肇杰. (2012). 中西医结合治疗小儿慢性鼻窦炎84例观察. *实用中医药杂志*, *28*(08), 667.

张丽艳. (2015). 鼻渊汤加减联合西药治疗慢性鼻-鼻窦炎的疗效观察. *陕西中医*, *36*(10), 1390-1391.

张磊. (2020). 自拟通窍止嗽散治疗过敏性鼻窦炎性久咳的效果评价. *中国现代药物应用*, *14*(12), 196-198. https://doi.org/10.14164/j.cnki.cn11-5581/r.2020.12.094

张慕然. (2006). 中西医结合治疗儿童慢性鼻窦炎临床观察. *辽宁中医药大学学报*, *8*(5), 108-108. https://doi.org/10.3969/j.issn.1673-842X.2006.05.076

张成科. (2010). 鼻渊方治疗慢性鼻窦炎36例临床观察

Clinical observation on 36 cases chronic sinusitis with formula of Biyuan. *中医临床研究*, *2*(14), 98-99. https://doi.org/10.3969/j.issn.1674-7860.2010.14.064

张素娜, 李谊, & 麻文来. (2014). 苍耳子鼻炎滴丸治疗慢性鼻-鼻窦炎的疗效观察. *河南医学研究*, *23*(3), 77-78. https://doi.org/10.3969/j.issn.1004-437X2014.03.037

张小华. (2011). 综合疗法治疗鼻窦炎鼻息肉80例临床分析. *健康必读（下旬刊）*(6), 72. https://d.wanfangdata.com.cn/periodical/ChlQZXJpb2RpY2FsQ0hJTmV3UzIwMjEwNjE2Eg9qa2JkLXgyMDExMDYwNzAaCDRuNHR3dzN2

张淑娟, & 严军虎. (2014). 霍胆丸治疗慢性鼻窦炎120例临床观察. *吉林医学*, *35*(27), 6012-6013.

张新友. (2010). 鼻渊康合剂治疗慢性鼻窦炎120例疗效分析. *中国社区医师(医学专业)*, *12*(17), 133. https://doi.org/10.3969/j.issn.1007-614x.2010.17.140

张雅珂. (2019a). 中西医结合治疗小儿慢性鼻窦炎临床研究. *实用中医药杂志*, *35*(12), 1513-1514.

张雅珂. (2019b). 中西医结合治小儿慢性鼻窦炎临床研究. *实用中医药杂志*, *35*(12), 1513-1514. https://d.wanfangdata.com.cn/periodical/ChlQZXJpb2RpY2FsQ0hJTmV3UzIwMjEwNjE2EhBzeXp5eXp6MjAxOTEyMDYzGghjajlqNnY2Nw%3D%3D

张艳娥, 张星星, 王山苓, & 张杰. (2016). 新安鼻渊方对慢性鼻—鼻窦炎术后患者生活质量的影响. *临床合理用药杂志*, *9*(10), 75-76.

张玉梅. (2019). 香菊胶囊治疗慢性鼻窦炎鼻息肉的疗效观察. *中国保健营养*, *29*(35), 82-83. https://d.wanfangdata.com.cn/periodical/ChlQZXJpb2RpY2FsQ0hJTmV3UzIwMjEwNjE2EhJ6Z2JqeXkta3AyMDE5MzUxMDMaCGNqOWo2djY3

张卫海, 李永新, & 李淼淼. (2017). 鼻窦炎口服液加鼻渊胶囊治疗儿童慢性鼻窦炎疗效观察. *中国保健营养*, *27*(10), 128-129. https://doi.org/10.3969/j.issn.1004-7484.2017.10.184

张一航. (2008). 鼻渊通窍颗粒治疗儿童慢性鼻窦炎临床观察. *中国误诊学杂志*, *8*(28), 6852-6853. https://doi.org/10.3969/j.issn.1009-6647.2008.28.035

张志亮, 邓智建, & 卢振民. (2010). 香菊胶囊治疗慢性鼻窦炎的疗效观察. *中国现代药物应用*, *4*(20), 130-131. https://doi.org/10.3969/j.issn.1673-9523.2010.20.120

张志亮, 曹冬梅, 侯梦霞, & 卢振民. (2011). 鼻渊通窍颗粒治疗急性鼻窦炎的疗效观察. *现代中西医结合杂志*, *20*(21), 2652-2653. https://doi.org/10.3969/j.issn.1008-8849.2011.21.031

张珍爱, & 李娟. (2020). 浅析中西医结合护理干预对鼻窦炎术后患者疼痛的影响. *光明中医*, *35*(5), 768-770. https://doi.org/10.3969/j.issn.1003-8914.2020.05.053

张春萍, 翟文生, 邓晓明, & 张建. (2006). 鼻窦解毒汤治疗小儿慢性鼻窦炎60例. *中医研究*, *19*(3), 35-36. https://doi.org/10.3969/j.issn.1001-6910.2006.03.015

张治, & 马钰辉. (2012). 鼻康片治疗慢性鼻窦炎的临床疗效. *健康必读（中旬刊）*, *11*(5), 261-262. https://d.wanfangdata.com.cn/periodical/ChlQZXJpb2RpY2FsQ0hJTmV3UzIwMjEwNjE2Eg9qa2JkLXoyMDEyMDUzODYaCDRuNHR3dzN2

张和平, 王勤学, & 於清. (2005). 综合治疗儿童慢性鼻窦炎92例疗效观察. *山西中医*, *21*(6), 20-21. https://doi.org/10.3969/j.issn.1000-7156.2005.06.012

张晓辉. (2015). 鼻窦炎应用中西医结合治疗的效果观察. *北方药学*, *12*(01), 41-42.

张屹. (2008). 中西医结合治疗慢性鼻窦炎45例临床观察. *湖南中医杂志*, *24*(6), 28-29. https://doi.org/10.3969/j.issn.1003-7705.2008.06.015

储进, & 周莉. (2014). 小儿鼻炎鼻窦炎保守治疗的临床价值研究. *中国继续医学教育*(4), 28-29. https://doi.org/10.3969/j.Issn.1674-9308.2014.04.017

宁晓阳. (2009). 苍耳子鼻炎胶囊在鼻内镜术后的疗效观察

Observation of curative effect of Changerzi Sinusitis Capsule after endoscopic sinus surgery. *现代医药卫生*, *25*(15), 2280-2281. https://d.wanfangdata.com.cn/periodical/ChlQZXJpb2RpY2FsQ0hJTmV3UzIwMjEwNjE2Eg94ZHl5d3MyMDA5MTUwMjcaCGNnNHQxbGkx

田红梅. (2011). 西结合法治疗慢性鼻窦炎的临床疗效观察

Treatment of Integrated Traditional and Western Clinical efficacy of chronic sinusitis. *中国中医药咨讯*, *3*(17), 32,34. https://d.wanfangdata.com.cn/periodical/ChlQZXJpb2RpY2FsQ0hJTmV3UzIwMjEwNjE2EhB6Z3p5eXp4MjAxMTE3MDE5GghsdG1ybzhiNg%3D%3D

郑国峰. (2016). 苍辛通窍汤联合常规西药治疗鼻窦炎临床观察. *新中医*, *48*(01), 111-112.

丁涛. (2012). 通窍止渊汤治疗慢鼻渊68例疗效观察. *医学理论与实践*, *25*(07), 804-805.

程琳, 徐丽蓉, & 魏文英. (2013). 通鼻汤治疗慢性鼻窦炎40例. *中国中医药现代远程教育*, *11*(11), 23-24. https://doi.org/10.3969/j.issn.1672-2779.2013.11.013

郑立友, 杨西国, & 刘民生. (2001). 内窥镜鼻窦术后屏风辛苍丸的疗效观察

The effect of Pingfengxincangwan for sinusitis and nasal polyp after endoscopy. *山东医大基础医学院学报*, *15*(4), 246,249. https://doi.org/10.3969/j.issn.1673-3770.2001.04.024

郑立友, & 张桂荣. (2001). 中西医结合治疗小儿慢性鼻窦炎. *山东中医杂志*, *20*(3), 157-158. https://doi.org/10.3969/j.issn.0257-358X.2001.03.020

郑水洁, & 赵勇. (2020). 疏风通窍汤联合罗伊护理对鼻窦炎术后疼痛及IgE、IFN-γ水平影响研究. *长春中医药大学学报*, *36*(06), 1189-1192.

郑顺昌, & 杨娟娟. (2010). 鼻渊通窍颗粒治疗儿童慢性鼻窦炎85例疗效观察. *山东医药*, *50*(37), 93. https://doi.org/10.3969/j.issn.1002-266X.2010.37.066

丁亚南, & 常向辉. (2016). 中药配合鼻内镜手术治疗鼻窦炎鼻息肉的疗效观察

Clinical Observation on Chinese Medicine combined with Endoscopic Sinus Surgery in the Treatment of Sinusitis and Nasal Polyps. *光明中医*, *31*(13), 1948-1949. https://doi.org/10.3969/j.issn.1003-8914.2016.13.053

齐鹤立. (2012). 慢性上颌窦炎临床治疗效果分析. *吉林医学*, *33*(28), 6090-6091.

赵江涛, & 王中霞. (2017). 通窍鼻炎丸治疗慢性鼻窦炎及其对相关炎性因子的影响

Effect of Tongqiao Biyan Pill in Treatment of Chronic Sinusitis and Related Inflammatory Factors. *山东中医杂志*, *36*(8), 663-665. https://d.wanfangdata.com.cn/periodical/ChlQZXJpb2RpY2FsQ0hJTmV3UzIwMjEwNjE2Eg9zZHp5enoyMDE3MDgwMTIaCGNnNHQxbGkx

赵璟晶. (2017). 鼻渊通窍颗粒治疗急性鼻窦炎的临床疗效分析. *大家健康（中旬版）*, *11*(5), 138-139. https://doi.org/10.3969/j.issn.1009-6019.2017.05.195

赵德渊, 沈晓玲, 张宁国, 石明海, & 王平. (2017). 中西医结合治疗慢性鼻窦炎46例临床分析. *青海医药杂志*, *47*(5), 75-76. https://d.wanfangdata.com.cn/periodical/ChlQZXJpb2RpY2FsQ0hJTmV3UzIwMjEwNjE2Eg9xaHl5enoyMDE3MDUwMzUaCGNqOWo2djY3

赵斌, 王献伟, & 李咏梅. (2015). 小儿鼻炎鼻窦炎的治疗体会. *世界最新医学信息文摘（连续型电子期刊）*(68), 115-115. https://doi.org/10.3969/j.issn.1671-3141.2015.68.102

赵雪松. (2016). 鼻渊通窍颗粒联合阿莫西林治疗急性鼻窦炎病例探究. *饮食保健*, *3*(12), 84-85. https://d.wanfangdata.com.cn/periodical/ChlQZXJpb2RpY2FsQ0hJTmV3UzIwMjEwNjE2Eg95aW5zYmoyMDE2MTIwOTIaCGNqOWo2djY3

赵爱霞, & 罗新南. (2016). 疏风通窍汤联合鼻内镜手术治疗小儿慢性鼻窦炎鼻息肉的疗效观察. *临床医药文献电子杂志*, *3*(22), 4402-4403.

赵燕茹. (2010). 自拟通窍止痛汤治疗慢性鼻窦炎90例临床观察. *国医论坛*, *25*(2), 30-31. https://doi.org/10.3969/j.issn.1002-1078.2010.02.024

赵威. (2013). *鼻渊合剂对FESS术后术腔黏膜修复作用的临床观察* [硕士, 南京中医药大学].

钟美雄, 利海燕, & 陈坚. (2020). 鼻渊通窍颗粒联合孟鲁司特钠咀嚼片治疗儿童慢性鼻窦炎临床观察

Observation on the curative effects of montelukast sodium chewable tablets combined with Biyuan Tongqiao Granules in the treatment of chronic sinusitis for children. *中国处方药*, *18*(2), 88-89. https://doi.org/10.3969/j.issn.1671-945X.2020.02.050

钟晓凤. (2012). 中西药结合治疗老年慢性鼻窦炎100例疗效分析. *求医问药（学术版）*, *10*(11), 639. https://d.wanfangdata.com.cn/periodical/ChlQZXJpb2RpY2FsQ0hJTmV3UzIwMjEwNjE2EhJxeXd5LXhzYjIwMTIxMTA2NzMaCGx0bXJvOGI2

周巧耘, 丘棱芝, & 陈雁晴. (2019). 玉屏风颗粒联合丙酸倍氯米松鼻喷雾剂治疗慢性鼻-鼻窦炎患者的疗效观察. *中医临床研究*, *11*(35), 15-17.

周军平, & 李玉芳. (2003). 中西医结合治疗慢性化脓性上颌窦炎48例. *河北中医*, *25*(3), 227-227. https://doi.org/10.3969/j.issn.1002-2619.2003.03.040

周李芳, 陈健, 刘真贞, & 杨华晖. (2017). 八珍汤加减方结合鼻内镜手术治疗慢性鼻-鼻窦炎临床研究. *亚太传统医药*, *13*(3), 116-117. https://doi.org/10.11954/ytctyy.201703047

周林荫. (2010). 小儿慢性鼻窦炎60例临床分析. *中国医药指南*, *8*(36), 245-246. https://doi.org/10.3969/j.issn.1671-8194.2010.36.188

周明义. (2015). 小儿鼻炎鼻窦炎保守治疗临床探讨. *母婴世界*(12), 110-111. https://d.wanfangdata.com.cn/periodical/ChlQZXJpb2RpY2FsQ0hJTmV3UzIwMjEwNjE2Eg1teXNqMjAxNTEyMTI2GghsdG1ybzhiNg%3D%3D

朱富高, 杨松凯, 朱士高, 张念凯, & 刘云超. (2004). 中药配合鼻内窥镜手术治疗鼻窦炎鼻息肉的疗效观察

Evaluation of healing effect for sinusitis and nasal polyps with traditional Chinese drug plus endoscopic sinus surgery. *山东大学基础医学院学报*, *18*(4), 220-221. https://doi.org/10.3969/j.issn.1673-3770.2004.04.011

朱扬秦. (2010). 中西医结合治疗慢性鼻窦炎90例疗效观察

Effective observation on treating 90 cases of sinusitis in the integrative medicine. *中医临床研究*, *2*(24), 70-70. https://doi.org/10.3969/j.issn.1674-7860.2010.24.047

朱颖兰. (2018). 中西医结合治疗300例儿童急性鼻窦炎疗效观察. *养生保健指南*(3), 88. https://doi.org/10.3969/j.issn.1006-6845.2018.03.082

周玉霞. *中西医结合治疗慢性上颌窦炎79例疗效观察* 中华中医药学会全国第十一届中医耳鼻喉科学术研讨会论文汇编, 成都. https://d.wanfangdata.com.cn/conference/ChZDb25mZXJlbmNlTmV3UzIwMjEwNTIxEgc2MDI0Njk5GghsdG1ybzhiNg%3D%3D

朱优立, 王琳, & 马崧. (2005). 川芎茶调颗粒冲剂治疗急性鼻窦炎的临床观察. *实用诊断与治疗杂志*, *19*(9), 669-669. https://doi.org/10.3969/j.issn.1674-3474.2005.09.024

周振峰, & 罗晓钦. (2007). 中西医结合治疗慢性鼻窦炎100例疗效观察. *湖南中医杂志*, *23*(5), 16-17. https://doi.org/10.3969/j.issn.1003-7705.2007.05.009

周灿禄, 曾平, 陈浩, 刘焕泰, 刘峰, & 邓钜良. (2007). 中药配合鼻内窥镜手术治疗慢性鼻窦炎、鼻息肉的疗效观察

Evaluation of effect for sinusitis and nasal polyps treated with traditional Chinese medicine plus endoscopic sinus surgery. *河北中医*, *29*(2), 112-113. https://doi.org/10.3969/j.issn.1002-2619.2007.02.006

朱洪源, 洪建和, & 吴国民. (2003). 中西医结合治疗小儿鼻窦炎116例临床分析

A clinical analysis on the therapeutic effect of integrative medicine therapy on chronic sinusitis among 116 children cases. *中国中西医结合耳鼻咽喉科杂志*, *11*(2), 64-65. https://doi.org/10.3969/j.issn.1007-4856.2003.02.005

朱晓朴, & 王旭. (2017). “醒窍汤”联合西药治疗慢性鼻-鼻窦炎48例临床研究. *江苏中医药*, *49*(11), 41-43.

朱晓朴, & 姚斌峰. (2021). 香菊胶囊联合罗红霉素治疗鼻窦炎的临床研究

Clinical Study of Xiangju Capsule Combined with Roxithromycin in the Treatment of Sinusitis. *中外医疗*, *40*(9), 178-180. https://doi.org/10.16662/j.cnki.1674-0742.2021.09.178

仲崇玉. (2012). 辛银鼻窦汤结合西药治疗慢性鼻窦炎疗效观察

Observation on Curative Effects ofXinYin BiDouTang and Western Medicine in the Treatment of Chronic Sinusitis. *西部中医药*, *25*(6), 76-78. https://doi.org/10.3969/j.issn.1004-6852.2012.06.030

仲崇玉. (2014). 辛银鼻窦汤治疗儿童慢性鼻窦炎的疗效研究. *陕西中医*(3), 289-290. https://doi.org/10.3969/j.issn.1000-7369.2014.03.020

曾伟. (2016). 中西医综合治疗慢性鼻窦炎与眼部相关疾病相关性的临床观察

Clinical Observation of the Correlation of Treating Chronic Nasosinusitis and Ocular Diseases with Chinese and Western Medicine. *数理医药学杂志*, *29*(3), 350-351,352. https://doi.org/10.3969/j.issn.1004-4337.2016.03.015

曾屹生, 唐月英, 陈宇, 张国庆, 许亚桦, 王丽云, & 郑晓雯. (2016). 从黄芩滑石汤探讨利湿通窍法对脾胃湿热证型慢性鼻窦炎术后疗效

The effect of Huangqin Huashi decoction on chronic sinusitis with damp-heat syndrome of spleen-stomach after surgery. *山西中医学院学报*, *17*(6), 34-36. https://doi.org/10.3969/j.issn.1000-7369.2016.06.013

陈启文. (2014). 中西医结合治疗成年慢性鼻窦炎体会. *临床医药文献电子杂志*(10), 1692-1693. https://d.wanfangdata.com.cn/periodical/ChlQZXJpb2RpY2FsQ0hJTmV3UzIwMjEwNjE2EhFsY3l5ZHp6ejIwMTQxMDAxMxoIY2c0dDFsaTE%3D

陈桂才, & 何桂娟. (2012). 鼻渊通窍颗粒治疗儿童鼻窦炎500例体会. *西南军医*, *14*(2), 265. https://doi.org/10.3969/j.issn.1672-7193.2012.02.039

陈国祥. (2014). 中西医结合治疗儿童急性鼻窦炎临床观察. *新中医*, *46*(12), 139-140.

陈琦, 胡金旺, 王刚, & 吴长松. (2014). 鼻内镜手术后联合鼻渊通窍颗粒治疗慢性鼻鼻窦炎的临床研究. *医药前沿*(24), 124-124. https://doi.org/10.3969/j.issn.2095-1752.2014.24.116

陈毛毛, & 肖洁. (2018). 口服鼻渊通窍颗粒对小儿慢性鼻窦炎的疗效观察

Therapeutic Effect of Oral Biyuan Tongqiao Granule on Chronic Sinusitis inChildren. *国际感染杂志（电子版）*, *7*(3), 81-83. https://d.wanfangdata.com.cn/periodical/ChlQZXJpb2RpY2FsQ0hJTmV3UzIwMjEwNjE2EhFnamdyenotZDIwMTgwMzA1NRoIY2o5ajZ2Njc%3D

陈文明. (2015). *培土生金法在FESS患者术后运用的价值初探* 世界中联耳鼻喉口腔专业委员会换届大会暨第七次学术年会、中华中医药学会耳鼻喉分会第二十一次耳鼻喉科学术年会暨辽宁省中医及中西医结合耳鼻咽喉科学术会议论文集, 沈阳. https://d.wanfangdata.com.cn/conference/ChZDb25mZXJlbmNlTmV3UzIwMjEwNTIxEgc4ODI4NDY1Ggg0bjR0d3czdg%3D%3D

陈福彬, 朱继先, & 薛秉阳. (2002). 鼻渊冲剂治疗慢性鼻窦炎132例

132 Cases of Chronic Nasosinusitis treated by BiYuan Granules. *辽宁中医学院学报*, *4*(2), 112-113. https://doi.org/10.3969/j.issn.1673-842X.2002.02.018

陈思法. (2012). 观察中西医结合治疗小儿慢性鼻窦炎的临床疗效. *按摩与康复医学（下旬刊）*, *3*(11), 346-346. https://d.wanfangdata.com.cn/periodical/ChlQZXJpb2RpY2FsQ0hJTmV3UzIwMjEwNjE2EhJhbXlrZnl4LXgyMDEyMTEzODMaCGNnNHQxbGkx

陈少茹, & 王挥戈. (2005). 鼻渊舒加强力稀化粘素治疗儿童慢性鼻窦炎

The therapeutic effect of a therapy combined Nasosinusitis Relieving Oral Liquid with Gelomyrtol Forte on chronic sinusitis among children. *中国中西医结合耳鼻咽喉科杂志*, *13*(4), 206-208. https://doi.org/10.3969/j.issn.1007-4856.2005.04.010

陈松岳, 杨桦, 顾亮, & 许敏达. (2009). 鼻渊舒口服液对慢性鼻窦炎和鼻息肉术后患者睡眠结构的影响. *中国中医药科技*, *16*(3), 227-228. https://doi.org/10.3969/j.issn.1005-7072.2009.03.031

陈亚军, 吴莉, 张健, 王红林, 王宗定, & 曾光. (2016). 香菊胶囊配合负压置换治疗小儿鼻窦炎500例

XiangJu Capsules Combined with Proetz Treatment in Treating 500 Children with Sinusitis. *西部中医药*, *29*(6), 80-81. https://doi.org/10.3969/j.issn.1004-6852.2016.06.030

陈全生. (2015). 鼻渊通窍颗粒治疗鼻窦炎的疗效观察. *中外医学研究*, *13*(31), 131-132.

陈婷婷, 郭其云, 刘佳琪, & 刘丽庭. (2017). 龙胆泻肝汤结合西药治疗慢性鼻-鼻窦炎不伴鼻息肉患者的临床观察

Efficacy observation of longdan xiegan decoction combined with western medicine in chronic rhinosinusitis without nasal polyps. *中国中西医结合耳鼻咽喉科杂志*, *25*(2), 94-98. https://doi.org/10.16542/j.cnki.issn.1007-4856.2017.02.005

陈正辉. (2010). 参苓白术散加减方治疗鼻窦炎术后黏膜水肿18例观察. *实用中医药杂志*, *26*(11), 758-759.

陈俊杰, 林傧, & 林建武. (2005). 通窍鼻炎片对慢性鼻窦炎、鼻息肉鼻内窥镜术后病例的临床疗效观察. *福建医药杂志*(02), 74-76.

陈倩倩. (2016). 中西医结合治疗慢性鼻窦炎的疗效与护理. *中西医结合心血管病电子杂志*, *4*(21), 174-175.

陈春华, 杨金锁, & 唐成定. (2009). 辛散通窍汤治疗慢性鼻窦炎33例观察. *实用中医药杂志*, *25*(06), 360.

陈忠万, & 刘爱华. (2013). 中西医结合治疗慢性鼻窦炎218例临床观察. *中国中西医结合耳鼻咽喉科杂志*, *21*(2), 143. https://doi.org/10.3969/j.issn.1007-4856.2013.02.026

陈向军, 黄健健, & 李国义. (2015). 玉屏风散联合头孢丙烯治疗急性细菌感染性鼻窦炎的疗效和安全性分析

Curative Efficience and Safety Analysis of Jade Screen Power with Cefprozil in Treating Acute Bacterial Sinusitis. *中国医药导刊*(6), 589-590,593. https://doi.org/10.3969/j.issn.1009-0959.2015.06.020

陈华. (2016). 鼻渊通窍经验方联合头孢克肟胶囊治疗慢性鼻窦炎疗效观察. *新中医*, *48*(08), 201-202.

蔡群峰, & 张德江. (2007). 慢性鼻窦炎术后中药辅助治疗的临床基础研究

Therapeutic Effect of Bi -yuan- shu Liquid on Patients with Chronic Nasal Sinusitis After Endoscopic Sinus Surgery. *成都中医药大学学报*, *30*(4), 13-15. https://doi.org/10.3969/j.issn.1004-0668.2007.04.006

蔡燕文, 张雪玲, & 李彩云. (2011). 鼻敏灵联合丙酸氟替卡松喷鼻治疗儿童慢性鼻窦炎的疗效观察. *湖北中医杂志*, *33*(5), 20-21. https://doi.org/10.3969/j.issn.1000-0704.2011.05.011

蔡楚君. (2019). *甘露消毒丹治疗脾胃湿热型慢性鼻-鼻窦炎随机对照试验* [硕士, 新疆医科大学].

楚选云. (2017). 鼻渊通窍颗粒联合曲安奈德鼻喷雾剂治疗慢性鼻-鼻窦炎的疗效及对鼻纤毛传输功能的影响. *世界中医药*, *12*(10), 2382-2385.

楚选云, & 高建步. (2018). 鼻渊通窍颗粒治疗慢性鼻-鼻窦炎的效果及对鼻纤毛传输功能的影响. *世界中医药*, *13*(05), 1191-1194.

肖艳林. (2014). 低温等离子技术配合中药治疗慢性鼻窦炎的临床疗效

The clinical effect of low temperature plasma technology combined with traditional Chinese medicine in the treatment of chronic nasosinusitis. *中国社区医师*(16), 82-83. https://doi.org/10.3969/j.issn.1007-614x.2014.16.54

焦河玲, & 唐成定. (2008). "辛芳鼻炎汤"治疗慢性鼻窦炎300例临床观察. *江苏中医药*, *40*(10), 53. https://doi.org/10.3969/j.issn.1672-397X.2008.10.028

肖辉良, & 黄合银. (2011). 中西医结合治疗儿童慢性鼻窦炎64例临床观察

Therapeutic Effect Observation of 64cases on the combination of TCM with Western medicine in Treatment the Chronicsin usitisin children. *内蒙古中医药*, *29*(9), 64-65. https://doi.org/10.3969/j.issn.1006-0979.2011.09.063

崔庆霞. (2010). 中西医结合治疗慢性鼻窦炎90例. *中国中医药现代远程教育*, *8*(19), 36-37. https://doi.org/10.3969/j.issn.1672-2779.2010.19.034

崔珑. (2016). 蒲地蓝口服液联合西药治疗儿童慢性鼻－鼻窦炎52例. *中国药业*, *25*(2), 110-111. https://d.wanfangdata.com.cn/periodical/ChlQZXJpb2RpY2FsQ0hJTmV3UzIwMjEwNjE2Eg16Z3l5MjAxNjAyMDQzGghsdG1ybzhiNg%3D%3D

邹桂年, 曾旭军, & 魏海敏. (2016). 儿童鼻窦炎中西医结合治疗临床研究. *中国实用医药*, *11*(19), 197-198. https://doi.org/10.14163/j.cnki.11-5547/r.2016.19.144

沈泽元, & 况光仪. (2000). 中西医结合治疗鼻窦炎65例. *海南医学*, *11*(3), 22,22. https://doi.org/10.3969/j.issn.1003-6350.2000.03.017

彭有全. (2018). 慢性鼻-鼻窦炎患者鼻内镜术后口服鼻渊通窍颗粒效果观察. *基层医学论坛*, *22*(35), 4954-4955.

彭冲, 陈勇辉, & 张国川. (2019). 鼻渊通窍颗粒联合曲安奈德鼻喷雾剂治疗慢性鼻—鼻窦炎的临床疗效及安全性分析. *医学理论与实践*, *32*(20), 3309-3310.

彭寒. (2014). 慢性鼻窦炎患者内镜术后中医辨证治疗的疗效分析

The efficacy analysis of TCM treatment for chronic sinusitis following endoscopic surgery. *中医临床研究*(8), 28-29. https://doi.org/10.3969/j.issn.1674-7860.2014.08.011

何勉, & 彭璐璐. (2008). 千金苇茎汤加减治疗小儿鼻窦炎临床观察. *四川中医*, *26*(3), 110-110. https://doi.org/10.3969/j.issn.1000-3649.2008.03.077

何春玲. (2013). 银花通窍鼻炎汤治疗慢性鼻窦炎的临床疗效观察. *中国医药指南*, *11*(20), 289-290.

何春玲. (2019). 中西医结合治疗儿童慢性鼻窦炎疗效观察. *实用中医药杂志*, *35*(08), 992-993.

向雅倩, 曹恒, & 姚平. (2010). 中西医结合治疗慢性鼻窦炎80例临床观察

Clinical Observation on Treatment of 80 Cases of Chronic Sinusitis with Chinese and Western Medicine. *江苏中医药*, *42*(2), 34-35. https://doi.org/10.3969/j.issn.1672-397X.2010.02.020

向彩云. (2011). 医结合治疗小儿慢性鼻窦炎93例疗效观察. *中国当代医药*, *18*(30), 96-97. https://doi.org/10.3969/j.issn.1674-4721.2011.30.051

许瑞凌. (2015). 藿胆滴丸联合头孢米诺治疗急性化脓性鼻窦炎58例疗效观察

Observation of Efficacy of 58 Cases of Huodan Dripping Pills Combined with Cefminox for Treatment of Acute Purulent Sinusitis. *襄阳职业技术学院学报*(2), 35-36. https://doi.org/10.3969/j.issn.1671-914X.2015.02.010

许秀霞, 冯喆, & 姜轶铭. (2014). 中西医结合治疗鼻窦炎的临床效果观察

Analysis the Clinical Effect of Combining Traditional Chinese and Western Medicine Treatment of Sinusitis. *中国伤残医学*(21), 20-21. https://doi.org/10.13214/j.cnki.cjotadm.2014.21.015

许彩萍. (2018). 鼻渊通窍颗粒辅助治疗急性鼻窦炎的疗效观察. *心理医生*, *24*(1), 130-131. https://d.wanfangdata.com.cn/periodical/ChlQZXJpb2RpY2FsQ0hJTmV3UzIwMjEwNjE2Eg94bHlzLXgyMDE4MDExMDcaCGNqOWo2djY3

胡启煜. (2019). 加味千金苇茎汤治疗肺经热盛型急性鼻窦炎临床研究. *光明中医*, *34*(08), 1149-1151.

胡文健. (2005). 中西医结合治疗鼻窦炎74例疗效观察. *胡南中医杂志*, *21*(5), 38-39. https://doi.org/10.3969/j.issn.1003-7705.2005.05.020

胡生辉. (2012). 鼻炎宁颗粒在慢性鼻窦炎鼻息肉围手术期应用的疗效观察. *南通大学学报（医学版）*, *32*(4), 328-329. https://doi.org/10.3969/j.issn.1674-7887.2012.04.038

胡胜祥, 李湘, & 黄剑聪. (2011). 联合加味六君子汤治疗鼻窦炎术后黏膜水肿疗效观察

Clinical observation on treating mucosal edema after sinusitis surgery with Liujunzi decoction. *中医临床研究*, *3*(9), 8-10. https://doi.org/10.3969/j.issn.1674-7860.2011.09.004

胡顺生, & 刘军波. (2007). 中西医结合治疗慢性鼻窦炎鼻息肉59例总结

A Summary on 59 Cases of Chronic Sinusitis and Rhinopolypus Treated with the Therapy of Combination of Chinese Traditional and Western Medicine. *湖南中医杂志*, *23*(5), 5-7. https://doi.org/10.3969/j.issn.1003-7705.2007.05.003

胡俊杰. (2013). 中西医结合治疗儿童慢性鼻窦炎临床研究. *亚太传统医药*, *9*(12), 96.

洪元庚. (2010). 低温消融术配合中药治疗慢性鼻窦炎58例观察. *实用中医药杂志*, *26*(09), 628.

洪静. (2010). *功能性鼻内镜术后应用菊花通圣汤疗效观察* [硕士, 辽宁中医药大学].

洪海裕, 陈奕伸, 洪仲思, & 郑晓滨. (2015). 鼻渊通窍颗粒联合克拉霉素治疗不伴息肉的慢性鼻-鼻窦炎疗效观察

@@. *中药材*, *38*(6), 1334-1336. https://doi.org/10.13863/j.issn1001-4454.2015.06.057

黄桂锋. (2019). 温阳化湿法治疗慢性鼻-鼻窦炎伴鼻息肉临床分析. *光明中医*, *34*(10), 1477-1479.

黄魁, & 王黎. (2011). 小儿鼻炎的临床治疗分析. *求医问药(下半月)*, *9*(11), 178-179.

黄业武, & 黎国杰. (2015). 自拟中药方用于慢性鼻窦炎术后疗效观察. *广西中医药*, *38*(2), 21-22. https://d.wanfangdata.com.cn/periodical/ChlQZXJpb2RpY2FsQ0hJTmV3UzIwMjEwNjE2Eg5neHp5eTIwMTUwMjAwORoIY2c0dDFsaTE%3D

黄业武, 冯时滨, 李东, & 李杰恩. (2015). 自拟中药内服在慢性鼻窦炎鼻内镜术后的辨证施治效果分析

Analysis of syndrome differentiation treatment effect of self-made traditional Chinese herbs for chronic sinusitis after endoscopic surgery. *右江医学*, *43*(3), 329-333. https://doi.org/10.3969/j.issn.1003-1383.2015.03.016

黄延昌. (2016). 补肺益脾通窍汤治疗慢性鼻窦炎39例疗效观察

@@. *中国中医药科技*, *23*(2), 183,187. https://d.wanfangdata.com.cn/periodical/ChlQZXJpb2RpY2FsQ0hJTmV3UzIwMjEwNjE2EhB6Z3p5eWtqMjAxNjAyMDI2GghjZzR0MWxpMQ%3D%3D

黄永平. (2013). 中西医结合治疗慢性鼻窦炎148例. *中国中医药现代远程教育*, *11*(18), 64-64. https://doi.org/10.3969/j.issn.1672-2779.2013.18.048

黄春江. (2010). 中药治疗鼻内镜围手术期疗效观察. *中国社区医师(医学专业)*, *12*(15), 114.

黄向红. (2012). 龙胆泻肝汤加减治疗小儿急性鼻窦炎40例临床观察. *新中医*, *44*(3), 71-72. https://d.wanfangdata.com.cn/periodical/ChlQZXJpb2RpY2FsQ0hJTmV3UzIwMjEwNjE2Egx4enkyMDEyMDMwMzYaCGx0bXJvOGI2

黄慧敏, & 覃文格. (2011). 自拟鼻渊通窍饮治疗慢性鼻窦炎82例临床观察. *中国民族民间医药*, *20*(6), 67-67. https://doi.org/10.3969/j.issn.1007-8517.2011.06.048

侯森, & 宫丽丽. (2014). 鼻渊汤辨证加减联合西医治疗慢性鼻-鼻窦炎的临床观察

Clinical observation on therapeutic effect of a combined therapy with modified Biyuan Decoction on the basis of syndrome differentiation with medical treating procedures for the treatment of chronic rhinosinusitis. *中国中西医结合耳鼻咽喉科杂志*(3), 179-181. https://doi.org/10.3969/j.issn.1007-4856.2014.03.006

侯寿尧. (2011). 中西医结合综合治疗慢性化脓性副鼻窦炎. *中国社区医师(医学专业)*, *13*(34), 182. https://doi.org/10.3969/j.issn.1007-614x.2011.34.179

侯自强. (2009). 连蒲苍耳子散联合药物冲洗治疗慢性鼻窦炎138例疗效观察. *中医药导报*, *15*(8), 52-54. https://doi.org/10.3969/j.issn.1672-951X.2009.08.029

侯清惠, 聂志君, 李晓玲, & 许焜. (2015). 自拟益气通窍汤治疗慢性鼻窦炎疗效观察. *大家健康（中旬版）*(3), 42-42. https://d.wanfangdata.com.cn/periodical/ChlQZXJpb2RpY2FsQ0hJTmV3UzIwMjEwNjE2Eg9kamprLXoyMDE1MDMwMzkaCGNqOWo2djY3

侯宪保, & 张兆星. (2002). 中西医结合治疗儿童慢性鼻窦炎. *山东大学基础医学院学报*(04), 207-208.

侯红岭. (2020). 分析自拟辛夷鼻炎汤治疗鼻渊的临床疗效. *中华养生保健*, *38*(2), 22-23. https://d.wanfangdata.com.cn/periodical/ChlQZXJpb2RpY2FsQ0hJTmV3UzIwMjEwNjE2Eg96aHlzYmoyMDIwMDIwMTIaCGNqOWo2djY3

**3. not about chronic rhinosinusitis (n = 21)**

甘海燕. (2019). 鼻窦炎口服液联合西药治疗急性鼻窦炎的临床观察. *中国医药指南*, *17*(11), 204-205. https://d.wanfangdata.com.cn/periodical/ChlQZXJpb2RpY2FsQ0hJTmV3UzIwMjEwNjE2Eg96Z3l5em4yMDE5MTExNTgaCGNnNHQxbGkx

邱红艳, & 陈玲. (2019). 银黄清肺胶囊联合阿奇霉素治疗肺经风热型急性鼻窦炎临床研究. *新中医*, *51*(05), 238-240.

刘鲜妮, & 张瑞永. (2016). 鼻窦炎汤配合鼻内镜手术治疗非侵袭型真菌性鼻-鼻窦炎疗效观察

Observation on the Effect of Non Invasive Fungal Rhinosinusitis Treated with Bidouyan Decoction Combined with Nasal Endoscopic Sinus Surgery. *山东中医杂志*, *35*(5), 399-401. https://d.wanfangdata.com.cn/periodical/ChlQZXJpb2RpY2FsQ0hJTmV3UzIwMjEwNjE2Eg9zZHp5enoyMDE2MDUwMTEaCGNnNHQxbGkx

刘静, & 朱佳丽. (2019). 鱼脑石鼻渊汤治疗儿童鼻窦炎50例疗效观察. *医药前沿*, *9*(31), 216-217. https://d.wanfangdata.com.cn/periodical/ChlQZXJpb2RpY2FsQ0hJTmV3UzIwMjEwNjE2Eg55aXlxeTIwMTkzMTE5NRoIY2o5ajZ2Njc%3D

刘慧, & 许昱. (2016). 健脾化浊通窍法联合鼻内镜手术治疗以涕多为主症鼻窦炎临床观察

@@. *四川中医*, *34*(12), 108-110. https://d.wanfangdata.com.cn/periodical/ChlQZXJpb2RpY2FsQ0hJTmV3UzIwMjEwNjE2Eg1zY3p5MjAxNjEyMDQxGgg0bjR0d3czdg%3D%3D

李静波. (2015). 中西医结合治疗变应性真菌性鼻窦炎疗效观察

Allergic Fungal Sinusitis Efficacy in Integrative Chinese and Western Medicine Therapy. *中国继续医学教育*(30), 195-196. https://doi.org/10.3969/j.issn.1674-9308.2015.30.144

庞敏飞. (2015). 苍耳鼻窦炎方联合头孢米诺钠治疗化脓性鼻窦炎临床观察. *新中医*, *47*(10), 153-154. https://doi.org/10.13457/j.cnki.jncm.2015.10.072

付发祥, & 赵鉴. (2020). 疏风解毒胶囊治疗急性鼻窦炎的疗效观察. *中国中医急症*, *29*(10), 1844-1846. https://doi.org/10.3969/j.issn.1004-745X.2020.10.043

苏金辉, 陈远安, & 欧桂养. (2016). 头孢呋辛联合鼻渊软胶囊治疗急性鼻窦炎临床疗效分析. *北方药学*, *13*(04), 55-56.

杨春玲, 芦金男, & 张海芳. (2018). 清热化浊通窍汤辅助治疗小儿急性鼻窦炎41例临床观察

Clinical observation on 41 cases of children with acute nasosinusitis treated with Qingre Huazhuo Tongqiao Tang as an adjunctive therapy. *中医儿科杂志*, *14*(2), 49-51. https://doi.org/10.16840/j.issn1673-4297.2018.02.15

易美涛. (2018). 鼻内镜手术联合加味玉屏风散治疗真菌性鼻窦炎的临床观察. *中国中医急症*, *27*(3), 513-515. https://doi.org/10.3969/j.issn.1004-745X.2018.03.042

王吉国. (2021). 观察分析苍辛通窍汤治疗鼻窦炎的临床效果评价. *特别健康*(13), 121. https://d.wanfangdata.com.cn/periodical/ChlQZXJpb2RpY2FsQ0hJTmV3UzIwMjEwNjE2Eg1qdGJqMjAyMTEzMTIyGghjajlqNnY2Nw%3D%3D

张立刚. (2015). 通窍鼻炎颗粒联合布地奈德治疗鼻窦炎97例

Ninety-seven Patients with Nasosinusitis Treated with Tongqiao Biyan Granules Combined with Budesonide. *河南中医*, *35*(9), 2288-2290. https://doi.org/10.16367/j.issn.1003-5028.2015.09.0970

张鸿雁. (2020). 香菊胶囊结合莫西沙星治疗鼻窦炎临床疗效及安全性研究. *国际感染病学(电子版)*, *9*(02), 106.

赵龙飞. (2016). *银翘清鼻胶囊治疗儿童急性鼻窦炎风热犯肺证临床疗效观察* [硕士, 山西中医学院].

钟敏茹, 邱宝珊, 李丽君, & 林叶青. (2020). 黄芩滑石汤治疗急性鼻窦炎的疗效观察. *中国中医急症*, *29*(09), 1625-1627.

周文康, & 王红月. (2020). 鼻窦炎鼻内窥镜围手术期中西医结合治疗的临床疗效分析. *临床医药文献电子杂志*, *7*(24), 50. https://d.wanfangdata.com.cn/periodical/ChlQZXJpb2RpY2FsQ0hJTmV3UzIwMjEwNjE2EhFsY3l5ZHp6ejIwMjAyNDA0MBoIY2o5ajZ2Njc%3D

朱宁, 姚晓燕, & 王志平. (2018). 川芎茶调散合苍耳子散联合西医常规疗法治疗急性鼻窦炎临床研究

Chuanxiong-Chatiao powder and Cangerzi powder combined with routine treatment for acute rhinosinusitis. *国际中医中药杂志*, *40*(8), 719-721. https://doi.org/10.3760/cma.j.issn.1673-4246.2018.08.009

周青秀. (2020). 健脾理肺方联合鼻内镜与西药治疗真菌性鼻窦炎52例临床研究. *新中医*, *52*(04), 96-98.

胡卫华, & 万里江. (2019). 口服藿胆片辅助治疗急性化脓性鼻窦炎患者的价值研究. *临床医药文献电子杂志*, *6*(71), 70-71. https://d.wanfangdata.com.cn/periodical/ChlQZXJpb2RpY2FsQ0hJTmV3UzIwMjEwNjE2EhFsY3l5ZHp6ejIwMTk3MTA1ORoIY2c0dDFsaTE%3D

黄瑛. (2014). 鼻渊通窍颗粒联合阿莫西林治疗急性鼻窦炎随机平行对照研究

Acute Sinusitis Randomized Parallel Group Study Biyuan Tongqiao Particles Combined Particle Therapy Amoxicillin. *实用中医内科杂志*, *28*(6), 137-138. https://doi.org/10.13729/j.issn.1671-7813.2014.06.66

**4. without description of individual herb component (n = 13)**

康宁. *辛芩颗粒治疗慢性鼻窦炎的临床总结* 中华中医药学会全国第十一届中医耳鼻喉科学术研讨会论文汇编, 成都. https://d.wanfangdata.com.cn/conference/ChZDb25mZXJlbmNlTmV3UzIwMjEwNTIxEgc2MDI0NDYxGgg0bjR0d3czdg%3D%3D

刘宪宾. (2017). 香菊胶囊辅助治疗慢性鼻窦炎鼻息肉49例疗效观察. *北方药学*, *14*(2), 58-59. https://doi.org/10.3969/j.issn.1672-8351.2017.02.050

裴嘉红. (2005). *云南白药胶囊围手术期用药减少慢性鼻窦炎鼻息肉鼻内镜手术中出血随机双盲、对照、多中心临床研究* [硕士, 四川大学].

徐志远. (2019). 鼻渊通窍颗粒联合头孢地尼分散片对 儿童慢性鼻窦炎的临床疗效观察

Observation on clinical efficacy of Biyuan Tongqiao granule combined with cefdinir dispersible tablets on chronic sinusitis in children. *中国实用医药*, *14*(28), 17-18. https://doi.org/10.14163/j.cnki.11-5547/r.2019.28.007

雍军, & 王咏峰. (2016). 鼻渊通窍颗粒联合曲安奈德鼻喷雾剂治疗慢性鼻-鼻窦炎的临床观察Δ

Clinical Observation of Biyuan Tongqiao Granule Combined with Triamcinolone Acetonide Spray in the Treatment of Chronic Rhinosinusitis. *中国药房*, *27*(20), 2795-2797. https://doi.org/10.6039/j.issn.1001-0408.2016.20.18

汪身正. (2020). 香菊胶囊联合枸地氯雷他定 对慢性鼻窦炎患者黏膜组织重塑及炎症反应的影响

Effect of Xiangju capsule combined with desloratadine citrate on mucosal tissue remodeling and inflammatory response in patients with chronic sinusitis. *天津药学*, *32*(4), 36-39. https://doi.org/10.3969/j.issn.1006-5687.2020.04.013

饶小桥. (2019). 鼻渊通窍颗粒联合曲安奈德鼻喷雾剂治疗慢性鼻-鼻窦炎的临床价值探究. *养生保健指南*(15), 71. https://doi.org/10.3969/j.issn.1006-6845.2019.15.065

俞静, & 王雁. (2014). 通窍鼻炎颗粒治疗鼻窦炎疗效观察

Clinic Efficacy of Tongqiao Biyan Granules in the Treatment of Sinusitis. *中国药师*(2), 264-265,266. https://d.wanfangdata.com.cn/periodical/ChlQZXJpb2RpY2FsQ0hJTmV3UzIwMjEwNjE2Eg16Z3lzMjAxNDAyMDM3GghjZzR0MWxpMQ%3D%3D

张金庄, 殷璞, 刘宁, 王江宇, 贾云芬, 丁元吉, & 吴垚. (2021). FESS术后加用香菊胶囊与康复新液治疗慢性鼻窦炎伴有鼻息肉的对比研究. *山东大学耳鼻喉眼学报*, *35*(01), 69-76.

张晓明, 王中霞, 赵江涛, & 江燕. (2020). 通窍鼻渊丸在慢性鼻窦炎FESS围手术期的临床疗效观察. *甘肃医药*, *39*(3), 216-217,229. https://d.wanfangdata.com.cn/periodical/ChlQZXJpb2RpY2FsQ0hJTmV3UzIwMjEwNjE2Eg1nc3l5MjAyMDAzMDEwGghjajlqNnY2Nw%3D%3D

迟明泽. (2016). 探讨鼻渊通窍颗粒联合曲安奈德鼻喷雾剂在慢性鼻-鼻窦炎治疗中的临床价值. *世界最新医学信息文摘（连续型电子期刊）*, *16*(A1), 147,152. https://doi.org/10.3969/j.issn.1671-3141.2016.101.111

胡菁, 朱宁, & 张建. (2018). 通窍鼻炎颗粒对慢性鼻窦炎伴鼻息肉远期复发的影响及其机制研究

Effect of Tongqiao Biyan Granule on Long-term Recurrence of Chronic Sinusitis with Nasal Polyps and Its Mechanism. *中国中西医结合耳鼻咽喉科杂志*, *26*(6), 431-435. https://doi.org/10.16542/j.cnki.issn.1007-4856.2018.06.008

侯慎英. (2021). 慢性鼻窦炎鼻息肉鼻窦内窥镜手术联合中药治疗的价值. *中国农村卫生*, *13*(3), 40-41. https://doi.org/10.3969/j.issn.1674-361X.2021.03.019

**5. not about only herbal medicine (n = 23)**

Peng, Q., Qin, G., Hou, T., Liang, Z., & Zhou, W. (2015). [Short-term efficacy observation on Chinese traditional medicine used after functional endoscopic sinus surgery for chronic sinusitis]. *Lin Chung Er Bi Yan Hou Tou Jing Wai Ke Za Zhi*, *29*(12), 1064-1066, 1070.

唐涵芬. (2019). 对接受手术后的慢性鼻窦炎合并鼻息肉患者进行中西医结合治疗的效果评价. *当代医药论丛*, *17*(21), 195-196. https://doi.org/10.3969/j.issn.2095-7629.2019.21.148

梁承志, 黄彦, 李艺, 莫绍毅, 高阳, 陈潇, & 张勉. (2014). “培土生金法”在慢性鼻窦炎鼻内镜术后应用临床研究

Clinical Curative Effect of Invigorating Spleen to Benefit Lung on Chronic Sinusitis after Endoscopic Sinus Surgery. *山西中医*, *30*(2), 16-18. https://d.wanfangdata.com.cn/periodical/ChlQZXJpb2RpY2FsQ0hJTmV3UzIwMjEwNjE2Eg1zeHp5MjAxNDAyMDA4GghsdG1ybzhiNg%3D%3D

黎力, & 许倩. (2018). 鼻炎康汤治疗胆腑郁热型慢性鼻-鼻窦炎78例疗效分析. *饮食保健*, *5*(12), 88-89. https://doi.org/10.3969/j.issn.2095-8439.2018.12.107

刘翠英. (2010). 四联疗法治疗小儿副鼻窦炎疗效观察. *中国民族民间医药*, *19*(16), 128. https://doi.org/10.3969/j.issn.1007-8517.2010.16.119

李静波, 王俊杰, 王慧敏, 陈文明, & 蔡纪堂. (2019). 慢性鼻-鼻窦炎伴鼻息肉围手术期中医药替代激素治疗的疗效观察. *中药材*, *42*(03), 676-679.

李静波, 王俊杰, & 蔡纪堂. (2018). 温阳化湿法治疗慢性鼻-鼻窦炎伴鼻息肉疗效观察

Curative Effect Observation on Wenyang Huashi Method in Treatment of Chronic Rhinosinusitis with Nasal Polyps. *中医学报*, *33*(9), 1782-1785. https://doi.org/10.16368/j.issn.1674-8999.2018.09.422

文凤妮, 张继平, 陈舒华, & 李影雪. (2008). 鼻嗅通治疗儿童慢性鼻窦炎60例临床观察

Effect of Bixiutong for Children Chronic Sinusitis: An Observation of 60 Cases. *新中医*, *40*(4), 35-36. https://doi.org/10.3969/j.issn.0256-7415.2008.04.020

闫福龙. (2018). 中西医结合治疗鼻窦炎临床分析. *中国中医药现代远程教育*, *16*(05), 65-67.

王涛, & 黄雪琨. (2012). 鼻嗅通在儿童慢性鼻窦炎治疗中的作用. *医药前沿*, *2*(14), 87-88. https://doi.org/10.3969/j.issn.2095-1752.2012.14.078

王洪锋, & 翁柏岳. (2013). 香菊胶囊辅助治疗对慢性鼻窦炎鼻息肉患者鼻内镜术后的影响. *辽宁中医杂志*, *40*(11), 2261-2262.

魏绍忠. (2009). 中西结合治疗上颌窦炎. *中国伤残医学*, *17*(6), 158-158. https://doi.org/10.3969/j.issn.1673-6567.2009.06.154

张玲. (2018). 中西医结合护理对慢性鼻窦炎患者鼻内镜术后恢复的影响. *医学理论与实践*, *31*(24), 3774-3776.

张秀强, & 沈志森. (2015). 疏风通窍汤联合鼻内镜手术治疗小儿慢性鼻窦炎鼻息肉疗效观察. *新中医*, *47*(6), 191-192. https://doi.org/10.13457/j.cnki.jncm.2015.06.092

张伟, 鲁文军, 赵绪珍, & 许生聚. (2001). 中药鼻窦置换治疗小儿慢性鼻窦炎67例疗效观察. *新中医*, *33*(7), 20-20. https://doi.org/10.3969/j.issn.0256-7415.2001.07.011

张恩琴, & 林丹. (2018). 中西医结合治疗慢性鼻-鼻窦炎的临床效果

Clinical effect of integrated traditional Chinese and western medicine in the treatment of chronic rhinosinusitis. *临床医学研究与实践*, *3*(9), 115-116. https://doi.org/10.19347/j.cnki.2096-1413.201809056

齐银辉. (2012). *鼻窍整体疏通疗法治疗慢性鼻—鼻窦炎的临床研究* [硕士, 成都中医药大学]. https://d.wanfangdata.com.cn/thesis/ChJUaGVzaXNOZXdTMjAyMTA1MTkSCFkyNTE5OTA5GghsdG1ybzhiNg%3D%3D

朱丽芳. (2017). 鼻内镜规范治疗配合中药内服对慢性鼻窦炎鼻息肉的临床疗效. *临床研究*, *25*(8), 160-161. https://doi.org/10.3969/j.issn.1004-8650.2017.08.096

彭清华, 覃冠锻, & 侯涛. (2016). 托里排脓法对鼻窦炎患者功能性内镜鼻窦手术后鼻窦腔黏膜恢复的影响. *广西医学*, *38*(01), 108-109+112.

彭清华, 覃冠锻, 侯涛, 梁志成, & 周文瑾. (2015). 慢性鼻窦炎术后辅助中医治疗的近期疗效观察

Short-term efficacy observation on chinese traditional medicine used after functional endoscopic sinus surgery for chronic sinusitis. *临床耳鼻咽喉头颈外科杂志*, *29*(12), 1064-1066,1070. https://doi.org/10.13201/j.issn.1001-1781.2015.12.003

何胜恬. (2006). 中药加鼻病雾化剂治疗慢性鼻窦炎临床观察. *中医药学刊*, *24*(6), 1169-1170. https://doi.org/10.3969/j.issn.1673-7717.2006.06.109

叶上珠. (2017). 慢性鼻窦炎中西医结合治疗的疗效和生活质量的临床研究

Clinical Study on the Curative Effect and Quality of Life of Chronic Sinusitis Treated with Integrated Traditional Chinese and Western Medicine. *中国医学创新*, *14*(2), 75-78. https://doi.org/10.3969/j.issn.1674-4985.2017.02.019

叶玉清. (2019). 综合护理干预对慢性鼻窦炎患者鼻内窥镜术后疼痛及心理健康状况的影响. *河南医学研究*, *28*(18), 3447-3449. https://doi.org/10.3969/j.issn.1004-437X.2019.18.104

**6. using EATM intervention in control group (n = 26)**

强建华. (2010). 鼻渊合剂治疗鼻窦炎55例临床观察. *河北中医*, *32*(10), 1483. https://doi.org/10.3969/j.issn.1002-2619.2010.10.021

单金春, & 刘万成. (2020). 通窍理肺汤治疗感冒并发慢性鼻窦炎35例研析讨论. *健康之友*(16), 274. https://d.wanfangdata.com.cn/periodical/ChlQZXJpb2RpY2FsQ0hJTmV3UzIwMjEwNjE2Eg1qa3p5MjAyMDE2NTM3GghjajlqNnY2Nw%3D%3D

仝庆忠. (1998). 自拟鼻渊散治疗小儿慢性鼻窦炎疗效观察. *中国眼耳鼻喉科杂志*(1). http://qikan.cqvip.com/Qikan/Article/Detail?id=689826672199801032

李素娟, & 王智峰. (2013). 加味千金苇茎汤免煎颗粒辅助治疗急性鼻窦炎临床观察. *中国中医急症*, *22*(12), 2130-2131.

李伟萍. (2019). 千金苇茎汤应用于鼻窦炎治疗的有效性研究. *心理月刊*, *14*(03), 159.

李风雷, & 张秋霞. (2009). 芳香通窍丸的制备及临床应用. *中国现代医生*, *47*(11), 112-112,114. https://doi.org/10.3969/j.issn.1673-9701.2009.11.060

潘宁宇. (2019). *自制鼻炎康配方颗粒对慢性鼻—鼻窦炎脾胃湿热证患者的疗效观察* [硕士, 南京中医药大学].

佘秀梅. (2016). 取渊汤加减治疗急性鼻窦炎胆腑郁热证疗效观察. *四川中医*, *34*(02), 170-171.

杨登权, & 胡波. (2014). 竹沥胶囊在真菌性上颌窦炎鼻内镜术后临床应用疗效观察

Clinical application of Juli capsule in fungal maxillay sinusitis after endoscopic sinus surgery. *中医眼耳鼻喉杂志*, *4*(2), 77-78. https://doi.org/10.3969/j.issn.1674-9006.2014.02.007

王丽丽, 潘春林, & 冯善川. (2008). 自制辛鹅鼻安丸治疗慢性鼻窦炎疗效观察. *中医药临床杂志*, *20*(3), 280. https://d.wanfangdata.com.cn/periodical/ChlQZXJpb2RpY2FsQ0hJTmV3UzIwMjEwNjE2EhFhaHp5bGN6ejIwMDgwMzA0MBoIY2c0dDFsaTE%3D

王立. (2020). 加减鼻渊汤联合西医治疗慢性鼻-鼻窦炎的疗效观察. *临床医药文献电子杂志*, *7*(47), 157,162. https://d.wanfangdata.com.cn/periodical/ChlQZXJpb2RpY2FsQ0hJTmV3UzIwMjEwNjE2EhFsY3l5ZHp6ejIwMjA0NzExMBoIY2o5ajZ2Njc%3D

牛玉洁. (2009). *鼻后滴漏综合征的中医治疗及与红细胞免疫的相关性研究* [硕士, 福建中医药大学]. https://d.wanfangdata.com.cn/thesis/ChJUaGVzaXNOZXdTMjAyMTA1MTkSCFkxNTU5Njc0GghsdG1ybzhiNg%3D%3D

韦志武. (2011). 中西医结合治疗急性鼻-鼻窦炎

Acute Sinusitis Treated With Integarted Chinese And Western Medicine. *中外健康文摘*, *8*(29), 78-79. https://doi.org/10.3969/j.issn.1672-5085.2011.29.061

殷志君, 王讯, & 王丽华. (2018). 银花通窍鼻炎汤治疗慢性鼻窦炎的应用效果分析. *家庭医药*(7), 50-51. https://doi.org/10.3969/j.issn.1671-4954.2018.07.055

任永星. (2020). 经鼻内镜手术联合鼻渊通窍颗粒治疗慢性鼻窦炎伴鼻息肉的疗效及复发率观察. *医药前沿*, *10*(36), 152-154. https://d.wanfangdata.com.cn/periodical/ChlQZXJpb2RpY2FsQ0hJTmV3UzIwMjEwNjE2Eg55aXlxeTIwMjAzNjA5MBoIY2o5ajZ2Njc%3D

任应化. (2016). 清渊汤联合西药常规治疗儿童慢性鼻窦炎91例. *中医研究*, *29*(6), 22-24. https://doi.org/10.3969/j.issn.1001-6910.2016.06.11

张群. (2004). 甘露消毒丹治疗急性鼻窦炎临床观察. *湖北中医杂志*, *26*(8), 36-36. https://doi.org/10.3969/j.issn.1000-0704.2004.08.027

张群慧. (2015a). 玉屏风对慢性鼻-鼻窦炎鼻内镜术后炎症因子与免疫指标的影响. *深圳中西医结合杂志*, *25*(16), 27-29. https://doi.org/10.16458/j.cnki.1007-0893.2015.16.013

张群慧. (2015b). 玉屏风散对慢性鼻-鼻窦炎鼻内镜术后炎症因子与免疫指标的影响. *深圳中西医结合杂志*, *25*(16), 27-29.

张淑兰. (2004). 鼻渊舒口服液治疗小儿慢性鼻窦炎的疗效观察附:120例病例报告

Children Chronic Sinusitis Treated with Bi-yuan-shu Oral Liquid：120 Cases Report Attachment. *成都中医药大学学报*, *27*(4), 21-22. https://doi.org/10.3969/j.issn.1004-0668.2004.04.010

张杨, & 郭洁. (2018). 中西药合用治疗慢性鼻窦炎胆腑郁热证疗效观察. *实用中医药杂志*, *34*(06), 676-677.

丁会军. (2000). 针药并施治疗副鼻窦炎疗效观察. *北京针灸骨伤学院学报*, *0*(1). http://qikan.cqvip.com/Qikan/Article/Detail?id=4000706212

赵竞一, 艾建伟, 王雅琴, 王曼, 盖建青, & 王俊阁. (2020). 温阳通窍汤加减对慢性鼻窦炎鼻内镜术后患者鼻黏膜重塑的影响

Effects of Modified Wenyang Tongqiao Decoction（温阳通窍汤）on Mucosa Remodeling of Chronic Rhinosinusitis Patients After Endoscopic Surgery. *中医杂志*, *61*(14), 1247-1252. https://doi.org/10.13288/j.11-2166/r.2020.14.013

周美玲. (2016). *温肺止流丹加减治疗肺气虚寒型慢性鼻-鼻窦炎(Ⅱ型Ⅱ期)鼻内镜术后的疗效观察* [硕士, 福建中医药大学]. https://d.wanfangdata.com.cn/thesis/ChJUaGVzaXNOZXdTMjAyMTA1MTkSCFkzMDYyMjE1Ggg0bjR0d3czdg%3D%3D

陈长武, 钟春明, & 冯建华. (2013). 中西医结合治疗慢性鼻-鼻窦炎FESS术后44例疗效观察. *中医药导报*, *19*(11), 120-121.

何珍, 张滟, & 韩宇. (2018). 鼻窦炎鼻内窥镜围手术期中西医结合治疗的临床疗效分析. *世界中西医结合杂志*, *13*(06), 806-808+833.

**7. only abstract available without raw data (n = 6)**

郭赛, 彭桂原, 向凤, 黄小安, 林山珊, 钱湃梓, 李林蔓, & 黄桂烂. (2019). 皂角刺颗粒治疗慢性鼻窦炎的临床研究. 中华中医药学会耳鼻喉科分会第二十五次学术年会暨世界中联耳鼻喉口腔科专业委员会第十一次学术年会, 中国山东济南.

寻满湘, & 滕磊. (2017). 五味石膏汤在慢性鼻-鼻窦炎围手术期的临床应用. 中华中医药学会耳鼻喉科分会第二十三次学术年会世界中联耳鼻喉口腔科专业委员会第九次学术年会, 中国江苏张家港.

王中霞, 王辉, & 江燕. (2014). *鼻渊丸对慢性鼻-鼻窦炎术后鼻黏膜恢复的临床研究* 2014第四届全国耳鼻咽喉科医师大会论文集, 北京. https://d.wanfangdata.com.cn/conference/ChZDb25mZXJlbmNlTmV3UzIwMjEwNTIxEgc4OTg1ODkyGghjajlqNnY2Nw%3D%3D

张校科, 李建明, 李灵芝, 赵俊义, & 张馨. (2001). *鼻炎一闻灵治疗慢性副鼻窦炎200例疗效观察* 全国中药研究与开发学术研讨会论文摘要集, 中国昆明. https://d.wanfangdata.com.cn/conference/ChZDb25mZXJlbmNlTmV3UzIwMjEwNTIxEg5IWTAwMDAwMjA1NzY1NBoING40dHd3M3Y%3D

詹益斯, 吕剑霆, 刘红, 黄雪琨, 陈靖, 黄亚非, 陶玲, & 文博. (2000). *"拂手治鼻散"治疗学龄儿童慢性化脓性鼻窦炎165例临床疗效观察(摘要)* 第三届第二次全国中西医结合耳鼻咽喉科学术大会论文汇编, 中国浙江宁波. https://d.wanfangdata.com.cn/conference/ChZDb25mZXJlbmNlTmV3UzIwMjEwNTIxEg5IWTAwMDAwMjAyMDU2NxoING40dHd3M3Y%3D

胡启煜. (2015). *千金苇茎汤加减治疗急性鼻窦炎临床疗效观察* 世界中联耳鼻喉口腔专业委员会换届大会暨第七次学术年会、中华中医药学会耳鼻喉分会第二十一次耳鼻喉科学术年会暨辽宁省中医及中西医结合耳鼻咽喉科学术会议论文集, 沈阳. <https://d.wanfangdata.com.cn/conference/ChZDb25mZXJlbmNlTmV3UzIwMjEwNTIxEgc4ODI4Mzg3GghjZzR0MWxpMQ%3D%3D>

**8. duplicate and inappropriate data (n = 2)**

蔺林, 陈峥, 戴飞, 魏瑾瑾, & 汤欣玥. (2019). 连花清瘟颗粒对慢性鼻-鼻窦炎不伴鼻息肉 的治疗作用研究

The efficacy of Lianhuaqingwen for chronic rhinosinusitis without nasal polyps. *中国中西医结合耳鼻咽喉科杂志*, *27*(2), 112-116,146. https://doi.org/10.16542/j.cnki.issn.1007-4856.2019.02.011

熊子云. (2019). 不同中医辨证分型慢性鼻-鼻窦炎患者鼻内窥镜术后中医药治疗效果分析. *检验医学与临床*, *16*(12), 1732-1734.
