# Supplementary material for: Herbal medicine for the treatment of chronic rhinosinusitis: A systematic review and meta-analysis
Source: Front Pharmacol. 2022 Jul 18;13:908941. doi: 10.3389/fphar.2022.908941 (PMC9341451; doi:10.3389/fphar.2022.908941)
Supplement: Supplementary file 4 [file Table3.DOCX]

Supplement 3. General characteristics of the included studies

| **Study ID** | **Sample size (TG:CG)** | **Mean age (range) (yr)** | **CRS disease period (range)** | **Population** | **Pattern identification** | **TG** | **CG** | **Outcome measures** |
| --- | --- | --- | --- | --- | --- | --- | --- | --- |
| Cao 2016 | 60(30:30) | 6.7 ± 1.1 (3~10) | 3.1 ± 0.7yr (5mo~4yr) | CRS | NR | HM + CG | Azithromycin dry suspension, Loratadine dispersible tablets | **1. TER** |
| Chai 2018 | 120(60:60) | TG: 30.1 ± 8.1 (21~59) CG: 29.6 ± 7.6 (18~56) | NR | CRS | NR | HM + CG | Antibiotics and steroids, Cleaning the surgical cavity once every two weeks | **1. TER** 2. Sign disappearance time (wk) |
| Chen 2005 | 60(30:30) | TG: 37.5 (25~50) CG: 38.5 (28~49) | TG: 8yr (1~16yr) CG: 5yr (1~10yr) | CRS | NR | HM + CG | Chlorephedrine nasal drops, Gentamicin and flumethasone | **1. TER** |
| Chen 2016 | 276(138:138) | TG: 8.09 ± 2.13 (4~12) CG: 7.65 ± 1.98 (4~11) | TG: 16.97 ± 6.38mo (4~35mo) CG: 17.23 ± 6.25mo (3~37mo) | CRS | NR | HM + CG | Amoxicillin and clavulanate potassium dry suspension, Ambroxol granules | **1. TER 2. MTR (mm/min) 3. MCC (%)** 4. IL-2 (ng/L) 5. IL-6 (g/L) 6. IL-10 (pg/mL) 7. IFN-γ (kU/L) **8. TNF-α (ng/mL)** 9. TNF-β (ng/L) **10. CRP (mg/L)** |
| Chen 2017 | 80(40:40) | TG: 18.99 ± 3.14 (10~30) CG: 18.21 ± 3.21 (11~29) | TG: 5.24 ± 0.71mo CG: 5.11 ± 0.87mo | CRS | NR | HM + CG | Roxithromycin capsules | **1. TER** **2. Lund-Mackay CT score (0-24)** |
| Chen 2019a | 121(60:61) | TG: 5.32 ± 2.46 (4~10) CG: 5.78 ± 1.57 (4~11) | TG: 1.03 ± 0.59yr (6mo~2yr) CG: 1.05 ± 0.47yr (5mo~2yr) | CRS type I without nasal polyps | NR | HM + CG | Clarithromycin tablets | **1. TER 2. VAS (0-40)** **3. Lund-Kennedy endoscopic score (0-20) 4. Lund-Mackay CT score (0-24)** 5. IL-6 (ng/L) 6. IL-2 (ng/L) **7. MTR (mm/min) 8. MCC (%)** |
| Chen 2019b | 76(38:38) | TG: 7.33 ± 3.06 (3~14) CG: 7.24 ± 3.15 (3~12) | TG: 12.52 ± 4.03mo (3mo~3yr) CG: 12.47 ± 4.25mo (3mo~2.5yr) | CRS without nasal polyps | wind-heat invading the lung meridian | HM + CG | Cefixime capsules | **1. TER 2. Lund-Kennedy endoscopic score (0-20)** 3. TCM symptom score 4. IL-25 (pg/mL) 5. HMGB1 (μg/L) 6. ECP (ng/L) |
| Dai 2013 | 72(37:35) | 11.35 ± 3.23 (8~14) | 3 ± 0.9yr (2~4yr) | Perioperative period of children with CRS with nasal polyps | NR | HM + CG | Antibiotic cefaclor and corticosteroid budesonide nasal spray, Cleaning the surgical cavity twice a day with antibiotics or normal saline | **1. TER** 2. Symptom score |
| Ding 2012 | 100(50:50) | TG: 8.5 (6~12) CG: 8.9 (5~12) | TG: 3.5yr (0.3~6.5yr) CG: 3.8yr (0.4~7yr) | CRS | gallbladder meridian heat depression | HM + CG | Cefixime, Mometasone furoate nasal spray | **1. TER** |
| Du 2016 | 110(55:55) | TG: 35.36 ± 5.71 (18~62) CG: 36.17 ± 6.03 (19~64) | NR | CRS after FESS | NR | HM + CG | Intranasal corticosteroids, antibiotics, nasal irrigation with 9% sodium chloride | 1. Symptom disappearance time (d) |
| Fan 2020 | 50(25:25) | TG: 36.0 ± 2.6 CG: 35.0 ± 2.3 | TG: 13.0 ± 1.5yr CG: 12.5 ± 1.5yr | CRS | - dampness-heat - deficiency cold pattern | HM + CG | Cephalexin capsules or azithromycin tablets, Fluticasone propionate nasal spray | **1. TER** |
| Fu 2020 | 104(52:52) | TG: 36.78 ± 3.52 (29~51) CG: 39.12 ± 3.67 (28~53) | TG: 7.23 ± 3.25yr (5~11yr) CG: 6.27 ± 3.58yr (3~10yr) | CRS | NR | HM + FESS | FESS, Beclomethasone propionate nasal spray | **1. MTR (mm/min) 2. Lund-Mackay CT score (0-24) 3. Lund-Kennedy endoscopic score (0-20) 4. hs-CRP (pg/L) 5. TNF-a (pg/mL)** 6. IL-6 (pg/mL) **7. Serum total IgE (kU/L)** |
| Gou 2020 | 96(48:48) | TG: 42.56 ± 7.89 (18~65) CG: 42.78 ± 7.95 (18~64) | TG: 5.23 ± 0.53yr (4~7yr) CG: NR | CRS | NR | HM + FESS | FESS, Nasal irrigation, budesonide nasal spray | 1. Symptom score **2. Lund-Kennedy endoscopic score 3. Lund-Mackay CT score** 4. IgG (g/L) 5. IgM (g/L) **6. TER** |
| He 2020 | 105(53:52) | TG: 41.11 ± 7.16 CG: 40.85 ± 6.29 | TG: 2.31 ± 0.88yr CG: 2.26 ± 0.74yr | CRS with nasal polyp | NR | HM + CG | FESS, Nasal irrigation with sodium chloride injection | **1. TER 2. MTR (mm/min)** 3. Distribution symptoms 4. Sinus mucosal epithelialization time (wk) **5. Recurrence rate (after 6mo)** |
| Hou 2018 | 72(36:36) | TG: 38.12 ± 4.14 (25~71) CG: 38.17 ± 4.11 (25~72) | TG: 6.41 ± 1.21yr (1~13yr) CG: 6.44 ± 1.24yr (1~13yr) | CRS | NR | HM + CG | Amoxicillin, triamcinolone acetonide nasal spray | **1. TER** 2. Symptom disapperance time (d) 3. Symptom score 4. Quality of life **5. CRP (mg/L)** |
| Hu 2019 | 82(41:41) | TG: 28.6 ± 5.0 (22~48) CG: 28.8 ± 5.5 (20~50) | TG: 6.2 ± 2.7yr (2~13yr) CG: 6.3 ± 3.0yr (3~15yr) | CRS | NR | HM + CG | Amoxicillin-clavulanate potassium tablets | **1. TER** 2. Symptom score |
| Huang 2015 | 66(33:33) | TG: 38.8 ± 4.5 (9~62) CG: 39.7 ± 4.2 (8~62) | TG: 4.0 ± 0.9yr (1mo~8yr) CG: 4.1 ± 0.8yr (1mo~9yr) | CRS | NR | HM + CG | Clarithromycin sustained-release tablets | **1. TER** 2. Epithelialization time (d) |
| Huang 2020 | 80(40:40) | TG: 42.24 ± 2.12 (20~57) CG: 42.89 ± 2.56 (21~57) | NR | Refractory CRS | NR | HM + CG | Glucocorticoid, Antibiotics | **1. TER** 2. Symptom score **3. CRP (mg/L)** 4. IL-6 (pg/mL) 5. IL-8 (pg/mL) 6. Symptom disappearance time |
| Jiang 2012 | 53(26:27) | TG: 52.9 ± 2.8 (18~86) CG: 53.7 ± 2.2 (27~78) | NR | CRS without nasal polyps | NR | HM + Erythromycin placebo | Erythromycin + HM placebo | **1. SNOT-20 2. Lund-Kennedy endoscopic score** 3. Bacterial culture rate 4. The decrease in the ratio of the saccharin transit time |
| Jiang 2021a | 94(48:46) | TG: 6.83 ± 1.04 (2~13) CG: 6.92 ± 1.21 (3~15) | TG: 2.55 ± 0.27yr (0.2~4yr) CG: 2.83 ± 0.36yr (0.5~7yr) | CRS | wind-heat invading the lung meridian | HM | Roxithromycin | **1. TER** 2. VC-max (L) 3. FEV1 (L) 4. FEV1/FVC (%) 5. MMEF (L/s) |
| Jiang 2021b | 99(45:44) | TG: 23.71 ± 4.51 CG: 23.56 ± 4.37 | TG: 2.13 ± 0.38yr CG: 2.09 ± 0.42yr | CRS caused by mycoplasma pneumonia infection | wind-heat invading the lung meridian | HM + CG | Azithromycin enteric-coated tablets | **1. TER** 2. TCM symptom score 3. IL-2 4. IL-4 5. IL-10 **6. Recurrence rate** |
| Li 2015 | 78(39:39) | TG: 7.55 ± 2.86 CG: 7.34 ± 3.03 | ≧ 3 mo | CRS | NR | HM + CG | Cefprozil granules, Mometasone furoate nasal spray | **1. TER** |
| Li 2018a | 112(56:56) | TG: 41.78 ± 11.22 (19~65) CG: 42.51 ± 10.49 (18~64) | TG: 8.25 ± 4.05mo (1~16mo) CG: 8.18 ± 3.65mo (1~15mo) | CRS | NR | HM + CG | Clarithromycin tablets | **1. TER 2. VAS 3. Lund-Mackay CT score 4. Lund-Kennedy endoscopic score** |
| Li 2018b | 66(33:33) | TG: 31.3 ± 3.7 (20~44) CG: 30.7 ± 3.8 (19~46) | NR | CRS | NR | HM + CG | Amoxicillin capsules | **1. TER** 2. Symptom score **3. VAS** |
| Li 2021 | 60(30:30) | TG: 42.40 ± 7.97 (20~60) CG: 41.36 ± 8.01 (20~60) | TG: 9.42 ± 3.32mo (1~16mo) CG: 10.39 ± 2.28mo (1~16mo) | CRS | NR | HM + CG | Clarithromycin tablets | 1. Symptom score **2. TNF-α (ng/mL) 3. hs-CRP (mg/L)** 4. IL-1β (pg/mL) 5. IL-5 (pg/mL) |
| Liang 2004 | 340(170:170) | TG: 38.96 ± 14.02 CG: 38.70 ± 13.89 | TG: 63.68d CG: 82.19d | CRS after FESS | NR | HM + CG | Quinolone antibiotics, Steroid (Fushuliang nasal spray) | **1. TER** 2. Disappearance rate of main symptoms 3. Symptom disapperace time (d) |
| Liao 2020 | 120(60:60) | TG: 40.8 ± 2.6 (16~62) CG: 41.2 ± 2.4 (16~64) | TG: 2.6 ± 0.5yr (10mo~5yr) CG: 2.4 ± 0.6yr (1~5yr) | CRS | gallbladder heat depression | HM + CG | Clarithromycin, Gentamicin sulfate injection, Dexamethasone sodium phosphate injection, Nasal irrigation | **1. TER** **2. IgE (IU/mL)** 3. IL-6 (ng/L) **4. MTR (mm/min)** |
| Lin 2010 | 103(51:52) | TG: 33.85 ± 11.60 (18~60) CG: 29.65 ± 11.26 (18~57) | TG: 6.36 ± 3.58yr  CG: 6.57 ± 2.49yr | CRS after FESS | - retained heat invading the lung meridian - dampness-heat in the spleen and stomach, liver and gallbladder - dual deficiency of the lung-spleen | HM | None | **1. TER** 2. Postoperative cleaning time 3. Epithelialization time (d) 4. Vesicle appearance time (d) 5. Vesicle duration time (d) |
| Lin 2013 | 64(34:30) | TG: 32.3 (18~55) CG: 31.6 (18~55) | TG: 6.12 ± 2.32yr CG: 6.23 ± 2.54yr | CRS | NR | HM + CG | Roxithromycin, Mucosultan, Nasal spray | **1. TER 2. VAS** |
| Lin 2017 | 100(50:50) | TG: 7.6 ± 1.5 (4~11) CG: 7.4 ± 1.9 (4~10) | TG: 15.6 ± 1.5mo (3~37mo) CG: 15.3 ± 1.7mo (3~36mo) | CRS | NR | HM + CG | Clarithromycin | **1. TER** **2. Lund-Mackay CT score (0-24) 3. Lund-Kennedy endoscopic score (0-20) 4. VAS (0-10)** 5. Improvement of main symptoms |
| Lin 2020 | 144(72:72)→ 140(70:70) | TG: median 51 (25~71) CG: median 54 (18~75) | NR | CRS without nasal polyps | NR | HM | Placebo | **1. VAS 2. SNOT-22** 3. rhinomanometer 4. nasal NO concentration (ppb) 5. Histochemical analysis (inflammatory cell counts) 6. CD4+ (%) 7. CD8+ (%) 8. IL-4 (pg/mL) 9. IL-5 (pg/mL) 10. ECP (ng/mL) 11. IFN-r (pg/mL) 12. neutrophil myeloperoxidase (ng/mL) **13. TNF-a (pg/mL)** |
| Liu 2012 | 48(24:24) | TG: 28.83 (18~47) CG: 29.75 (18~54) | TG: 5.71yr (4mo~21yr) CG: 7.812yr (6mo~26yr) | CRS without nasal polyps | NR | HM + Clarithromycin placebo | Clarithromycin tablets + HM placebo | **1. VAS (0-10) 2. Lund-Mackay CT score (0-24)** |
| Liu 2017 | 70(35:35) | TG: 65.91 ± 6.99 (61~67) CG: 66.57 ± 8.72 (63~69) | TG: 2.31 ± 0.43yr (9mo~3.5yr) CG: 2.36 ± 0.39yr (10mo~4yr) | CRS with nasal polyp | NR | HM + CG | FESS, Nasal irrigation, 3% sodium chloride, Fluticasone propionate spray | **1. VAS (0-10)   (nasal obstruction, olfactory dysfunction,   rhinorrhea, facial pain) 2. Lund-Kennedy endoscopic score  3. Lund-Mackay CT score  4. Recurrence rate** 5. CD3+ 6. CD4+  7. CD8+ |
| Liu 2018 | 80(40:40) | TG: 38.95 ± 3.96 (27~61) CG: 38.98 ± 4.12 (25~60) | TG: 5.05 ± 1.13yr (1~7yr) CG: 5.03 ± 1.11yr (1~8yr) | CRS without nasal polyps | gallbladder heat depression | HM + CG | Clarithromycin tablets, Nasal irrigation | **1. TER** 2. IL-6 (pg/mL) **3. TNF-α (pg/mL)** **4. CRP (mg/L)** **5. IgE (IU/mL) 6. MTR (mm/min) 7. SNOT-20** |
| Liu 2019 | 60(30:30) | TG: 35.50 ± 14.29 (18~64) CG: 35.10 ± 13.56 (18~63) | TG: 9.26 ± 4.02mo (3~18mo) CG: 9.30 ± 4.20mo (3~18mo) | CRS without nasal polyps | lung qi deficiency cold | HM + Mometasone furoate nasal spray | Roxithromycin tablets, Mometasone furoate nasal spray | **1. VAS (0-40) 2. Lund-Mackay CT score (0-24) 3. Lund-Kennedy endoscopic score (0-8) 4. TER** |
| Liu 2020 | 88(44:44) | TG: 32.54 ± 3.41 (25~59) CG: 32.43 ± 3.46 (26~60) | TG: 13.54 ± 2.15yr (6~20yr) CG: 13.37 ± 2.32yr (5~20yr) | CRS | - dual deficiency of the lung-spleen - lung qi deficiency cold | HM + CG | Fluticasone propionate, Prednisone | **1. TER** 2. FVC (L) 3. FEV1 (L) 4. FEV1/FVC (%) 5. IL-17 (pg/mL) 6. IL-1β (pg/mL) 7. IFN-γ (pg/mL) **8. TNF-α (pg/mL)** |
| Lu 2011 | 110(55:55) | TG: 35.5 ± 18.5 CG: 36.5 ± 17.8 | NR | CRS | NR | HM | Levofloxacin, Furancortisone nasal drops | **1. TER** |
| Ma 2016 | 96(48:48) | TG: 8.60 ± 2.37 (6~13) CG: 8.70 ± 2.49 (6~12) | NR | CRS | NR | HM + CG | Clarithromycin granules | **1. VAS (0-10) 2. TER** |
| Ma 2020 | 158(79:79) | TG: 34.69 ± 67.08 (20~59) CG: 35.92 ± 6.11 (24~58) | TG: 5.14 ± 2.23yr (5~13yr) CG: 5.06 ± 2.37yr (2~12yr) | CRS | NR | HM + CG | FESS | 1. Symptom score **2. CRP (pg/L)** 3. IL-6 (pg/L) 4. IL-8 (pg/L) 5. CD3+ (%) 6. CD4+ (%) 7. CD8+ (%) 8. CD4+/CD8+ **9. Recurrence rate** |
| Peng 2020 | 68(34:34) | TG: 45.29 ± 2.38 (22~59) CG: 46.71 ± 3.02 (24~62) | NR | CRS with nasal polyp | NR | HM + CG | FESS, Mometasone furoate nasal spray | **1. VAS (0-10) 2. Lund-Kennedy endoscopic score (0-20)** 3. TGF-β1 (ng/L) 4. IL-6 (ng/L) 5. IL-8 (ng/L) 6. T&T standard olfactory test |
| Qian 2019 | 120(60:60) | TG: 9.28 ± 2.43 (6~16) CG: 9.44 ± 2.76 (6~16) | TG: 12.48 ± 5.12mo (3~28mo) CG: 12.51 ± 5.97mo (3~28mo) | CRS | dampness-heat in the liver and gallbladder | HM + CG | Clarithromycin | **1. TER** 2. TCM symptom score 3. SIgA (μ/mL) |
| Shao 2019 | 100(50:50) | TG: 38.84 ± 4.68 (20~60) CG: 37.89 ± 5.98 (18~58) | TG: 4.43 ± 1.95yr (1.3~10.6yr) CG: 4.76 ± 1.79yr (1.4~10.6yr) | CRS | NR | HM + CG | Clarithromycin tablets | **1. VAS 2. Lund-Mackay CT score 3. Lund-Kennedy endoscopic score 4. TER** |
| Shen 2013 | 360(180:180) | TG: 32.6 (21~47) CG: 32.4 (22~46) | 3.5yr (5mo~6yr) | CRS | NR | HM + CG | Antibiotics, Steroid nasal spray | **1. TER 2. Recurrence rate** |
| Shen 2020 | 90(45:45) | TG: 41.17 ± 3.4 CG: 40.20 ± 3.36 | TG: 2.39 ± 0.26yr  CG: 2.43 ± 0.31yr | CRS after surgery | dual deficiency of the lung-spleen | HM + CG | Budesonide nasal spray, Levofloxacin capsules, Normal saline flushing nasal cavity | **1. TER** 2. Symptom disappearance time (d) **3. Lund-Kennedy endoscopic score (0-20)** 4. CT score (3-18)  5. olfactory function score  6. nasal ventilation function **7. MTR (mm/min) 8. MCC (%) 9. Recurrence rate** |
| Song 2021 | 92(46:46) | TG: 44.67 ± 4.59 (25~66) CG: 44.27 ± 4.36 (24~65) | TG: 8.12 ± 2.03yr CG: 8.25 ± 2.12yr | CRS | lung qi deficiency cold | HM + CG | Mometasone furoate nasal spray | **1. TER 2. VAS (0-10) 3. Lund-Mackay CT score 4. Lund-Kennedy endoscopic score 5. SNOT-20** 6. IL-6 (pg/ml) 7. IL-17 (pg/ml) **8. TNF-a (ng/ml)** |
| Sun 2017 | 84(42:42) | TG: 41.6 ± 9.8 (22~64) CG: 42.7 ± 9.4 (20~65) | TG: 21.8 ± 11.9yr (6mo~39yr) CG: 22.1 ± 12.4yr (5mo~40yr) | CRS | NR | HM + CG | Roxithromycin capsules | **1. VAS 2. TER** |
| Tang 2020 | 80(40:40) | TG: 39.5 ± 17.5 (22~57) CG: 40.5 ± 17.5 (23~58) | TG: 6.0 ± 3.0yr (1~9yr)  CG: 5.5 ± 2.5yr (1~8yr) | CRS | dampness-heat in the liver and gallbladder | HM + CG | Mometasone furoate spray, Azithromycin capsule | **1. TER** 2. Nasal mucociliary clearance time (min) **3. SNOT-20  4. Lund-Kennedy endoscopic score (0-24)** 5. TER (TCM syndrome score) |
| Tao 2020 | 214(107:107) | TG: 9.25 ± 2.36 (6~12) CG: 9.63 ± 2.28 (6~11) | TG: 6.27 ± 3.50mo (2~14mo) CG: 6.50 ± 3.45mo (1~15mo) | CRS | NR | HM + CG | Active silver ion antibacterial liquid, Nasal spray | **1. TER 2. SNOT-20** 3. IL-5 (ng/L) 4. IL-8 (ng/L) |
| Wang 2016 | 92(46:46) | TG: 46.3 ± 8.4 (22~67) CG: 45.8 ± 8.5 (23~65) | TG: 3.9 ± 0.8yr (1~7yr) CG: 3.8 ± 0.7yr (1~6yr) | CRS | NR | HM + CG | Triamcinolone acetonide nasal spray | **1. TER** 2. Symptom disapperance time (d) |
| Wang 2017 | 80(40:40) | TG: 42.9 ± 4.5  CG: 43.1 ± 4.2 | TG: 6.3 ± 2.4yr  CG: 6.1 ± 2.2yr | CRS | gallbladder heat depression | HM + CG | FESS, Cephalosporin, Dexamethasone sodium phosphate Injection, Cefradine capsules, Nasal irrigation | **1. VAS (0-10)**  2. Symptom score 3. Nasal mucociliary clearance time (min) **4. MCC (%)** 5. MTR (mm/min) **6. TER** 7. Quality of life (0-10) |
| Wang 2020a | 60(30:30) | TG: 47.0 ± 1.9 (24~63) CG: 46.6 ± 2.5 (24~62) | NR | CRS | NR | HM + CG | Anti-allergic drugs, Nasal irrigation, Mucolysis promoting agents, Decongestants, Azithromycin | **1. TER 2. SNOT-20** 3. IL-6 (ng/L) 4. IL-2 (ng/L) |
| Wang 2020b | 92(46:46) | TG: 36.27 ± 2.17 (20~45) CG: 35.19 ± 2.07 (22~43) | TG: 5.62 ± 0.42yr (1~10yr) CG: 5.37 ± 0.56yr (2~11yr) | CRS | NR | HM + CG | FESS, Antibiotics, Nasal irrigation | **1. TER** |
| Wei 2017 | 120(60:60) | 36.4 ± 4.7 (20~50) | 2.5 ± 1.5yr (0.5~6yr) | CRS | NR | HM + CG | Clarithromycin, Beclomethasone dipropionate nasal aerosol | **1. TER** 2. SF-36 |
| Xia 2019 | 200(100:100)→197(99:98) | TG: 43.12 ± 6.21 (34~59) CG: 43.03 ± 5.78 (33~58) | TG: 26.98 ± 3.61mo (13~44mo) CG: 26.55 ± 3.94mo (11~41mo) | CRS | gallbladder meridian heat depression | HM + Clarithromycin | Flushing nasal cavity with 0.9 sodium chloride injection, Clarithromycin | **1. TER 2. VAS (0-10) 3. Lund-Mackay CT score (0-24) 4. Lund-Kennedy endoscopic score (0-16)** 5. Symptom score of gallbladder meridian stagnation and heat syndrome **6. SNOT-20** |
| Xiang 2017 | 80(40:40) | TG: 5.8 ± 0.3 (3~11) CG: 6.4 ± 0.3 (3~12) | TG: 1.5 ± 0.3yr (2mo~4yr) CG: 1.6 ± 0.1yr (2mo~4yr) | CRS | NR | HM + CG | Clarithromycin | **1. TER 2. VAS (0-40)** **3. Lund-Kennedy endoscopic score (0-20) 4. Lund-Mackay CT score (0-24)** 5. Improvement of the main symptoms (case) |
| Xie 2021 | 130(65:65) | TG: 25.41 ± 5.83 (20~50) CG: 26.23 ± 4.21 (21~49) | TG: 2.15 ± 0.34yr (0.6~4.5yr) CG: 2.23 ± 0.31yr (0.5~4yr) | CRS | NR | HM + CG | Amoxicillin, Clavulanate potassium capsules, Budesonide nasal spray, Nasal cleaning device | **1. TER** 2. Symptom score 3. Changes in nasal cavity flora (aerobic bacteria, fungus etc.) (%) **4. SNOT-20 (F/U 1 year after treatment)** |
| Xin 2010 (3군이나 본 연구에 맞는 그룹 정보만 추출) | 120(60:60)→104(53:51) | TG: 38.70 ± 11.95 CG: 39.55 ± 12.40 | TG: 42.06 ± 36.61mo CG: 37.49 ± 33.11mo | CRS | NR | HM + surgery | Roxithromycin capsules, Gelomyrtol, Surgery | **1. TER 2. Recurrence rate** |
| Xing 2015 | 60(30:30) | TG: 41.5 ± 3.1 (5~80) CG: 40.9 ± 3.2 (5.2~78) | TG: 5.2 ± 1.3yr (8mo~31yr) CG: 4.9 ± 1.6yr (9mo~31.2yr) | CRS | dual deficiency of qi and blood (deficiency cold, dampness-heat) | HM + CG | FESS, Budesonide nasal spray, Nasonex, Beclomethasone dipropionate aqueous nasal spray, Rinsing the nasal cavity with normal saline | **1. TER** |
| Xu 2013 | 128(64:64) | TG: 38.52 ± 3.67 CG: 40.96 ± 3.11 | TG: 3.70 ± 1.19d CG: 3.60 ± 1.22d | CRS | NR | HM + CG | FESS, Antibacterial drugs, Dexamethasone, Mucous excretion agents, Rinsing the nasal cavity regularly | **1. TER** 2. Nasal function recovery time (average cleaning time of the operation cavity, epithelialization time) 3. Postoperative symptoms and signs disappearance (cases) |
| Xu 2019 | 86(43:43) | TG: 38.8 ± 9.4 (21~58) CG: 38.2 ± 9.1 (22~59) | TG: 9.4 ± 3.1mo (2~18mo) CG: 10.1 ± 2.9mo (3~20mo) | CRS | NR | HM + CG | Clarithromycin tablets, Fluticasone propionate nasal spray | **1. TER 2. VAS (0-10)** |
| Yang 2013 | 217(109:108) | TG: 35.69 ± 7.38 (15~65) CG: 35.34 ± 7.91 (16~64) | TG: 4.58 ± 2.87yr (1.5~18.6yr) CG: 4.74 ± 2.72yr (1.5~19.5yr) | CRS after FESS | NR | HM + CG | Conventional treatment after surgery (penicillins, cephalosporins, quinolones, and other antibiotics for 5~10d), Dexamethasone 10mg/d IV after 3d, switch to Prednisone tablets 20mg for 1-2wks | **1. TER** 2. Epithelialization time (wk) 3. Improved sense of smell (case) **4. Recurrence rate** |
| Yang 2016 | 96(48:48) | 27.67 ± 8.43 (14~53) | 57.45 ± 8.97mo (3mo~7yr) | CRS | NR | HM + CG | Moxifloxacin, Cefdinir | **1. TER 2. Recurrence rate** |
| Yang 2018 | 74(40:34) | TG: 28.8 ± 2.6 (16~43) CG: 29.3 ± 2.5 (15~42) | TG: 1.8 ± 0.3yr (4mo~4.2yr) CG: 1.7 ± 0.4yr (3mo~3yr) | CRS | NR | HM + CG | Clarithromycin | **1. TER** 2. Time to disappearance of clinical symptoms |
| Yang 2020 | 90(45:45) | TG: 29.78 ± 8.27 (22~40) CG: 29.56 ± 7.29 (24~42) | TG: 10.35 ± 9.4yr (2~27yr) CG: 10.36 ± 9.42yr (2~28yr) | CRS | NR | HM + CG | Clarithromycin | **1. TER 2. TNF-α (pg/ml)** 3. IL-6 (pg/ml) 4. IL-8 (ng/L) |
| Yao 2019 | 86(43:43) | TG: 8.1 ± 2.2 (6~13) CG: 8.2 ± 2.3 (7~12) | TG: 1.1 ± 0.5yr (1mo~2yr) CG: 1.1 ± 0.6yr (2mo~2yr) | Suppurative CRS without nasal polyp | NR | HM | Cefprozil capsule, 0.5% furosemide nasal drops, Loratadine, Negative pressure exhaust replacement method | **1. TER** 2. TCM symptom score |
| Yun 2019 | 100(50:50) | TG: 42.1 ± 10.6 (18~68) CG: 42.3 ± 10.1 (18~65) | TG: 4.5 ± 1.2yr (1.5~20yr) CG: 4.6 ± 1.1yr (1.5~18yr) | CRS after FESS | NR | HM + CG | After 5d of operation, IV infusion of cefradin, Dexamethasone sodium phosphate injection, 5d later changed to cefradine capsules, Rinsing the nasal cavity daily | **1. TER 2. MTR (mm/min) 3. MCC (%)** 4. Nasal mucus fiber clearance time (min) |
| Zhang 2004 | 278(132:146) | TG: 5~51 CG: 4~54 | TG: 1~20yr CG: 1~20yr | CRS | NR | HM | Antibiotics | **1. TER (6 mo F/U)** |
| Zhang 2015 | 90(45:45) | 46.06 ± 2.31 (22~70) | 4.26 ± 1.05yr (5mo~10yr) | CRS | NR | HM + CG | Penicillin V potassium tablets 0.5 g, Metronidazole 0.4 g, 1% furosemide nasal drops | **1. TER 2. SNOT-20 3. VAS (0-10)** |
| Zhang 2016a | 120(60:60) | TG: 22.8 ± 3.5 (17~68) CG: 22.5 ± 3.2 (15~65) | TG: 3.5 ± 0.8yr (0.4~8yr) CG: 3.2 ± 0.9yr (0.3~8yr) | CRS | NR | HM + CG | Beclomethasone dipropionate nasal aerosol, Clarithromycin | **1. TER** |
| Zhang 2016b | 100(50:50) | TG: 42.7 ± 6.4 (20~58) CG: 42.5 ± 6.5 (21~57) | TG: 5.1 ± 2.0yr (1.4~14.9yr) CG: 5.3 ± 2.1yr (1.7~15.2yr) | CRS after FESS | gallbladder heat depression | HM + CG | Within 5d after operation, cephalosporins or penicillins combined with dexamethasone sodium phosphate injection, 5d later changed to cefradine capsules for 2wks, rinsing the nasal cavity daily. | **1. TER** 2. Clinical symptom score **3. VAS (0-10) 4. MTR (mm/min) 5. MCC (%)** 6 Nasal mucus fiber clearance time (min) 7. Quality of life |
| Zhang 2018 | 60(30:30) | TG: 36.60 ± 10.51 (18~58) CG: 36.53 ± 10.95 (18~60) | TG: 4.0 ± 2.21yr CG: 3.9 ± 2.18yr | CRS without nasal polyps | dual deficiency of the lung-spleen | HM | Roxithromycin sustained release capsules, Mometasone furoate aqueous nasal spray | **1. TER 2. VAS (0-40) 3. Lund-Kennedy endoscopic score (0-20) 4. Lund-Mackay CT score (0-24)** |
| Zhang 2019a | 117(59:58) | TG: 34.3 ± 4.1 (17~69) CG: 33.5 ± 4.3 (15~71) | TG: 5.5 ± 0.8yr (0.5~12yr) CG: 5.3 ± 0.8yr (0.5~11yr) | CRS | NR | HM | Clarithromycin, Budesonide nasal spray | **1. TER 2. SNOT-22 3. VAS (0-10)** |
| Zhang 2019b | 166(83:83)→164(82:82) | TG: 46.5 ± 16.21 CG: 46.72 ± 14.58 | TG: 6.20 ± 3.81yr CG: 6.45 ± 3.64yr | CRS | NR | HM + CG | FESS, Mometasone furoate aqueous nasal spray, Cleaning the surgical cavity regularly | **1. TER  2. VAS (0-40) 3. Lund-Kennedy endoscopic score (0-20) 4. Lund-Mackay CT score (0-24)** |
| Zhang 2020 | 60(30:30) | TG: 44.27 ± 11.04 (25~63) CG: 43.93 ± 10.45 (27~60) | TG: 33.97 ± 13.38mo (10~63mo) CG: 32.40 ± 13.27mo (10~63mo) | CRS with nasal polyps | spleen qi deficiency | HM + CG | FESS, IV infusion of antibiotics for 5d, Eucalyptus soft capsule, Flushing the nasal cavity with 250ml of normal saline, Mometasone furoate nasal spray | **1. TER  2. VAS (0-10) 3. Lund-Kennedy endoscopic score (0-20)** |
| Zhang 2021 | 92(46:46) | TG: 35.72 ± 8.61 (21~68) CG: 36.10 ± 8.47 (23~69) | TG: 3.84 ± 2.51yr (2~9yr) CG: 3.95 ± 2.64yr (3~10yr) | CRS with nasal polyps | dampness-heat in the spleen and stomach | HM + CG | FESS, Penicillin antibiotics, Budesonide nasal spray, Cleaning the surgical cavity regularly | **1. TER** 2. TCM syndrome score 3. TGF-β1 positive cells (%) 4. IL-17 positive cells (%) **5. MTR (mm/min) 6. MCC (%)** 7. Nasal mucus fiber clearance time (min) 8. Operation cavity rehabilitation |
| Zhou 2008 | 80(40:40) | TG: 42.6 (16~73) CG: 41.2 (17~71) | TG: 4.3yr (8mo~16yr) CG: 4.7yr (6mo~18yr) | CRS without nasal polyps | NR | HM | Antibiotics (mainly combined with broad-spectrum antibiotics and metronidazole) | **1. TER (6 mo F/U)** |
| Zhou 2012 | 300(150:150) | TG: 16.52 (6y1mo~30yr) CG: 14.25 (5y6mo~30yr) | TG: 7.36mo CG: 7.23mo | CRS | NR | HM + CG | Amoxicillin, Clavulanate potassium dispersible tablets plus furama nasal drops | **1. TER** 2. CT recovery rate |
| Zhou 2013 | 60(30:30) | TG: 48.2 ± 7.3 CG: 45.9 ± 5.4 | TG: 5.7 ± 4.9yr CG: 5.2 ± 6.3yr | CRS | gallbladder heat depression | HM | placebo HM | **1. TER** 2. TCM syndrome score 3. SF-36  **4. SNOT-20 5. VAS (0-10)** |
| Zhu 2014 | 194(96:98) | TG: 30.3 ± 14.5 (17~63) CG: 29.5 ± 15.3 (16~65) | TG: 3.2 ± 1.5yr (0.5~10yr) CG: 2.9 ± 1.7yr (0.5~8.5yr) | CRS after FESS | NR | HM + CG | IV infusion of sensitive antibiotics for 3-5d after operation, then change to amoxicillin and clavulanate potassium tablets for 4wks, Flushing nasal cavity with 0.9% saline, Gentamicin, Dexamethasone | **1. TER** 2. Symptom disappearance time 3. Main symptom scores **4. Lund-Kennedy endoscopic score (0-20) 5. MTR (mm/min)** |
| Zou 2007 | 72(36:36) | 33 (16~50) | NR | CRS after FESS | NR | HM + CG | Antibiotics, Dexamethasone, Ephedrine hydrochloride and nitrofurazone nasal drops, Compound menthol glycerid, Flixonase aqueous nasal spray | **1. TER** |

CG, control group; CRP, C-reactive protein; CRS, chronic rhinosinusitis; CT, computer tomography; ECP, eosinophil cationic protein; FESS, functional endoscopic sinus surgery; FEV1, forced expiratory volume in one second; FVC, forced vital capacity; HM, herbal medicine; HMGB1, high mobility group box 1; hs-CRP, high sensitivity C-reactive protein; IFN, interferon; IL, interleukin; MCC, mucociliary clearance rate; MMEF, maximal mid-expiratory flow; MTR, mucociliary transport rate; NO, nitric oxide; NR, not reported; SF-36, the 36-item short form survey; SNOT, sino-nasal outcome test; TCM, traditional Chinese medicine; TER, total effective rate; TG, treatment group; TGF, transforming growth factor' TNF, tumor necrosis factor; VAS, visual analogue scale; VC, vital capacity.
