# Supplementary material for: Herbal medicine for the treatment of chronic rhinosinusitis: A systematic review and meta-analysis
Source: Front Pharmacol. 2022 Jul 18;13:908941. doi: 10.3389/fphar.2022.908941 (PMC9341451; doi:10.3389/fphar.2022.908941)
Supplement: Supplementary file 5 [file Table4.DOCX]

Supplement 4. Details of herbal medicine used

| **Study ID** | **Herbal medicine formulation** | **Dosage form** | **Main herb components (per day)** | **Additional components (per day)** | **Administration period** | **Follow-up period** | **Quality control**  **Reported / Chemical analysis reported**  **(Y/N)** |
| --- | --- | --- | --- | --- | --- | --- | --- |
| Cao 2016 | No name | Decoction | Magnolia denudata Desr. [Magnoliaceae; Magnoliae Flos] , Ligusticum chuanxiong Hort [Apiaceae; Ligustici Rhizoma], Fructus liquidambaris [Liquidambar formosana Hance.], Akebia quinata Decne. [Lardizabalaceae; Akebiae Caulis], Angelica dahurica Benth. et Hooker f. [Apiaceae; Angelicae Dahuricae Radix] 10 g, Mentha arvensis var. piperascens Makinv. [Lamiaceae; Menthae Herba], Gardenia jasminoides var. grandiflora (Lour.) Nakai [Rubiaceae; Gardeniae Fructus], Bupleurum falcatum Linne [Apiaceae; Bupleuri Radix], Gentiana scabra Bunge [Gentianaceae; Gentianae Radix], Poria cocos (Schw.) Wolf [Polyporaceae; Poria(Hoelen)], Platycodon grandiflorum (Jacq.) A. DC. [Campanulaceae; Platycodi Radix], Xanthium strumarium L. [Asteraceae; Xanthii Fructus] 6 g, Astragalus membranaceus Bunge [Leguminosae; Astragali Radix] 24 g | None | 1mo | 2mo | N / N |
| Chai 2018 | No name | Decoction | Nelumbo nucifera Gaertner [Nymphaceae; Nelumbinis Semen], Coix lachryma-jobi var. ma-yeun (Roman.) Stapf [Gramineae; Coicis Semen], Amomum villosum Lour. [Zingiberaceae; Amomi Fuctus], Platycodon grandiflorum (Jacq.) A. DC. [Campanulaceae; Platycodi Radix], Dolichos lablab L. [Leguminosae; Dolichoris Semen], Poria cocos (Schw.) Wolf [Polyporaceae; Poria(Hoelen)], Panax ginseng C. A. Mey. [Araliaceae; Ginseng Radix], Atractylodes macrocepha-la Koidz [Asteraceae; Atractylodis Rhizoma Alba], Dioscorea batatas Decne. [Dioscoreaceae; Dioscoreae Rhizoma], Glycyrrhiza uralensis Fisch. [Leguminosae; Glycyrrhizae Radix] | None | 2mo | None | N / N |
| Chen 2005 | Gegen decoction | Decoction | Pueraria thunbergiana Benth. [Leguminosae; Puerariae Radix] 12 g, Ligusticum chuanxiong Hort [Apiaceae; Ligustici Rhizoma] 10 g, Magnolia denudata Desr. [Magnoliaceae; Magnoliae Flos], Cinnamomum cassia Blume [Lauraceae; Cinnamomi Ramulus], Paeonia albiflora Pallas var. trichocarpa Bunge [Paeoniaceae; Paeoniae Radix Alba], Glycyrrhiza uralensis Fisch. [Leguminosae; Glycyrrhizae Radix] 6 g, Ephedra sinica Stapf. [Ephedraceae; Ephedrae Herba] 3 g, Zizyphus jujuba var. inermis Rehder [Rhamnaceae; Zizyphi Fructus], Zingiber officinale Rosc. [Zingiberaceae; Zingiberis Rhizoma Recens] 3 pieces | None | 10d | None | N / N |
| Chen 2016 | Biyuan tongqiao granule | Granule | Magnolia denudata Desr. [Magnoliaceae; Magnoliae Flos], Xanthium strumarium L. [Asteraceae; Xanthii Fructus], Ephedra sinica Stapf. [Ephedraceae; Ephedrae Herba], Angelica dahurica Benth. et Hooker f. [Apiaceae; Angelicae Dahuricae Radix], Mentha arvensis var. piperascens Makinv. [Lamiaceae; Menthae Herba], Scutellaria baicalensis Georgi [Labiatae; Scutellariae Radix], Forsythia suspensa (Thunb.) Vahl [Oleaceae; Forsythiae Fructus], Chrysanthemum indicum L [Asteraceae; Chrysanthemi Indici Flos], Trichosanthes kirilowii Maxim. [Cucurbitaceae; Trichosanthis Radix], Rhemannia glutinosa (Gaertner) Liboschitz [Scrophulariaceae; Rehmanniae Radix], Salvia miltiorrhiza Bunge. [Labiatae; Salviae Miltiorrhizae Radix] | None | 3mo | None | N / N |
| Chen 2017 | Biyuanshu oral liquid | Oral liquid | Xanthium strumarium L. [Asteraceae; Xanthii Fructus], Magnolia denudata Desr. [Magnoliaceae; Magnoliae Flos], Angelica dahurica Benth. et Hooker f. [Apiaceae; Angelicae Dahuricae Radix], Bupleurum falcatum Linne [Apiaceae; Bupleuri Radix], Scutellaria baicalensis Georgi [Labiatae; Scutellariae Radix], Astragalus membranaceus Bunge [Leguminosae; Astragali Radix] | None | NR | None | Y – produced by Chengdu Huashen Group Co., Ltd. / N |
| Chen 2019a | Biyuan tongqiao granule | Granule | Angelica dahurica Benth. et Hooker f. [Apiaceae; Angelicae Dahuricae Radix], Magnolia denudata Desr. [Magnoliaceae; Magnoliae Flos], Xanthium strumarium L. [Asteraceae; Xanthii Fructus], Ephedra sinica Stapf. [Ephedraceae; Ephedrae Herba], Mentha arvensis var. piperascens Makinv. [Lamiaceae; Menthae Herba], Angelica tenuissima Nakai [Apiaceae; Ligustici Tenuissimae Radix], Scutellaria baicalensis Georgi [Labiatae; Scutellariae Radix], Forsythia suspensa (Thunb.) Vahl [Oleaceae; Forsythiae Fructus], Chrysanthemum indicum L [Asteraceae; Chrysanthemi Indici Flos], Trichosanthes kirilowii Maxim. [Cucurbitaceae; Trichosanthis Radix], Rhemannia glutinosa (Gaertner) Liboschitz [Scrophulariaceae; Rehmanniae Radix], Salvia miltiorrhiza Bunge. [Labiatae; Salviae Miltiorrhizae Radix], Poria cocos (Schw.) Wolf [Polyporaceae; Poria(Hoelen)], Glycyrrhiza uralensis Fisch. [Leguminosae; Glycyrrhizae Radix] | None | 4wks | None | Y – produced by Shandong New Times Pharmaceutical Co., Ltd. / N |
| Chen 2019b | Qīngxuān bí qiào yǐn | Decoction | Taraxacum platycarpum H. Dahlsi [Asteraceae; Taraxci Herba] 20 g, Magnolia denudata Desr. [Magnoliaceae; Magnoliae Flos], Forsythia suspensa (Thunb.) Vahl [Oleaceae; Forsythiae Fructus], Coix lachryma-jobi var. ma-yeun (Roman.) Stapf [Gramineae; Coicis Semen], Ligusticum chuanxiong Hort [Apiaceae; Ligustici Rhizoma], Trichosanthes kirilowii Maxim. [Cucurbitaceae; Trichosanthis Radix] 15 g, Xanthium strumarium L. [Asteraceae; Xanthii Fructus], Scutellaria baicalensis Georgi [Labiatae; Scutellariae Radix], Bombyx mori L. [Bombycide; Bombycis Corpus cum Batryticatus] 12 g, Arctium lappa L. [Asteraceae; Arctii Fructus] 10 g, Mentha arvensis var. piperascens Makinv. [Lamiaceae; Menthae Herba], Angelica dahurica Benth. et Hooker f. [Apiaceae; Angelicae Dahuricae Radix], Phragmites communis Trin. [Gramineae; Phragmitis Rhizoma], Scrophularia buergeriana Miq. [Scrophulariaceae; Scrophulariae Radix] 9 g, Prunus armeniaca L. var. ansu Maxim. [Rosaceae; Armeniacae Semen] 8 g, Platycodon grandiflorum (Jacq.) A. DC. [Campanulaceae; Platycodi Radix], Fritillaria thunbergii Miq. [Liliaceae; Fritillariae Thunbergii Bulbus], Glycyrrhiza uralensis Fisch. [Leguminosae; Glycyrrhizae Radix] 6 g, Belamcanda chinensis (L.) DC. [Iridaceae; Belamcandae Rhizoma] 4 g | - sore throat: Isatis indigotica Fort [Brassicaceae; Isatidis Radix]  - severe yellow rhinorrhea: Lonicera japonica Thunb. [Caprifoliaceae; Lonicerae Flos], Saururus chinensis Baill. [Saururaceae; Houttuyniae Herba]  - severe cough: Morus alba L. [Moraceae; Mori Radicis Cortex]  - nasal dryness: Liriope Platyphylla Wang et Tang [Liliaceae; Liriopes Radix], Glehnia littoralis Fr. Schm. [Apiaceae; Glehniae Radix], Dendrobium loddigesii Rolfe. [Orchidaceae; Dendrobii Herba]  - adenoid hypertrophy: Boswellia carterii Birdw. [Burseraceae; Olibanum], Commiphora myrrha Engl. [Burseraceae; Myrrha], Gleditsia japonica var. korainensis (Nak.) Nakai [Leguminosae; Gleditsiae Semen]  - severe headache: Angelica tenuissima Nakai [Apiaceae; Ligustici Tenuissimae Radix]  - constipation: Trichosanthes kirilowii Maxim. [Cucurbitaceae; Trichosanthis Fuctus] 12 g | 2wks | None | N / N |
| Dai 2013 | Bitong mixture | NR | Xanthium strumarium L. [Asteraceae; Xanthii Fructus], Angelica dahurica Benth. et Hooker f. [Apiaceae; Angelicae Dahuricae Radix], Magnolia denudata Desr. [Magnoliaceae; Magnoliae Flos], Poria cocos (Schw.) Wolf [Polyporaceae; Poria(Hoelen)], Coix lachryma-jobi var. ma-yeun (Roman.) Stapf [Gramineae; Coicis Semen], Scutellaria baicalensis Georgi [Labiatae; Scutellariae Radix], Saururus chinensis Baill. [Saururaceae; Houttuyniae Herba], Ligusticum chuanxiong Hort [Apiaceae; Ligustici Rhizoma], Astragalus membranaceus Bunge [Leguminosae; Astragali Radix] | None | 3mo | 12mo | N / N |
| Ding 2012 | Biyuan decoction | Decoction | Scutellaria baicalensis Georgi [Labiatae; Scutellariae Radix], Prunella vulgaris var. lilacina Nakai [Lamiaceae; Prunellae Spica], Plantago asiatica L. [Plantaginaceae; Plantaginis Herba], Alisma orientalis (Sam) Juzep [Alismataceae; Alismatis Rhizoma], Magnolia denudata Desr. [Magnoliaceae; Magnoliae Flos], Xanthium strumarium L. [Asteraceae; Xanthii Fructus], Fructus liquidambaris [Liquidambar formosana Hance.], Taraxacum platycarpum H. Dahlsi [Asteraceae; Taraxci Herba] 10 g, Gentiana scabra Bunge [Gentianaceae; Gentianae Radix] 3 g | - severe headache: Angelica dahurica Benth. et Hooker f. [Apiaceae; Angelicae Dahuricae Radix], Ligusticum chuanxiong Hort [Apiaceae; Ligustici Rhizoma] 10 g  - nasal pus, fishy smell: Lonicera japonica Thunb. [Caprifoliaceae; Lonicerae Flos], Forsythia suspensa (Thunb.) Vahl [Oleaceae; Forsythiae Fructus], Saururus chinensis Baill. [Saururaceae; Houttuyniae Herba] 10 g  - fatigue, spirit malaise: Astragalus membranaceus Bunge [Leguminosae; Astragali Radix] 10 g | 3wks | None | N / N |
| Du 2016 | Qingbi decoction | Decoction | Astragalus membranaceus Bunge [Leguminosae; Astragali Radix] 30 g, Phragmites communis Trin. [Gramineae; Phragmitis Rhizoma], Saururus chinensis Baill. [Saururaceae; Houttuyniae Herba] 15 g, Scutellaria baicalensis Georgi [Labiatae; Scutellariae Radix], Mentha arvensis var. piperascens Makinv. [Lamiaceae; Menthae Herba], Platycodon grandiflorum (Jacq.) A. DC. [Campanulaceae; Platycodi Radix], Lonicera japonica Thunb. [Caprifoliaceae; Lonicerae Flos], Angelica gigas Nakai [Apiaceae; Angelicae Gigantis Radix] 10 g, Magnolia denudata Desr. [Magnoliaceae; Magnoliae Flos], Angelica dahurica Benth. et Hooker f. [Apiaceae; Angelicae Dahuricae Radix], Xanthium strumarium L. [Asteraceae; Xanthii Fructus], Glycyrrhiza uralensis Fisch. [Leguminosae; Glycyrrhizae Radix] 6 g | None | 8wks | 3mo | N / N |
| Fan 2020 | No name | Decoction | -dampness-heat: Bupleurum falcatum Linne [Apiaceae; Bupleuri Radix], Scutellaria baicalensis Georgi [Labiatae; Scutellariae Radix], Xanthium strumarium L. [Asteraceae; Xanthii Fructus], Ligusticum chuanxiong Hort [Apiaceae; Ligustici Rhizoma], Angelica gigas Nakai [Apiaceae; Angelicae Gigantis Radix] 15 g, Gardenia jasminoides var. grandiflora (Lour.) Nakai [Rubiaceae; Gardeniae Fructus], Platycodon grandiflorum (Jacq.) A. DC. [Campanulaceae; Platycodi Radix], Angelica dahurica Benth. et Hooker f. [Apiaceae; Angelicae Dahuricae Radix], Saururus chinensis Baill. [Saururaceae; Houttuyniae Herba], Agastache rugosa (Fisch. et Meyer) O. Kuntze [Lamiaceae; Agastachis Herba], Saposhnikovia divaricata Schiskin [Apiaceae; Saposhnikovia Radix], Magnolia denudata Desr. [Magnoliaceae; Magnoliae Flos], Akebia quinata Decne. [Lardizabalaceae; Akebiae Caulis] 10 g, Glycyrrhiza uralensis Fisch. [Leguminosae; Glycyrrhizae Radix] 6 g  -deficiency cold pattern: Codonopsis pilosulae (Fr.) Nannf. [Campunulaceae; Codonopsis Pilosulae Radix] 20 g, Poria cocos (Schw.) Wolf [Polyporaceae; Poria(Hoelen)], Bupleurum falcatum Linne [Apiaceae; Bupleuri Radix], Xanthium strumarium L. [Asteraceae; Xanthii Fructus], Ligusticum chuanxiong Hort [Apiaceae; Ligustici Rhizoma] 15 g, Platycodon grandiflorum (Jacq.) A. DC. [Campanulaceae; Platycodi Radix], Angelica dahurica Benth. et Hooker f. [Apiaceae; Angelicae Dahuricae Radix], Magnolia denudata Desr. [Magnoliaceae; Magnoliae Flos], Saposhnikovia divaricata Schiskin [Apiaceae; Saposhnikovia Radix], Atractylodes macrocepha-la Koidz [Asteraceae; Atractylodis Rhizoma Alba], Zingiber officinale Rosc. [Zingiberaceae; Zingiberis Rhizoma Siccus] 10 g, Glycyrrhiza uralensis Fisch. [Leguminosae; Glycyrrhizae Radix] 6 g, Asarum sieboldii Miq. [Aristolochiaceae; Asari Herba Cum Radix] 5 g | None | 14d | None | N / N |
| Fu 2020 | Sì huáng bí yuān fāng | Decoction | Astragalus membranaceus Bunge [Leguminosae; Astragali Radix], Scutellaria baicalensis Georgi [Labiatae; Scutellariae Radix], Phellodendron amurense Rupr. [Rutaceae; Phellodendri Cortex] 15 g, Coptis deltoidea C.Y. Cheng et Hsiao [Ranunculaceae; Coptidis Rhizoma], Chrysanthemum morifolium Ramat. [Asteraceae; Chrysanthemi Flos], Glycyrrhiza uralensis Fisch. [Leguminosae; Glycyrrhizae Radix], Angelica dahurica Benth. et Hooker f. [Apiaceae; Angelicae Dahuricae Radix], Forsythia suspensa (Thunb.) Vahl [Oleaceae; Forsythiae Fructus], Ligusticum chuanxiong Hort [Apiaceae; Ligustici Rhizoma], Bupleurum falcatum Linne [Apiaceae; Bupleuri Radix] 10 g, Xanthium strumarium L. [Asteraceae; Xanthii Fructus], Dryobalanops aromatica Gaertn. f. [Dipterocarpaceae; Borneolum], Paeonia lactiflora Pall. [Paeoniaceae; Paeoniae Radix Rubra] 5 g | None | 4wks | None | N / N |
| Gou 2020 | Biyuan tongqiao granule | Granule | Magnolia denudata Desr. [Magnoliaceae; Magnoliae Flos], Ephedra sinica Stapf. [Ephedraceae; Ephedrae Herba], Angelica dahurica Benth. et Hooker f. [Apiaceae; Angelicae Dahuricae Radix], Forsythia suspensa (Thunb.) Vahl [Oleaceae; Forsythiae Fructus], Trichosanthes kirilowii Maxim. [Cucurbitaceae; Trichosanthis Radix], Salvia miltiorrhiza Bunge. [Labiatae; Salviae Miltiorrhizae Radix], Mentha arvensis var. piperascens Makinv. [Lamiaceae; Menthae Herba], Xanthium strumarium L. [Asteraceae; Xanthii Fructus], Scutellaria baicalensis Georgi [Labiatae; Scutellariae Radix], Rhemannia glutinosa (Gaertner) Liboschitz [Scrophulariaceae; Rehmanniae Radix] | None | 14d | None | Y – produced by Shandong New Times Pharmaceutical Co., Ltd. / N |
| He 2020 | Shufeng Tongqiao decoction | Decoction | Salvia miltiorrhiza Bunge. [Labiatae; Salviae Miltiorrhizae Radix] 30 g, Sophora flavescens Ait. [Leguminosae; Sophorae Radix], Poria cocos (Schw.) Wolf [Polyporaceae; Poria(Hoelen)] 20 g, Ligusticum chuanxiong Hort [Apiaceae; Ligustici Rhizoma], Atractylodes lancea (Thunb.) DC. [Asteraceae; Atractylodis Rhizoma], Potentilla discolor Bunge [Rosaceae; Potentillae Discoloris Herba], Glycyrrhiza uralensis Fisch. [Leguminosae; Glycyrrhizae Radix] 10 g, Mentha arvensis var. piperascens Makinv. [Lamiaceae; Menthae Herba], Angelica dahurica Benth. et Hooker f. [Apiaceae; Angelicae Dahuricae Radix], Magnolia denudata Desr. [Magnoliaceae; Magnoliae Flos], Xanthium strumarium L. [Asteraceae; Xanthii Fructus] 6 g, Cryptotympana pustulata Fabricius [Cicadidae; Cicadae Periostracum] 3 g | None | 4wks | 6mo | N / N |
| Hou 2018 | No name | Decoction | Xanthium strumarium L. [Asteraceae; Xanthii Fructus], Saururus chinensis Baill. [Saururaceae; Houttuyniae Herba] 12 g, Ligusticum chuanxiong Hort [Apiaceae; Ligustici Rhizoma], Angelica dahurica Benth. et Hooker f. [Apiaceae; Angelicae Dahuricae Radix] 9 g, Saposhnikovia divaricata Schiskin [Apiaceae; Saposhnikovia Radix], Magnolia denudata Desr. [Magnoliaceae; Magnoliae Flos], Bupleurum falcatum Linne [Apiaceae; Bupleuri Radix], Glycyrrhiza uralensis Fisch. [Leguminosae; Glycyrrhizae Radix] 6 g, Asarum sieboldii Miq. [Aristolochiaceae; Asari Herba Cum Radix] 2 g | None | 30d | 3mo | N / N |
| Hu 2019 | Bufei Yipi Tongqiao decoction | Decoction | Codonopsis pilosulae (Fr.) Nannf. [Campunulaceae; Codonopsis Pilosulae Radix], Astragalus membranaceus Bunge [Leguminosae; Astragali Radix] 18 g, Poria cocos (Schw.) Wolf [Polyporaceae; Poria(Hoelen)], Atractylodes macrocepha-la Koidz [Asteraceae; Atractylodis Rhizoma Alba], Dioscorea batatas Decne. [Dioscoreaceae; Dioscoreae Rhizoma], Coix lachryma-jobi var. ma-yeun (Roman.) Stapf [Gramineae; Coicis Semen], Nelumbo nucifera Gaertner [Nymphaceae; Nelumbinis Semen] 15 g, Saposhnikovia divaricata Schiskin [Apiaceae; Saposhnikovia Radix], Platycodon grandiflorum (Jacq.) A. DC. [Campanulaceae; Platycodi Radix], Schizonepeta tenuifolia (Benth.) Briq. [Labiatae; Schizonepetae Spica], Centipeda minima (L.) A. Br. Et Aschers. [Asteraceae; Centipedae Herba], Glycyrrhiza uralensis Fisch. [Leguminosae; Glycyrrhizae Radix] 10 g, Asarum sieboldii Miq. [Aristolochiaceae; Asari Herba Cum Radix] 5 g | None | 4wks | 3mo | N / N |
| Huang 2015 | Qingyaun decoction | Decoction | Scutellaria baicalensis Georgi [Labiatae; Scutellariae Radix], Salvia miltiorrhiza Bunge. [Labiatae; Salviae Miltiorrhizae Radix], Patrinia villosa (Thunb.) Juss [Valerianaceae; Patriniae Radix], Fructus trichosanthis [Trichosanthes Hisrilowii], Saururus chinensis Baill. [Saururaceae; Houttuyniae Herba] 20 g, Astragalus membranaceus Bunge [Leguminosae; Astragali Radix] 15 g, Acorus gramineus Sol. ex Aiton [Acoraceae; Acori Graminei Rhizoma], Angelica dahurica Benth. et Hooker f. [Apiaceae; Angelicae Dahuricae Radix], Paeonia lactiflora Pall. [Paeoniaceae; Paeoniae Radix Rubra], Chrysanthemum morifolium Ramat. [Asteraceae; Chrysanthemi Flos], Polygala tenuifolia Willd. [Polygalaceae; Polygalae Radix] 10 g, Xanthium strumarium L. [Asteraceae; Xanthii Fructus], Platycodon grandiflorum (Jacq.) A. DC. [Campanulaceae; Platycodi Radix], Magnolia denudata Desr. [Magnoliaceae; Magnoliae Flos], Gleditsia japonica var. korainensis (Nak.) Nakai [Leguminosae; Gleditsiae Semen] 6 g | None | 10d | None | N / N |
| Huang 2020 | Jianpi Hauzhuo Tongqiao decoction | Decoction | Astragalus membranaceus Bunge [Leguminosae; Astragali Radix], Poria cocos (Schw.) Wolf [Polyporaceae; Poria(Hoelen)], Coix lachryma-jobi var. ma-yeun (Roman.) Stapf [Gramineae; Coicis Semen] 30 g, Paeonia albiflora Pallas var. trichocarpa Bunge [Paeoniaceae; Paeoniae Radix Alba], Trichosanthes kirilowii Maxim. [Cucurbitaceae; Trichosanthis Radix], Atractylodes macrocepha-la Koidz [Asteraceae; Atractylodis Rhizoma Alba], Amomum villosum Lour. [Zingiberaceae; Amomi Fuctus] 15 g, Citrus unshiu Markovich [Rutaceae; Citri Pericarpium] 12 g, Eupatorium chinense for. tripartitum H. Hara [Asteraceae; Eupatorii Herba], Agastache rugosa (Fisch. et Meyer) O. Kuntze [Lamiaceae; Agastachis Herba], Platycodon grandiflorum (Jacq.) A. DC. [Campanulaceae; Platycodi Radix] 10 g, Angelica dahurica Benth. et Hooker f. [Apiaceae; Angelicae Dahuricae Radix], Cimicifuga heracleifolia Kom. [Ranunculaceae; Cimicifugae Rhizoma], Acorus gramineus Sol. ex Aiton [Acoraceae; Acori Graminei Rhizoma] 9 g, Glycyrrhiza uralensis Fisch. [Leguminosae; Glycyrrhizae Radix] 5 g | None | 4wks | None | N / N |
| Jiang 2012 | Tsang-Erh-San extract and Houttuynia extract powder | Capsule | Angelica dahurica Benth. et Hooker f. [Apiaceae; Angelicae Dahuricae Radix], Magnolia denudata Desr. [Magnoliaceae; Magnoliae Flos], Xanthium strumarium L. [Asteraceae; Xanthii Fructus], Mentha arvensis var. piperascens Makinv. [Lamiaceae; Menthae Herba], Saururus chinensis Baill. [Saururaceae; Houttuyniae Herba] | None | 8wks | None | N / N |
| Jiang 2021a | Xiāo fēng xuān fèi tāng | Decoction | Lonicera japonica Thunb. [Caprifoliaceae; Lonicerae Flos], Forsythia suspensa (Thunb.) Vahl [Oleaceae; Forsythiae Fructus], Chrysanthemum morifolium Ramat. [Asteraceae; Chrysanthemi Flos] 9 g, Scutellaria baicalensis Georgi [Labiatae; Scutellariae Radix], Bombyx mori L. [Bombycide; Bombycis Corpus cum Batryticatus], Oroxylum indicum (Linn.) Kurz [Semen Oroxyli], Fritillaria cirrhosa D. Don. [Liliaceae; Fritillariae Cirrhosae Bulbus], Prunus armeniaca L. var. ansu Maxim. [Rosaceae; Armeniacae Semen] 6 g, Ephedra sinica Stapf. [Ephedraceae; Ephedrae Herba], Glycyrrhiza uralensis Fisch. [Leguminosae; Glycyrrhizae Radix] 3 g | None | 1mo | None | N / N |
| Jiang 2021b | Biyuan tongqiao granule | Granule | Angelica tenuissima Nakai [Apiaceae; Ligustici Tenuissimae Radix], Ephedra sinica Stapf. [Ephedraceae; Ephedrae Herba], Xanthium strumarium L. [Asteraceae; Xanthii Fructus], Poria cocos (Schw.) Wolf [Polyporaceae; Poria(Hoelen)], Angelica dahurica Benth. et Hooker f. [Apiaceae; Angelicae Dahuricae Radix], Magnolia denudata Desr. [Magnoliaceae; Magnoliae Flos], Mentha arvensis var. piperascens Makinv. [Lamiaceae; Menthae Herba], Chrysanthemum indicum L [Asteraceae; Chrysanthemi Indici Flos], Rhemannia glutinosa (Gaertner) Liboschitz [Scrophulariaceae; Rehmanniae Radix], Scutellaria baicalensis Georgi [Labiatae; Scutellariae Radix] | None | 10wks | 6mo | Y – produced by Shandong New Times Pharmaceutical Co., Ltd. / N |
| Li 2015 | Orifice-opening, Phlegm-Eliminating and Sinus-clearing decoction | Decoction | Atractylodes lancea (Thunb.) DC. [Asteraceae; Atractylodis Rhizoma], Magnolia officinalis Rehder et Wilson [Magnoliaceae; Magnoliae Cortex], Saposhnikovia divaricata Schiskin [Apiaceae; Saposhnikovia Radix], Magnolia denudata Desr. [Magnoliaceae; Magnoliae Flos], Forsythia suspensa (Thunb.) Vahl [Oleaceae; Forsythiae Fructus], Triticum aestivum L. [Gramineae; Massa Medicata Fermentata], Perilla frutescens var. acuta Kudo [Labiatae; Perilliae Semen], Agastache rugosa (Fisch. et Meyer) O. Kuntze [Lamiaceae; Agastachis Herba], Eupatorium chinense for. tripartitum H. Hara [Asteraceae; Eupatorii Herba], Bombyx mori L. [Bombycide; Bombycis Corpus cum Batryticatus], Centipeda minima (L.) A. Br. Et Aschers. [Asteraceae; Centipedae Herba], Angelica dahurica Benth. et Hooker f. [Apiaceae; Angelicae Dahuricae Radix] 6 g, Prunus armeniaca L. var. ansu Maxim. [Rosaceae; Armeniacae Semen], Glycyrrhiza uralensis Fisch. [Leguminosae; Glycyrrhizae Radix] 4 g | None | 30d | None | N / N |
| Li 2018a | Xinzhi Tongqiao granule | Granule | Magnolia denudata Desr. [Magnoliaceae; Magnoliae Flos], Xanthium strumarium L. [Asteraceae; Xanthii Fructus], Angelica dahurica Benth. et Hooker f. [Apiaceae; Angelicae Dahuricae Radix], Mentha arvensis var. piperascens Makinv. [Lamiaceae; Menthae Herba], Ephedra sinica Stapf. [Ephedraceae; Ephedrae Herba], Lonicera japonica Thunb. [Caprifoliaceae; Lonicerae Flos], Scutellaria baicalensis Georgi [Labiatae; Scutellariae Radix], Gypsum [Gypsum Fibrosum], Saururus chinensis Baill. [Saururaceae; Houttuyniae Herba], Ligusticum chuanxiong Hort [Apiaceae; Ligustici Rhizoma], Paeonia suffruticosa Andrews [Ranunculaceae; Moutan Cortex], Zanthoxylum piperitum A.P. Dc. [Rutaceae; Zanthoxyli Pericarpium], Citrus unshiu Markovich [Rutaceae; Citri Pericarpium], Glycyrrhiza uralensis Fisch. [Leguminosae; Glycyrrhizae Radix] | None | 20d | None | N / N |
| Li 2018b | Biyuan decoction | Decoction | Astragalus membranaceus Bunge [Leguminosae; Astragali Radix] 30 g, Xanthium strumarium L. [Asteraceae; Xanthii Fructus], Magnolia denudata Desr. [Magnoliaceae; Magnoliae Flos] 15 g, Angelica gigas Nakai [Apiaceae; Angelicae Gigantis Radix], Atractylodes macrocepha-la Koidz [Asteraceae; Atractylodis Rhizoma Alba], Saururus chinensis Baill. [Saururaceae; Houttuyniae Herba] 12 g, Scutellaria baicalensis Georgi [Labiatae; Scutellariae Radix], Codonopsis pilosulae (Fr.) Nannf. [Campunulaceae; Codonopsis Pilosulae Radix], Acorus gramineus Sol. ex Aiton [Acoraceae; Acori Graminei Rhizoma] 10 g, Akebia quinata Decne. [Lardizabalaceae; Akebiae Caulis] 8 g, Glycyrrhiza uralensis Fisch. [Leguminosae; Glycyrrhizae Radix] 6 g | - severe nasal obstruction: Mentha arvensis var. piperascens Makinv. [Lamiaceae; Menthae Herba] 9 g - headache: Angelica tenuissima Nakai [Apiaceae; Ligustici Tenuissimae Radix] 9 g - rhinorrhea: Patrinia villosa (Thunb.) Juss [Valerianaceae; Patriniae Radix] 12 g - temporalis pain: Angelica dahurica Benth. et Hooker f. [Apiaceae; Angelicae Dahuricae Radix] 12 g, Ligusticum chuanxiong Hort [Apiaceae; Ligustici Rhizoma] 9 g | 28d | None | N / N |
| Li 2021 | Tongqiao Biyan pill | Pill | Scutellaria baicalensis Georgi [Labiatae; Scutellariae Radix], Gardenia jasminoides var. grandiflora (Lour.) Nakai [Rubiaceae; Gardeniae Fructus], Magnolia denudata Desr. [Magnoliaceae; Magnoliae Flos], Angelica dahurica Benth. et Hooker f. [Apiaceae; Angelicae Dahuricae Radix], Xanthium strumarium L. [Asteraceae; Xanthii Fructus], Mentha arvensis var. piperascens Makinv. [Lamiaceae; Menthae Herba], Glycyrrhiza uralensis Fisch. [Leguminosae; Glycyrrhizae Radix] | None | 1mo | None | Y – produced by Shandong Huayang Pharmaceutical Co., Ltd. / N |
| Liang 2004 | Biyuanshu oral liquid | Oral liquid | Magnolia denudata Desr. [Magnoliaceae; Magnoliae Flos], Xanthium strumarium L. [Asteraceae; Xanthii Fructus], Astragalus membranaceus Bunge [Leguminosae; Astragali Radix], Angelica dahurica Benth. et Hooker f. [Apiaceae; Angelicae Dahuricae Radix], Bupleurum falcatum Linne [Apiaceae; Bupleuri Radix], Scutellaria baicalensis Georgi [Labiatae; Scutellariae Radix] | None | 20d | 60d | N / N |
| Liao 2020 | Biyankang decoction | Decoction | Rehmannia glutinosa var. purpurea (Makino) Makino et Nemoto [Scrophulariaceae; Rehmanniae Radix], Lonicera japonica Thunb. [Caprifoliaceae; Lonicerae Flos], Chrysanthemum morifolium Ramat. [Asteraceae; Chrysanthemi Flos] 15 g, Angelica gigas Nakai [Apiaceae; Angelicae Gigantis Radix], Ligusticum chuanxiong Hort [Apiaceae; Ligustici Rhizoma], Acorus gramineus Sol. ex Aiton [Acoraceae; Acori Graminei Rhizoma], Angelica dahurica Benth. et Hooker f. [Apiaceae; Angelicae Dahuricae Radix], Taraxacum platycarpum H. Dahlsi [Asteraceae; Taraxci Herba], Gardenia jasminoides var. grandiflora (Lour.) Nakai [Rubiaceae; Gardeniae Fructus], Schizonepeta tenuifolia (Benth.) Briq. [Labiatae; Schizonepetae Spica], Saposhnikovia divaricata Schiskin [Apiaceae; Saposhnikovia Radix], Magnolia denudata Desr. [Magnoliaceae; Magnoliae Flos], Xanthium strumarium L. [Asteraceae; Xanthii Fructus] 10 g, Glycyrrhiza uralensis Fisch. [Leguminosae; Glycyrrhizae Radix] 6 g, Asarum sieboldii Miq. [Aristolochiaceae; Asari Herba Cum Radix] 3 g | None | 2mo | None | N / N |
| Lin 2010 | - retained heat invading the lung meridian: Xinyiqingfei yin - dampness-heat in the spleen and stomach, liver and gallbladder:   Huangqinhuashi decoction and Cangerzi - dual deficiency of the lung-spleen:  Shenlingbaizhu powder and Cangerzi powder | Decoction | - retained heat invading the lung meridian: Coix lachryma-jobi var. ma-yeun (Roman.) Stapf [Gramineae; Coicis Semen] 30 g, Trichosanthes kirilowii Maxim. [Cucurbitaceae; Trichosanthis Semen] 15 g, Liriope Platyphylla Wang et Tang [Liliaceae; Liriopes Radix] 12 g, Magnolia denudata Desr. [Magnoliaceae; Magnoliae Flos], Gardenia jasminoides var. grandiflora (Lour.) Nakai [Rubiaceae; Gardeniae Fructus], Scutellaria baicalensis Georgi [Labiatae; Scutellariae Radix], Angelica dahurica Benth. et Hooker f. [Apiaceae; Angelicae Dahuricae Radix], Xanthium strumarium L. [Asteraceae; Xanthii Fructus] 10 g, Anemarrhena asphodeloides Bunge [Haemodoraceae; Anemarrhenae Rhizoma] 8 g, Glycyrrhiza uralensis Fisch. [Leguminosae; Glycyrrhizae Radix] 6 g  - dampness-heat in the spleen and stomach, liver and gallbladder: Talc [Talcum], Coix lachryma-jobi var. ma-yeun (Roman.) Stapf [Gramineae; Coicis Semen] 30 g, Scutellaria baicalensis Georgi [Labiatae; Scutellariae Radix], Alisma orientalis (Sam) Juzep [Alismataceae; Alismatis Rhizoma] 15 g, Gardenia jasminoides var. grandiflora (Lour.) Nakai [Rubiaceae; Gardeniae Fructus], Morus alba L. [Moraceae; Mori Radicis Cortex], Paeonia lactiflora Pall. [Paeoniaceae; Paeoniae Radix Rubra], Angelica dahurica Benth. et Hooker f. [Apiaceae; Angelicae Dahuricae Radix], Xanthium strumarium L. [Asteraceae; Xanthii Fructus], Magnolia denudata Desr. [Magnoliaceae; Magnoliae Flos] 10 g, Mentha arvensis var. piperascens Makinv. [Lamiaceae; Menthae Herba] 8 g  - dual deficiency of the lung-spleen: Coix lachryma-jobi var. ma-yeun (Roman.) Stapf [Gramineae; Coicis Semen] 30 g, Dioscorea batatas Decne. [Dioscoreaceae; Dioscoreae Rhizoma] 25 g, Codonopsis pilosulae (Fr.) Nannf. [Campunulaceae; Codonopsis Pilosulae Radix], Poria cocos (Schw.) Wolf [Polyporaceae; Poria(Hoelen)], Atractylodes macrocepha-la Koidz [Asteraceae; Atractylodis Rhizoma Alba] 15 g, Xanthium strumarium L. [Asteraceae; Xanthii Fructus], Magnolia denudata Desr. [Magnoliaceae; Magnoliae Flos] 10 g, Angelica dahurica Benth. et Hooker f. [Apiaceae; Angelicae Dahuricae Radix] 8 g, Glycyrrhiza uralensis Fisch. [Leguminosae; Glycyrrhizae Radix], Amomum villosum Lour. [Zingiberaceae; Amomi Fuctus] 6 g | - many blood scabs in the surgical cavity, mucosal stasis, and qi stagnation and blood stasis: Bletilla striata Reichb. Fil. [Orchidaceae; Bletillae Rhizoma], Panax noto-ginseng (Burk) f. H. Chen [Araliaceae; Notoginseng Radix], Paeonia suffruticosa Andrews [Ranunculaceae; Moutan Cortex], Paeonia lactiflora Pall. [Paeoniaceae; Paeoniae Radix Rubra], Salvia miltiorrhiza Bunge. [Labiatae; Salviae Miltiorrhizae Radix] - operative cavity edema, vesicles, dampness turbidity: Agastache rugosa (Fisch. et Meyer) O. Kuntze [Lamiaceae; Agastachis Herba], Eupatorium chinense for. tripartitum H. Hara [Asteraceae; Eupatorii Herba], Plantago asiatica L. [Plantaginaceae; Plantaginis Semen], Alisma orientalis (Sam) Juzep [Alismataceae; Alismatis Rhizoma], Coix lachryma-jobi var. ma-yeun (Roman.) Stapf [Gramineae; Coicis Semen], Fructus liquidambaris [Liquidambar formosana Hance.] | 12wks | None | N / N |
| Lin 2013 | Biyuan tongqiao granule | Granule | Magnolia denudata Desr. [Magnoliaceae; Magnoliae Flos], Xanthium strumarium L. [Asteraceae; Xanthii Fructus], Ephedra sinica Stapf. [Ephedraceae; Ephedrae Herba], Angelica dahurica Benth. et Hooker f. [Apiaceae; Angelicae Dahuricae Radix], Mentha arvensis var. piperascens Makinv. [Lamiaceae; Menthae Herba], Angelica tenuissima Nakai [Apiaceae; Ligustici Tenuissimae Radix], Scutellaria baicalensis Georgi [Labiatae; Scutellariae Radix], Forsythia suspensa (Thunb.) Vahl [Oleaceae; Forsythiae Fructus], Chrysanthemum indicum L [Asteraceae; Chrysanthemi Indici Flos], Trichosanthes kirilowii Maxim. [Cucurbitaceae; Trichosanthis Radix], Rhemannia glutinosa (Gaertner) Liboschitz [Scrophulariaceae; Rehmanniae Radix], Salvia miltiorrhiza Bunge. [Labiatae; Salviae Miltiorrhizae Radix], Poria cocos (Schw.) Wolf [Polyporaceae; Poria(Hoelen)], Glycyrrhiza uralensis Fisch. [Leguminosae; Glycyrrhizae Radix] | None | 8wks | None | Y - Chinese medicine certificate Z20030071 / N |
| Lin 2017 | Biyuan tongqiao granule | Granule | Salvia miltiorrhiza Bunge. [Labiatae; Salviae Miltiorrhizae Radix], Xanthium strumarium L. [Asteraceae; Xanthii Fructus], Rhemannia glutinosa (Gaertner) Liboschitz [Scrophulariaceae; Rehmanniae Radix], Angelica dahurica Benth. et Hooker f. [Apiaceae; Angelicae Dahuricae Radix], Trichosanthes kirilowii Maxim. [Cucurbitaceae; Trichosanthis Radix], Chrysanthemum indicum L [Asteraceae; Chrysanthemi Indici Flos], Ephedra sinica Stapf. [Ephedraceae; Ephedrae Herba], Forsythia suspensa (Thunb.) Vahl [Oleaceae; Forsythiae Fructus], Mentha arvensis var. piperascens Makinv. [Lamiaceae; Menthae Herba], Scutellaria baicalensis Georgi [Labiatae; Scutellariae Radix], Magnolia denudata Desr. [Magnoliaceae; Magnoliae Flos] | None | 4wks | None | Y – produced by Shandong New Times Pharmaceutical Co., Ltd. / N |
| Lin 2020 | Lianhuaqingwen granule | Granule | Forsythia suspensa (Thunb.) Vahl [Oleaceae; Forsythiae Fructus], Lonicera japonica Thunb. [Caprifoliaceae; Lonicerae Flos], Ephedra sinica Stapf. [Ephedraceae; Ephedrae Herba], Prunus armeniaca L. var. ansu Maxim. [Rosaceae; Armeniacae Semen], Gypsum [Gypsum Fibrosum], Isatis indigotica Fort [Brassicaceae; Isatidis Radix], Dryopteris crassirhizoma Nakai [Aspidiaceae; Crassirhizomae Rhizoma], Saururus chinensis Baill. [Saururaceae; Houttuyniae Herba], Agastache rugosa (Fisch. et Meyer) O. Kuntze [Lamiaceae; Agastachis Herba], Rheum palmatum L. [Polygonaceae; Rhei Rhizoma], Rhodiola rosea L. [Radix Rhodiolae Crenulate; Crassulaceae], Mentha arvensis var. piperascens Makinv. [Lamiaceae; Menthae Oleum], Glycyrrhiza uralensis Fisch. [Leguminosae; Glycyrrhizae Radix] | None | 4wks | 30d | Y – produced by Beijing Yiling Pharmaceutical Co., Ltd. / N |
| Liu 2012 | Biyuanshu capsule | Capsule | Xanthium strumarium L. [Asteraceae; Xanthii Fructus], Magnolia denudata Desr. [Magnoliaceae; Magnoliae Flos], Mentha arvensis var. piperascens Makinv. [Lamiaceae; Menthae Herba], Angelica dahurica Benth. et Hooker f. [Apiaceae; Angelicae Dahuricae Radix], Scutellaria baicalensis Georgi [Labiatae; Scutellariae Radix], Gardenia jasminoides var. grandiflora (Lour.) Nakai [Rubiaceae; Gardeniae Fructus], Bupleurum falcatum Linne [Apiaceae; Bupleuri Radix], Asarum sieboldii Miq. [Aristolochiaceae; Asari Herba Cum Radix], Ligusticum chuanxiong Hort [Apiaceae; Ligustici Rhizoma], Astragalus membranaceus Bunge [Leguminosae; Astragali Radix], Akebia quinata Decne. [Lardizabalaceae; Akebiae Caulis], Platycodon grandiflorum (Jacq.) A. DC. [Campanulaceae; Platycodi Radix] | None | 12wks | None | Y – produced by Chengdu Huashen Group Co., Ltd. / N |
| Liu 2017 | Yupingfeng granule | Granule | Astragalus membranaceus Bunge [Leguminosae; Astragali Radix], Atractylodes macrocepha-la Koidz [Asteraceae; Atractylodis Rhizoma Alba], Saposhnikovia divaricata Schiskin [Apiaceae; Saposhnikovia Radix] | None | 4wks | 3mo | Y – produced by Guangdong Universal Pharmaceutical Co., Ltd. / N |
| Liu 2018 | Biyankang decoction | Decoction | Xanthium strumarium L. [Asteraceae; Xanthii Fructus], Magnolia denudata Desr. [Magnoliaceae; Magnoliae Flos], Saposhnikovia divaricata Schiskin [Apiaceae; Saposhnikovia Radix], Schizonepeta tenuifolia (Benth.) Briq. [Labiatae; Schizonepetae Spica], Chrysanthemum indicum L [Asteraceae; Chrysanthemi Indici Flos], Lonicera japonica Thunb. [Caprifoliaceae; Lonicerae Flos], Angelica gigas Nakai [Apiaceae; Angelicae Gigantis Radix] 10 g, Ephedra sinica Stapf. [Ephedraceae; Ephedrae Herba], Agastache rugosa (Fisch. et Meyer) O. Kuntze [Lamiaceae; Agastachis Herba], Glycyrrhiza uralensis Fisch. [Leguminosae; Glycyrrhizae Radix] 6 g, Asarum sieboldii Miq. [Aristolochiaceae; Asari Herba Cum Radix] 3 g | None | 4wks | None | N / N |
| Liu 2019 | Buqi zhuyang decoction | Decoction | Atractylodes macrocepha-la Koidz [Asteraceae; Atractylodis Rhizoma Alba], Astragalus membranaceus Bunge [Leguminosae; Astragali Radix] 30 g, Saposhnikovia divaricata Schiskin [Apiaceae; Saposhnikovia Radix], Agastache rugosa (Fisch. et Meyer) O. Kuntze [Lamiaceae; Agastachis Herba], Perilla fructescens var. acuta Kudo [Lamiaceae; Perilla Folium] 15 g, Angelica dahurica Benth. et Hooker f. [Apiaceae; Angelicae Dahuricae Radix], Schizonepeta tenuifolia (Benth.) Briq. [Labiatae; Schizonepetae Spica], Cinnamomum cassia Blume [Lauraceae; Cinnamomi Ramulus], Glycyrrhiza uralensis Fisch. [Leguminosae; Glycyrrhizae Radix] 10 g, Ephedra sinica Stapf. [Ephedraceae; Ephedrae Herba], Aconitum carmichaeli Debx [Ranunculaceae; Pulvis Aconiti Tuberis Purificatum], Terminalia chebula var. tomentella Kurt. [Combretaceae; Terminaliae Fructus] 5 g, Asarum sieboldii Miq. [Aristolochiaceae; Asari Herba Cum Radix] 3 g | - severe rhinorrhea:Poria cocos (Schw.) Wolf [Polyporaceae; Poria(Hoelen)], Citrus unshiu Markovich [Rutaceae; Citri Pericarpium] 15 g - severe headache and nasal obstruction: Ligusticum chuanxiong Hort [Apiaceae; Ligustici Rhizoma] 15 g, Magnolia denudata Desr. [Magnoliaceae; Magnoliae Flos], Xanthium strumarium L. [Asteraceae; Xanthii Fructus] 10 g - itchy nose and sneezing: Siegesbeckia glabrescens Makino [Asteraceae; Siegesbeckiae Herba], Lithospermum erythrorhizon S. et Z. [Borraginaceae; Lithospermi Radix] 15 g - severe symptoms of deficiency and cold: Alpinia officinarum Hance [Zingiberaceae; Alpiniae Officinarum Rhizoma] 10 g, Zanthoxylum piperitum A.P. Dc. [Rutaceae; Zanthoxyli Pericarpium] 5 g | 28d | None | N / N |
| Liu 2020 | Shensu wenfei decoction | Decoction | Perilla fructescens var. acuta Kudo [Lamiaceae; Perilla Folium], Cinnamomum cassia Blume [Lauraceae; Cinnamomi Cortex Spissus], Atractylodes macrocepha-la Koidz [Asteraceae; Atractylodis Rhizoma Alba], Panax ginseng C. A. Mey. [Araliaceae; Ginseng Radix] 25 g, Magnolia denudata Desr. [Magnoliaceae; Magnoliae Flos], Xanthium strumarium L. [Asteraceae; Xanthii Fructus], Pinellia ternata (Thunb.) Breit. [Araceae; Pinelliae Rhizoma] 20 g, Schizandra chinensis (Turcz.) Baill. [Magnoliaceae; Schizandrae Fructus], Poria cocos (Schw.) Wolf [Polyporaceae; Poria(Hoelen)], Citrus unshiu Markovich [Rutaceae; Citri Pericarpium] 10 g, Glycyrrhiza uralensis Fisch. [Leguminosae; Glycyrrhizae Radix] 8 g | None | 4wks | None | N / N |
| Lu 2011 | Tongbi decoction | Decoction | Poria cocos (Schw.) Wolf [Polyporaceae; Poria(Hoelen)] 12 g, Codonopsis pilosulae (Fr.) Nannf. [Campunulaceae; Codonopsis Pilosulae Radix], Atractylodes macrocepha-la Koidz [Asteraceae; Atractylodis Rhizoma Alba], Citrus unshiu Markovich [Rutaceae; Citri Pericarpium], Dioscorea batatas Decne. [Dioscoreaceae; Dioscoreae Rhizoma], Xanthium strumarium L. [Asteraceae; Xanthii Fructus], Magnolia denudata Desr. [Magnoliaceae; Magnoliae Flos], Angelica dahurica Benth. et Hooker f. [Apiaceae; Angelicae Dahuricae Radix] 10 g, Centipeda minima (L.) A. Br. Et Aschers. [Asteraceae; Centipedae Herba] 9 g, Mentha arvensis var. piperascens Makinv. [Lamiaceae; Menthae Herba] 6 g, Ephedra sinica Stapf. [Ephedraceae; Ephedrae Herba]3 g | None | 10d | None | N / N |
| Ma 2016 | Pudilan oral liquid | Oral liquid | Isatis indigotica Fort [Brassicaceae; Isatidis Radix], Taraxacum platycarpum H. Dahlsi [Asteraceae; Taraxci Herba], Scutellaria baicalensis Georgi [Labiatae; Scutellariae Radix], Corydalis bungeanaTurcz. [Papaveraceae; Corydalis (Tuber) Rhizoma] | None | 3mo | None | N / N |
| Ma 2020 | Shufeng Jiedu capsule | Capsule | Reynoutria japonica Houtt. [Polygonaceae; Polygoni Cuspidati Rhizoma], Forsythia suspensa (Thunb.) Vahl [Oleaceae; Forsythiae Fructus], Isatis indigotica Fort [Brassicaceae; Isatidis Radix], Bupleurum falcatum Linne [Apiaceae; Bupleuri Radix], Patrinia villosa (Thunb.) Juss [Valerianaceae; Patriniae Radix], Verbena officinalis L. [Verbenaceae; Verbenae Herba], Phragmites communis Trin. [Gramineae; Phragmitis Rhizoma], Glycyrrhiza uralensis Fisch. [Leguminosae; Glycyrrhizae Radix] | None | 90d | 3yr | Y – produced by Anhui Jiren Pharmaceutical Co., Ltd. / N |
| Peng 2020 | Wenyang huashi prescription | Decoction | Cinnamomum cassia Blume [Lauraceae; Cinnamomi Ramulus], Plantago asiatica L. [Plantaginaceae; Plantaginis Herba], Alisma orientalis (Sam) Juzep [Alismataceae; Alismatis Rhizoma] 15 g, Poria cocos (Schw.) Wolf [Polyporaceae; Poria(Hoelen)], Angelica dahurica Benth. et Hooker f. [Apiaceae; Angelicae Dahuricae Radix] 12 g, Magnolia denudata Desr. [Magnoliaceae; Magnoliae Flos], Xanthium strumarium L. [Asteraceae; Xanthii Fructus], Zingiber officinale Rosc. [Zingiberaceae; Zingiberis Rhizoma Siccus] 9 g, Aconitum carmichaeli Debx [Ranunculaceae; Pulvis Aconiti Tuberis Purificatum] 6 g | - deficiency symptom: Salvia miltiorrhiza Bunge. [Labiatae; Salviae Miltiorrhizae Radix], Atractylodes macrocepha-la Koidz [Asteraceae; Atractylodis Rhizoma Alba], Cimicifuga heracleifolia Kom. [Ranunculaceae; Cimicifugae Rhizoma] 9 g - excess symptom: Gentiana scabra Bunge [Gentianaceae; Gentianae Radix], Scutellaria baicalensis Georgi [Labiatae; Scutellariae Radix] 9 g | 3mo | None | N / N |
| Qian 2019 | Longdan Xiegan decoction | Decoction | Gentiana scabra Bunge [Gentianaceae; Gentianae Radix], Scutellaria baicalensis Georgi [Labiatae; Scutellariae Radix], Rehmannia glutinosa var. purpurea (Makino) Makino et Nemoto [Scrophulariaceae; Rehmanniae Radix], Gardenia jasminoides var. grandiflora (Lour.) Nakai [Rubiaceae; Gardeniae Fructus], Xanthium strumarium L. [Asteraceae; Xanthii Fructus], Angelica dahurica Benth. et Hooker f. [Apiaceae; Angelicae Dahuricae Radix], Saururus chinensis Baill. [Saururaceae; Houttuyniae Herba] 10 g, Pheretima aspergillum (E. Perrier) [Lumbricidae; Pheretimae Corpus] 7 g, Glycyrrhiza uralensis Fisch. [Leguminosae; Glycyrrhizae Radix], Mentha arvensis var. piperascens Makinv. [Lamiaceae; Menthae Herba] 6 g, Asarum sieboldii Miq. [Aristolochiaceae; Asari Herba Cum Radix] 3 g | None | 1mo | None | N / N |
| Shao 2019 | Xanthium powder | Decoction | Ligusticum chuanxiong Hort [Apiaceae; Ligustici Rhizoma] 12 g, Xanthium strumarium L. [Asteraceae; Xanthii Fructus] 10 g, Angelica dahurica Benth. et Hooker f. [Apiaceae; Angelicae Dahuricae Radix], Magnolia denudata Desr. [Magnoliaceae; Magnoliae Flos] 9 g, Asarum sieboldii Miq. [Aristolochiaceae; Asari Herba Cum Radix] 3 g, Mentha arvensis var. piperascens Makinv. [Lamiaceae; Menthae Herba] 6 g | - severe rhinorrhea: Atractylodes lancea (Thunb.) DC. [Asteraceae; Atractylodis Rhizoma], Atractylodes macrocepha-la Koidz [Asteraceae; Atractylodis Rhizoma Alba] 12 g - cough, lack of qi, loose stools, and other lung-spleen qi deficiency symptom: Poria cocos (Schw.) Wolf [Polyporaceae; Poria(Hoelen)], Astragalus membranaceus Bunge [Leguminosae; Astragali Radix] 15 g, Platycodon grandiflorum (Jacq.) A. DC. [Campanulaceae; Platycodi Radix] 9 g - halitosis, anorexia, and other spleen and stomach damp heat symptom: Taraxacum platycarpum H. Dahlsi [Asteraceae; Taraxci Herba] 30 g, Agastache rugosa (Fisch. et Meyer) O. Kuntze [Lamiaceae; Agastachis Herba] 10 g, Arisaema amurense var. serratum Nakai [Araceae; Arisaema Praeparatus cum Bile] 9 g - heat stagnation symptom in gallbladder meridian: Scutellaria baicalensis Georgi [Labiatae; Scutellariae Radix], Gardenia jasminoides var. grandiflora (Lour.) Nakai [Rubiaceae; Gardeniae Fructus] 10 g, Bupleurum falcatum Linne [Apiaceae; Bupleuri Radix] 9 g - wind-heat symtom in lung meridian: Lonicera japonica Thunb. [Caprifoliaceae; Lonicerae Flos] 12 g, Chrysanthemum morifolium Ramat. [Asteraceae; Chrysanthemi Flos] 10 g | 20d | None | N / N |
| Shen 2013 | Biyuan tongqiao granule | Granule | Magnolia denudata Desr. [Magnoliaceae; Magnoliae Flos], Xanthium strumarium L. [Asteraceae; Xanthii Fructus], Ephedra sinica Stapf. [Ephedraceae; Ephedrae Herba], Angelica dahurica Benth. et Hooker f. [Apiaceae; Angelicae Dahuricae Radix] | None | 4wks | None | N / N |
| Shen 2020 | Yupingfeng powder and Cangerzi powder | Decoction | Astragalus membranaceus Bunge [Leguminosae; Astragali Radix] 30 g, Codonopsis pilosulae (Fr.) Nannf. [Campunulaceae; Codonopsis Pilosulae Radix] 20 g, Angelica gigas Nakai [Apiaceae; Angelicae Gigantis Radix], Ligusticum chuanxiong Hort [Apiaceae; Ligustici Rhizoma], Salvia miltiorrhiza Bunge. [Labiatae; Salviae Miltiorrhizae Radix], Paeonia albiflora Pallas var. trichocarpa Bunge [Paeoniaceae; Paeoniae Radix Alba], Scutellaria baicalensis Georgi [Labiatae; Scutellariae Radix], Poria cocos (Schw.) Wolf [Polyporaceae; Poria(Hoelen)], Xanthium strumarium L. [Asteraceae; Xanthii Fructus] 15 g, Rosa laevigata Michx. [Rosaceae; Rosae Laevigatae Fructus] 12 g, Atractylodes macrocepha-la Koidz [Asteraceae; Atractylodis Rhizoma Alba], Angelica dahurica Benth. et Hooker f. [Apiaceae; Angelicae Dahuricae Radix] 10 g, Magnolia denudata Desr. [Magnoliaceae; Magnoliae Flos], Bupleurum falcatum Linne [Apiaceae; Bupleuri Radix], Fritillaria cirrhosa D. Don. [Liliaceae; Fritillariae Cirrhosae Bulbus], Mentha arvensis var. piperascens Makinv. [Lamiaceae; Menthae Herba], Saposhnikovia divaricata Schiskin [Apiaceae; Saposhnikovia Radix], Terminalia chebula var. tomentella Kurt. [Combretaceae; Terminaliae Fructus] 9 g, Citrus unshiu Markovich [Rutaceae; Citri Pericarpium] 8 g, Glycyrrhiza uralensis Fisch. [Leguminosae; Glycyrrhizae Radix] 6 g | - dry nose: Glehnia littoralis Fr. Schm. [Apiaceae; Glehniae Radix] 12 g, Liriope Platyphylla Wang et Tang [Liliaceae; Liriopes Radix] 9 g - severe yellow runny nose: Forsythia suspensa (Thunb.) Vahl [Oleaceae; Forsythiae Fructus] 12 g - severe itchy nose: Bombyx mori L. [Bombycide; Bombycis Corpus cum Batryticatus] 15 g - adenoid hypertrophy: Gleditsia japonica var. korainensis (Nak.) Nakai [Leguminosae; Gleditsiae Semen] 6 g - constipation: Trichosanthes kirilowii Maxim. [Cucurbitaceae; Trichosanthis Fuctus] 12 g - severe headache: Angelica tenuissima Nakai [Apiaceae; Ligustici Tenuissimae Radix] 10 g - excessive discharge in the nose: Platycodon grandiflorum (Jacq.) A. DC. [Campanulaceae; Platycodi Radix] 9 g | 12wks | 6mo | N / N |
| Song 2021 | Wenfei zhiliu pellet | Decoction | Pseudosciaena crocea (Richardson) [Sciaenidae; Pseudosciaenae Otolithum] 15 g, Terminalia chebula var. tomentella Kurt. [Combretaceae; Terminaliae Fructus] 12 g, Panax ginseng C. A. Mey. [Araliaceae; Ginseng Radix], Schizonepeta tenuifolia (Benth.) Briq. [Labiatae; Schizonepetae Spica], Platycodon grandiflorum (Jacq.) A. DC. [Campanulaceae; Platycodi Radix] 10 g, Asarum sieboldii Miq. [Aristolochiaceae; Asari Herba Cum Radix], Glycyrrhiza uralensis Fisch. [Leguminosae; Glycyrrhizae Radix] 6 g | - severe nasal itching: Bombyx mori L. [Bombycide; Bombycis Corpus cum Batryticatus], Cryptotympana pustulata Fabricius [Cicadidae; Cicadae Periostracum] 10 g - qi deficiency, more nasal discharge: Pinellia ternata (Thunb.) Breit. [Araceae; Pinelliae Rhizoma], Citrus unshiu Markovich [Rutaceae; Citri Pericarpium] 10 g, Astragalus membranaceus Bunge [Leguminosae; Astragali Radix] 30 g, Atractylodes macrocepha-la Koidz [Asteraceae; Atractylodis Rhizoma Alba] 15 g - fear of cold: Saposhnikovia divaricata Schiskin [Apiaceae; Saposhnikovia Radix], Cinnamomum cassia Blume [Lauraceae; Cinnamomi Ramulus], Zingiber officinale Rosc. [Zingiberaceae; Zingiberis Rhizoma Siccus] 10 g - stuffy nose: Xanthium strumarium L. [Asteraceae; Xanthii Fructus], Angelica dahurica Benth. et Hooker f. [Apiaceae; Angelicae Dahuricae Radix] 10 g | 12wks | None | N / N |
| Sun 2017 | Biyuanshu oral liquid | Oral liquid | Xanthium strumarium L. [Asteraceae; Xanthii Fructus], Magnolia denudata Desr. [Magnoliaceae; Magnoliae Flos], Astragalus membranaceus Bunge [Leguminosae; Astragali Radix], Bupleurum falcatum Linne [Apiaceae; Bupleuri Radix], Ligusticum chuanxiong Hort [Apiaceae; Ligustici Rhizoma], Platycodon grandiflorum (Jacq.) A. DC. [Campanulaceae; Platycodi Radix] | None | 3wks | None | Y – produced by Chengdu Huashen Group Co., Ltd. / N |
| Tang 2020 | Longdan Xiegan decoction | Decoction | Rehmannia glutinosa var. purpurea (Makino) Makino et Nemoto [Scrophulariaceae; Rehmanniae Radix] 20 g, Alisma orientalis (Sam) Juzep [Alismataceae; Alismatis Rhizoma] 12 g, Bupleurum falcatum Linne [Apiaceae; Bupleuri Radix] 10 g, Scutellaria baicalensis Georgi [Labiatae; Scutellariae Radix], Gardenia jasminoides var. grandiflora (Lour.) Nakai [Rubiaceae; Gardeniae Fructus], Akebia quinata Decne. [Lardizabalaceae; Akebiae Caulis], Plantago asiatica L. [Plantaginaceae; Plantaginis Semen] 9 g, Angelica gigas Nakai [Apiaceae; Angelicae Gigantis Radix] 8 g, Gentiana scabra Bunge [Gentianaceae; Gentianae Radix], Glycyrrhiza uralensis Fisch. [Leguminosae; Glycyrrhizae Radix] 6 g | None | 12wks | None | N / N |
| Tao 2020 | Tongqiao Biyan granule | Granule | Xanthium strumarium L. [Asteraceae; Xanthii Fructus], Ephedra sinica Stapf. [Ephedraceae; Ephedrae Herba], Astragalus membranaceus Bunge [Leguminosae; Astragali Radix], Angelica dahurica Benth. et Hooker f. [Apiaceae; Angelicae Dahuricae Radix], Magnolia denudata Desr. [Magnoliaceae; Magnoliae Flos], Mentha arvensis var. piperascens Makinv. [Lamiaceae; Menthae Herba] | None | 21d | None | Y – produced by Chengdu Dikang Pharmaceutical / N |
| Wang 2016 | Biyuan tongqiao granule | Granule | Magnolia denudata Desr. [Magnoliaceae; Magnoliae Flos], Angelica dahurica Benth. et Hooker f. [Apiaceae; Angelicae Dahuricae Radix], Xanthium strumarium L. [Asteraceae; Xanthii Fructus], Astragalus membranaceus Bunge [Leguminosae; Astragali Radix], Ephedra sinica Stapf. [Ephedraceae; Ephedrae Herba], Rhemannia glutinosa (Gaertner) Liboschitz [Scrophulariaceae; Rehmanniae Radix], Glycyrrhiza uralensis Fisch. [Leguminosae; Glycyrrhizae Radix], Salvia miltiorrhiza Bunge. [Labiatae; Salviae Miltiorrhizae Radix], Poria cocos (Schw.) Wolf [Polyporaceae; Poria(Hoelen)] | None | 10wks | None | Y – produced by Shandong New Times Pharmaceutical Co., Ltd. / N |
| Wang 2017 | Biyuan tongqiao granule | Granule | Scutellaria baicalensis Georgi [Labiatae; Scutellariae Radix], Ephedra sinica Stapf. [Ephedraceae; Ephedrae Herba], Mentha arvensis var. piperascens Makinv. [Lamiaceae; Menthae Herba], Forsythia suspensa (Thunb.) Vahl [Oleaceae; Forsythiae Fructus] | None | 4wks | None | N / N |
| Wang 2020a | Biyuan tongqiao granule | Granule | Xanthium strumarium L. [Asteraceae; Xanthii Fructus], Ephedra sinica Stapf. [Ephedraceae; Ephedrae Herba], Mentha arvensis var. piperascens Makinv. [Lamiaceae; Menthae Herba], Rhemannia glutinosa (Gaertner) Liboschitz [Scrophulariaceae; Rehmanniae Radix], Salvia miltiorrhiza Bunge. [Labiatae; Salviae Miltiorrhizae Radix], Chrysanthemum indicum L [Asteraceae; Chrysanthemi Indici Flos] | None | 6d | None | N / N |
| Wang 2020b | Tongqiao Biyan granule | Granule | Xanthium strumarium L. [Asteraceae; Xanthii Fructus], Saposhnikovia divaricata Schiskin [Apiaceae; Saposhnikovia Radix], Astragalus membranaceus Bunge [Leguminosae; Astragali Radix], Angelica dahurica Benth. et Hooker f. [Apiaceae; Angelicae Dahuricae Radix], Magnolia denudata Desr. [Magnoliaceae; Magnoliae Flos], Atractylodes macrocepha-la Koidz [Asteraceae; Atractylodis Rhizoma Alba], Mentha arvensis var. piperascens Makinv. [Lamiaceae; Menthae Herba] | None | 45d | None | Y – produced by Sichuan Chuanda Huaxi Pharmaceutical Co., Ltd. / N |
| Wei 2017 | No name | Decoction | Lonicera japonica Thunb. [Caprifoliaceae; Lonicerae Flos], Saururus chinensis Baill. [Saururaceae; Houttuyniae Herba] 20 g, Platycodon grandiflorum (Jacq.) A. DC. [Campanulaceae; Platycodi Radix], Agastache rugosa (Fisch. et Meyer) O. Kuntze [Lamiaceae; Agastachis Herba], Magnolia denudata Desr. [Magnoliaceae; Magnoliae Flos], Xanthium strumarium L. [Asteraceae; Xanthii Fructus], Chrysanthemum morifolium Ramat. [Asteraceae; Chrysanthemi Flos] 10 g, Angelica dahurica Benth. et Hooker f. [Apiaceae; Angelicae Dahuricae Radix] 9 g, Mentha arvensis var. piperascens Makinv. [Lamiaceae; Menthae Herba], Saposhnikovia divaricata Schiskin [Apiaceae; Saposhnikovia Radix] 6 g, Ligusticum chuanxiong Hort [Apiaceae; Ligustici Rhizoma] 5 g, Asarum sieboldii Miq. [Aristolochiaceae; Asari Herba Cum Radix] 3 g | - wind-cold: Schizonepeta tenuifolia (Benth.) Briq. [Labiatae; Schizonepetae Spica], Cinnamomum cassia Blume [Lauraceae; Cinnamomi Ramulus] - yellow sticky rhinorrhea or fishy smell and wind-fever: Scutellaria baicalensis Georgi [Labiatae; Scutellariae Radix], Forsythia suspensa (Thunb.) Vahl [Oleaceae; Forsythiae Fructus], Taraxacum platycarpum H. Dahlsi [Asteraceae; Taraxci Herba] - spleen-lung qi deficiency: Astragalus membranaceus Bunge [Leguminosae; Astragali Radix], Dioscorea batatas Decne. [Dioscoreaceae; Dioscoreae Rhizoma] | 2~5wks | None | N / N |
| Xia 2019 | Huodan tablet | Tablet | Agastache rugosa (Fisch. et Meyer) O. Kuntze [Lamiaceae; Agastachis Herba], Sus scrofa domesticus Brisson [Suidae; Suilus Fel] | None | 8wks | None | Y – produced by Shanghai Industrial United Group Great Wall Pharmaceutical Co., Ltd. / N |
| Xiang 2017 | Biyuan tongqiao granule | Granule | Magnolia denudata Desr. [Magnoliaceae; Magnoliae Flos], Xanthium strumarium L. [Asteraceae; Xanthii Fructus], Ephedra sinica Stapf. [Ephedraceae; Ephedrae Herba], Angelica dahurica Benth. et Hooker f. [Apiaceae; Angelicae Dahuricae Radix], Mentha arvensis var. piperascens Makinv. [Lamiaceae; Menthae Herba], Scutellaria baicalensis Georgi [Labiatae; Scutellariae Radix], Forsythia suspensa (Thunb.) Vahl [Oleaceae; Forsythiae Fructus], Chrysanthemum indicum L [Asteraceae; Chrysanthemi Indici Flos], Trichosanthes kirilowii Maxim. [Cucurbitaceae; Trichosanthis Radix], Rhemannia glutinosa (Gaertner) Liboschitz [Scrophulariaceae; Rehmanniae Radix], Salvia miltiorrhiza Bunge. [Labiatae; Salviae Miltiorrhizae Radix], Poria cocos (Schw.) Wolf [Polyporaceae; Poria(Hoelen)], Glycyrrhiza uralensis Fisch. [Leguminosae; Glycyrrhizae Radix] | None | 4wks | None | Y – produced by Shandong New Times Pharmaceutical Co., Ltd. / N |
| Xie 2021 | Qingqiao decoction | Decoction | Astragalus membranaceus Bunge [Leguminosae; Astragali Radix] 30 g, Atractylodes macrocepha-la Koidz [Asteraceae; Atractylodis Rhizoma Alba] 15 g, Codonopsis pilosulae (Fr.) Nannf. [Campunulaceae; Codonopsis Pilosulae Radix], Scutellaria baicalensis Georgi [Labiatae; Scutellariae Radix], Magnolia denudata Desr. [Magnoliaceae; Magnoliae Flos], Xanthium strumarium L. [Asteraceae; Xanthii Fructus], Saururus chinensis Baill. [Saururaceae; Houttuyniae Herba], Angelica gigas Nakai [Apiaceae; Angelicae Gigantis Radix], Acorus gramineus Sol. ex Aiton [Acoraceae; Acori Graminei Rhizoma] 10 g, Glycyrrhiza uralensis Fisch. [Leguminosae; Glycyrrhizae Radix], Akebia quinata Decne. [Lardizabalaceae; Akebiae Caulis] 5 g | None | 30d | 1yr | N / N |
| Xin 2010 | Bazhen decoction | Decoction | Angelica gigas Nakai [Apiaceae; Angelicae Gigantis Radix], Paeonia lactiflora Pall. [Paeoniaceae; Paeoniae Radix Rubra], Rehmannia glutinosa var. purpurea (Makino) Makino et Nemoto [Scrophulariaceae; Rehmanniae Radix], Poria cocos (Schw.) Wolf [Polyporaceae; Poria(Hoelen)] 12 g, Ligusticum chuanxiong Hort [Apiaceae; Ligustici Rhizoma], Codonopsis pilosulae (Fr.) Nannf. [Campunulaceae; Codonopsis Pilosulae Radix], Asarum sieboldii Miq. [Aristolochiaceae; Asari Herba Cum Radix] 9 g, Xanthium strumarium L. [Asteraceae; Xanthii Fructus] 6 g, Glycyrrhiza uralensis Fisch. [Leguminosae; Glycyrrhizae Radix] 3 g | - biliary-stagnation-heat type: Scutellaria baicalensis Georgi [Labiatae; Scutellariae Radix] 12 g, Bupleurum falcatum Linne [Apiaceae; Bupleuri Radix] 6 g, Gardenia jasminoides var. grandiflora (Lour.) Nakai [Rubiaceae; Gardeniae Fructus] 9 g - spleen and stomach damp-heat type: Agastache rugosa (Fisch. et Meyer) O. Kuntze [Lamiaceae; Agastachis Herba], Coptis deltoidea C.Y. Cheng et Hsiao [Ranunculaceae; Coptidis Rhizoma] 9 g, Coix lachryma-jobi var. ma-yeun (Roman.) Stapf [Gramineae; Coicis Semen] 6 g - lung-spleen deficiency-cold type: Astragalus membranaceus Bunge [Leguminosae; Astragali Radix] 15 g, Atractylodes macrocepha-la Koidz [Asteraceae; Atractylodis Rhizoma Alba], Citrus unshiu Markovich [Rutaceae; Citri Pericarpium] 6 g - qi and blood stasis type: Prunus persica (L.) Batsch [Rosaceae; Persicae Semen], Carthamus tinctorius L. [Asteraceae; Carthami Flos] 9 g | 4wks | 6mo | N / N |
| Xing 2015 | No name | Decoction | -damp-heat type: Coix lachryma-jobi var. ma-yeun (Roman.) Stapf [Gramineae; Coicis Semen], Saururus chinensis Baill. [Saururaceae; Houttuyniae Herba] 20 g, Poria cocos (Schw.) Wolf [Polyporaceae; Poria(Hoelen)], Ligusticum chuanxiong Hort [Apiaceae; Ligustici Rhizoma] 15 g, Scutellaria baicalensis Georgi [Labiatae; Scutellariae Radix], Agastache rugosa (Fisch. et Meyer) O. Kuntze [Lamiaceae; Agastachis Herba], Fritillaria thunbergii Miq. [Liliaceae; Fritillariae Thunbergii Bulbus], Angelica dahurica Benth. et Hooker f. [Apiaceae; Angelicae Dahuricae Radix], Saposhnikovia divaricata Schiskin [Apiaceae; Saposhnikovia Radix], Bupleurum falcatum Linne [Apiaceae; Bupleuri Radix], Magnolia denudata Desr. [Magnoliaceae; Magnoliae Flos], Gardenia jasminoides var. grandiflora (Lour.) Nakai [Rubiaceae; Gardeniae Fructus], Akebia quinata Decne. [Lardizabalaceae; Akebiae Caulis] 10 g, Glycyrrhiza uralensis Fisch. [Leguminosae; Glycyrrhizae Radix] 5 g  -deficiency-cold type: Poria cocos (Schw.) Wolf [Polyporaceae; Poria(Hoelen)] 20 g, Platycodon grandiflorum (Jacq.) A. DC. [Campanulaceae; Platycodi Radix], Atractylodes macrocepha-la Koidz [Asteraceae; Atractylodis Rhizoma Alba], Codonopsis pilosulae (Fr.) Nannf. [Campunulaceae; Codonopsis Pilosulae Radix], Ligusticum chuanxiong Hort [Apiaceae; Ligustici Rhizoma] 15 g, Xanthium strumarium L. [Asteraceae; Xanthii Fructus], Angelica dahurica Benth. et Hooker f. [Apiaceae; Angelicae Dahuricae Radix], Zingiber officinale Rosc. [Zingiberaceae; Zingiberis Rhizoma Siccus] 10 g, Bupleurum falcatum Linne [Apiaceae; Bupleuri Radix], Cimicifuga heracleifolia Kom. [Ranunculaceae; Cimicifugae Rhizoma], Asarum sieboldii Miq. [Aristolochiaceae; Asari Herba Cum Radix], Glycyrrhiza uralensis Fisch. [Leguminosae; Glycyrrhizae Radix] 5 g | Clinically, it can be added or subtracted according to the symptoms of the patient | 1~2wks | None | N / N |
| Xu 2013 | Xiangju capsule | Capsule | Platycarya strobilacea Sieb et Zucc. [Juglandaceae; Platycaryae Foluim], Prunella vulgaris var. lilacina Nakai [Lamiaceae; Prunellae Spica], Chrysanthemum indicum L [Asteraceae; Chrysanthemi Indici Flos], Astragalus membranaceus Bunge [Leguminosae; Astragali Radix], Magnolia denudata Desr. [Magnoliaceae; Magnoliae Flos], Saposhnikovia divaricata Schiskin [Apiaceae; Saposhnikovia Radix], Angelica dahurica Benth. et Hooker f. [Apiaceae; Angelicae Dahuricae Radix], Glycyrrhiza uralensis Fisch. [Leguminosae; Glycyrrhizae Radix], Ligusticum chuanxiong Hort [Apiaceae; Ligustici Rhizoma] | None | 3mo | 6mo~1yr (mean 6.4mo) | Y – produced by Shandong Buchang Pharmaceutical Co., Ltd. / N |
| Xu 2019 | Biyuan tongqiao decoction | Decoction | Rhemannia glutinosa (Gaertner) Liboschitz [Scrophulariaceae; Rehmanniae Radix] 20 g, Angelica gigas Nakai [Apiaceae; Angelicae Gigantis Radix], Ligusticum chuanxiong Hort [Apiaceae; Ligustici Rhizoma], Magnolia denudata Desr. [Magnoliaceae; Magnoliae Flos], Xanthium strumarium L. [Asteraceae; Xanthii Fructus] 15 g, Lonicera japonica Thunb. [Caprifoliaceae; Lonicerae Flos], Chrysanthemum morifolium Ramat. [Asteraceae; Chrysanthemi Flos], Schizonepeta tenuifolia (Benth.) Briq. [Labiatae; Schizonepetae Spica], Saposhnikovia divaricata Schiskin [Apiaceae; Saposhnikovia Radix], Angelica dahurica Benth. et Hooker f. [Apiaceae; Angelicae Dahuricae Radix] 10 g | None | 8wks | None | N / N |
| Yang 2013 | Biyankang decoction | Decoction | Astragalus membranaceus Bunge [Leguminosae; Astragali Radix], Rehmannia glutinosa var. purpurea (Makino) Makino et Nemoto [Scrophulariaceae; Rehmanniae Radix] 30 g, Codonopsis pilosulae (Fr.) Nannf. [Campunulaceae; Codonopsis Pilosulae Radix] 20 g, Atractylodes macrocepha-la Koidz [Asteraceae; Atractylodis Rhizoma Alba], Poria cocos (Schw.) Wolf [Polyporaceae; Poria(Hoelen)], Angelica gigas Nakai [Apiaceae; Angelicae Gigantis Radix], Paeonia lactiflora Pall. [Paeoniaceae; Paeoniae Radix Rubra], Ligusticum chuanxiong Hort [Apiaceae; Ligustici Rhizoma], Saururus chinensis Baill. [Saururaceae; Houttuyniae Herba], Scutellaria baicalensis Georgi [Labiatae; Scutellariae Radix] 15 g, Xanthium strumarium L. [Asteraceae; Xanthii Fructus], Magnolia denudata Desr. [Magnoliaceae; Magnoliae Flos] 12 g | None | 6~10wks | 3yr | N / N |
| Yang 2016 | No name | Decoction | Lonicera japonica Thunb. [Caprifoliaceae; Lonicerae Flos] 30 g, Astragalus membranaceus Bunge [Leguminosae; Astragali Radix], Saururus chinensis Baill. [Saururaceae; Houttuyniae Herba] 20 g, Platycodon grandiflorum (Jacq.) A. DC. [Campanulaceae; Platycodi Radix], Agastache rugosa (Fisch. et Meyer) O. Kuntze [Lamiaceae; Agastachis Herba], Scutellaria baicalensis Georgi [Labiatae; Scutellariae Radix], Mentha arvensis var. piperascens Makinv. [Lamiaceae; Menthae Herba], Xanthium strumarium L. [Asteraceae; Xanthii Fructus], Ligusticum chuanxiong Hort [Apiaceae; Ligustici Rhizoma], Magnolia denudata Desr. [Magnoliaceae; Magnoliae Flos], Atractylodes macrocepha-la Koidz [Asteraceae; Atractylodis Rhizoma Alba], Angelica tenuissima Nakai [Apiaceae; Ligustici Tenuissimae Radix] 10 g, Glycyrrhiza uralensis Fisch. [Leguminosae; Glycyrrhizae Radix] 6 g, Asarum sieboldii Miq. [Aristolochiaceae; Asari Herba Cum Radix] 3 g | None | 2wks | 1yr | N / N |
| Yang 2018 | Biyuan decoction | Decoction | Astragalus membranaceus Bunge [Leguminosae; Astragali Radix] 30 g, Magnolia denudata Desr. [Magnoliaceae; Magnoliae Flos], Xanthium strumarium L. [Asteraceae; Xanthii Fructus] 15 g, Angelica gigas Nakai [Apiaceae; Angelicae Gigantis Radix], Saururus chinensis Baill. [Saururaceae; Houttuyniae Herba], Atractylodes macrocepha-la Koidz [Asteraceae; Atractylodis Rhizoma Alba] 12 g, Acorus gramineus Sol. ex Aiton [Acoraceae; Acori Graminei Rhizoma], Scutellaria baicalensis Georgi [Labiatae; Scutellariae Radix], Codonopsis pilosulae (Fr.) Nannf. [Campunulaceae; Codonopsis Pilosulae Radix] 10 g, Akebia quinata Decne. [Lardizabalaceae; Akebiae Caulis] 8 g, Glycyrrhiza uralensis Fisch. [Leguminosae; Glycyrrhizae Radix] 6 g | - severe nasal congestion: Mentha arvensis var. piperascens Makinv. [Lamiaceae; Menthae Herba] 9 g - temporal pain: Angelica dahurica Benth. et Hooker f. [Apiaceae; Angelicae Dahuricae Radix] 12 g, Ligusticum chuanxiong Hort [Apiaceae; Ligustici Rhizoma] 9 g - parietal headache: Angelica tenuissima Nakai [Apiaceae; Ligustici Tenuissimae Radix] 9 g - much runny nose: Patrinia villosa (Thunb.) Juss [Valerianaceae; Patriniae Radix] 12 g | 4wks | None | N / N |
| Yang 2020 | Cangerzi biyan capsule | Capsule | Xanthium strumarium L. [Asteraceae; Xanthii Fructus], Gypsum [Gypsum Fibrosum], Angelica dahurica Benth. et Hooker f. [Apiaceae; Angelicae Dahuricae Radix], Dryobalanops aromatica Gaertn. f. [Dipterocarpaceae; Borneolum], Magnolia denudata Desr. [Magnoliaceae; Magnoliae Flos], Mentha arvensis var. piperascens Makinv. [Lamiaceae; Menthae Herba], Scutellaria baicalensis Georgi [Labiatae; Scutellariae Radix] | None | 2wks | None | N / N |
| Yao 2019 | Shenlingbaizhu powder and Cangerzi powder | Decoction | Glycyrrhiza uralensis Fisch. [Leguminosae; Glycyrrhizae Radix], Coix lachryma-jobi var. ma-yeun (Roman.) Stapf [Gramineae; Coicis Semen], Codonopsis pilosulae (Fr.) Nannf. [Campunulaceae; Codonopsis Pilosulae Radix] 15 g, Xanthium strumarium L. [Asteraceae; Xanthii Fructus], Magnolia denudata Desr. [Magnoliaceae; Magnoliae Flos], Angelica dahurica Benth. et Hooker f. [Apiaceae; Angelicae Dahuricae Radix], Ligusticum chuanxiong Hort [Apiaceae; Ligustici Rhizoma], Scutellaria baicalensis Georgi [Labiatae; Scutellariae Radix], Mentha arvensis var. piperascens Makinv. [Lamiaceae; Menthae Herba], Fritillaria thunbergii Miq. [Liliaceae; Fritillariae Thunbergii Bulbus], Glycine max Merr. [Leguminosae; Sojae Semen Praeparatum], Chrysanthemum morifolium Ramat. [Asteraceae; Chrysanthemi Flos], Dolichos lablab L. [Leguminosae; Dolichoris Semen], Platycodon grandiflorum (Jacq.) A. DC. [Campanulaceae; Platycodi Radix], Dioscorea batatas Decne. [Dioscoreaceae; Dioscoreae Rhizoma], Poria cocos (Schw.) Wolf [Polyporaceae; Poria(Hoelen)], Atractylodes macrocepha-la Koidz [Asteraceae; Atractylodis Rhizoma Alba] 10 g, Amomum villosum Lour. [Zingiberaceae; Amomi Fuctus], Nelumbo nucifera Gaertner [Nymphaceae; Nelumbinis Semen] 6 g | None | 4wks | None | N / N |
| Yun 2019 | Biyuan tongqiao granule | Granule | Magnolia denudata Desr. [Magnoliaceae; Magnoliae Flos], Xanthium strumarium L. [Asteraceae; Xanthii Fructus], Ephedra sinica Stapf. [Ephedraceae; Ephedrae Herba], Angelica dahurica Benth. et Hooker f. [Apiaceae; Angelicae Dahuricae Radix], Mentha arvensis var. piperascens Makinv. [Lamiaceae; Menthae Herba], Angelica tenuissima Nakai [Apiaceae; Ligustici Tenuissimae Radix], Scutellaria baicalensis Georgi [Labiatae; Scutellariae Radix], Forsythia suspensa (Thunb.) Vahl [Oleaceae; Forsythiae Fructus], Chrysanthemum indicum L [Asteraceae; Chrysanthemi Indici Flos], Trichosanthes kirilowii Maxim. [Cucurbitaceae; Trichosanthis Radix], Rhemannia glutinosa (Gaertner) Liboschitz [Scrophulariaceae; Rehmanniae Radix], Salvia miltiorrhiza Bunge. [Labiatae; Salviae Miltiorrhizae Radix], Poria cocos (Schw.) Wolf [Polyporaceae; Poria(Hoelen)], Glycyrrhiza uralensis Fisch. [Leguminosae; Glycyrrhizae Radix] | None | 4wks | None | Y – produced by Shandong New Times Pharmaceutical Co., Ltd. / N |
| Zhang 2004 | Ephedra and pueraria decoction | Decoction | Pueraria thunbergiana Benth. [Leguminosae; Puerariae Radix], Magnolia denudata Desr. [Magnoliaceae; Magnoliae Flos], Xanthium strumarium L. [Asteraceae; Xanthii Fructus], Agastache rugosa (Fisch. et Meyer) O. Kuntze [Lamiaceae; Agastachis Herba], Paeonia lactiflora Pall. [Paeoniaceae; Paeoniae Radix Rubra], Zingiber officinale Rosc. [Zingiberaceae; Zingiberis Rhizoma Recens], Platycodon grandiflorum (Jacq.) A. DC. [Campanulaceae; Platycodi Radix] 9 g, Ephedra sinica Stapf. [Ephedraceae; Ephedrae Herba] 6 g, Zizyphus jujuba var. inermis Rehder [Rhamnaceae; Zizyphi Fructus] 4 pieces, Glycyrrhiza uralensis Fisch. [Leguminosae; Glycyrrhizae Radix] 3 g | - cold stagnation, blood stasis, head and brain cold pain: Asarum sieboldii Miq. [Aristolochiaceae; Asari Herba Cum Radix] 3 g, Gypsum [Gypsum Fibrosum] 10 g | 10~30d (mean 22d) | 6mo | N / N |
| Zhang 2015 | Tongbi decoction | Decoction | Poria cocos (Schw.) Wolf [Polyporaceae; Poria(Hoelen)] 12 g, Atractylodes macrocepha-la Koidz [Asteraceae; Atractylodis Rhizoma Alba], Angelica dahurica Benth. et Hooker f. [Apiaceae; Angelicae Dahuricae Radix], Citrus unshiu Markovich [Rutaceae; Citri Pericarpium], Xanthium strumarium L. [Asteraceae; Xanthii Fructus], Codonopsis pilosulae (Fr.) Nannf. [Campunulaceae; Codonopsis Pilosulae Radix], Magnolia denudata Desr. [Magnoliaceae; Magnoliae Flos], Dioscorea batatas Decne. [Dioscoreaceae; Dioscoreae Rhizoma] 10 g, Centipeda minima (L.) A. Br. Et Aschers. [Asteraceae; Centipedae Herba] 9 g, Mentha arvensis var. piperascens Makinv. [Lamiaceae; Menthae Herba] 6 g, Ephedra sinica Stapf. [Ephedraceae; Ephedrae Herba] 3 g | None | 10d | 20d | N / N |
| Zhang 2016a | No name | Decoction | Lonicera japonica Thunb. [Caprifoliaceae; Lonicerae Flos], Saururus chinensis Baill. [Saururaceae; Houttuyniae Herba] 20 g, Magnolia denudata Desr. [Magnoliaceae; Magnoliae Flos], Platycodon grandiflorum (Jacq.) A. DC. [Campanulaceae; Platycodi Radix], Agastache rugosa (Fisch. et Meyer) O. Kuntze [Lamiaceae; Agastachis Herba], Xanthium strumarium L. [Asteraceae; Xanthii Fructus], Chrysanthemum morifolium Ramat. [Asteraceae; Chrysanthemi Flos] 10 g, Angelica dahurica Benth. et Hooker f. [Apiaceae; Angelicae Dahuricae Radix] 9 g, Mentha arvensis var. piperascens Makinv. [Lamiaceae; Menthae Herba], Saposhnikovia divaricata Schiskin [Apiaceae; Saposhnikovia Radix] 6 g, Ligusticum chuanxiong Hort [Apiaceae; Ligustici Rhizoma] 5 g, Asarum sieboldii Miq. [Aristolochiaceae; Asari Herba Cum Radix] 3 g | - severe wind cold: Schizonepeta tenuifolia (Benth.) Briq. [Labiatae; Schizonepetae Spica], Cinnamomum cassia Blume [Lauraceae; Cinnamomi Ramulus] - severe wind fever: Taraxacum platycarpum H. Dahlsi [Asteraceae; Taraxci Herba], Scutellaria baicalensis Georgi [Labiatae; Scutellariae Radix], Forsythia suspensa (Thunb.) Vahl [Oleaceae; Forsythiae Fructus] - spleen lung qi deficiency: Dioscorea batatas Decne. [Dioscoreaceae; Dioscoreae Rhizoma], Astragalus membranaceus Bunge [Leguminosae; Astragali Radix] | 2~5wks | None | N / N |
| Zhang 2016b | Biyuan tongqiao granule | Granule | Magnolia denudata Desr. [Magnoliaceae; Magnoliae Flos], Angelica dahurica Benth. et Hooker f. [Apiaceae; Angelicae Dahuricae Radix], Xanthium strumarium L. [Asteraceae; Xanthii Fructus], Ephedra sinica Stapf. [Ephedraceae; Ephedrae Herba], Mentha arvensis var. piperascens Makinv. [Lamiaceae; Menthae Herba], Angelica tenuissima Nakai [Apiaceae; Ligustici Tenuissimae Radix], Scutellaria baicalensis Georgi [Labiatae; Scutellariae Radix], Forsythia suspensa (Thunb.) Vahl [Oleaceae; Forsythiae Fructus], Chrysanthemum indicum L [Asteraceae; Chrysanthemi Indici Flos], Trichosanthes kirilowii Maxim. [Cucurbitaceae; Trichosanthis Radix], Rhemannia glutinosa (Gaertner) Liboschitz [Scrophulariaceae; Rehmanniae Radix], Salvia miltiorrhiza Bunge. [Labiatae; Salviae Miltiorrhizae Radix], Poria cocos (Schw.) Wolf [Polyporaceae; Poria(Hoelen)], Glycyrrhiza uralensis Fisch. [Leguminosae; Glycyrrhizae Radix] | None | 4wks | 6mo | Y – produced by Shandong New Times Pharmaceutical Co., Ltd. / N |
| Zhang 2018 | Tuoli tongdou decoction | Granule | Astragalus membranaceus Bunge [Leguminosae; Astragali Radix], Poria cocos (Schw.) Wolf [Polyporaceae; Poria(Hoelen)], Gleditsia japonica var. korainensis (Nak.) Nakai [Leguminosae; Gleditsiae Semen] 15 g, Codonopsis pilosulae (Fr.) Nannf. [Campunulaceae; Codonopsis Pilosulae Radix], Paeonia albiflora Pallas var. trichocarpa Bunge [Paeoniaceae; Paeoniae Radix Alba], Citrus unshiu Markovich [Rutaceae; Citri Pericarpium], Morus alba L. [Moraceae; Mori Radicis Cortex], Alisma orientalis (Sam) Juzep [Alismataceae; Alismatis Rhizoma], Lonicera japonica Thunb. [Caprifoliaceae; Lonicerae Flos], Agastache rugosa (Fisch. et Meyer) O. Kuntze [Lamiaceae; Agastachis Herba], Angelica dahurica Benth. et Hooker f. [Apiaceae; Angelicae Dahuricae Radix] 12 g, Atractylodes macrocepha-la Koidz [Asteraceae; Atractylodis Rhizoma Alba], Magnolia denudata Desr. [Magnoliaceae; Magnoliae Flos] 10 g, Angelica gigas Nakai [Apiaceae; Angelicae Gigantis Radix] 9 g, Ligusticum chuanxiong Hort [Apiaceae; Ligustici Rhizoma], Glycyrrhiza uralensis Fisch. [Leguminosae; Glycyrrhizae Radix] 6 g | None | 4wks | None | Y – produced by Guangzhou Yifang Pharmaceutical Co., Ltd. / N |
| Zhang 2019a | Biyuan decoction | Decoction | Phragmites communis Trin. [Gramineae; Phragmitis Rhizoma] 15 g, Morus alba L. [Moraceae; Mori Folium], Magnolia denudata Desr. [Magnoliaceae; Magnoliae Flos], Saururus chinensis Baill. [Saururaceae; Houttuyniae Herba], Ligusticum chuanxiong Hort [Apiaceae; Ligustici Rhizoma], Scutellaria baicalensis Georgi [Labiatae; Scutellariae Radix] 10 g, Angelica dahurica Benth. et Hooker f. [Apiaceae; Angelicae Dahuricae Radix], Xanthium strumarium L. [Asteraceae; Xanthii Fructus], Platycodon grandiflorum (Jacq.) A. DC. [Campanulaceae; Platycodi Radix], Acorus calamus var. angustatus Bess. [Acoraceae; Acori Calami Rhizoma] 6 g, Glycyrrhiza uralensis Fisch. [Leguminosae; Glycyrrhizae Radix] 3 g | - parietal headache: Angelica tenuissima Nakai [Apiaceae; Ligustici Tenuissimae Radix] - pain in the forehead and supra-orbital bone: Vitex rotundifolia L. Fil. [Verbenaceae; Viticis Fructus] - occipital area and neck pain: Pueraria thunbergiana Benth. [Leguminosae; Puerariae Radix] - bilateral temple pain: Bupleurum falcatum Linne [Apiaceae; Bupleuri Radix] - much phlegm: Prunus armeniaca L. var. ansu Maxim. [Rosaceae; Armeniacae Semen], Trichosanthes kirilowii Maxim. [Cucurbitaceae; Trichosanthis Fuctus] | 4mo | None | N / N |
| Zhang 2019b | Xinqian ganjie decoction | Decoction | Coix lachryma-jobi var. ma-yeun (Roman.) Stapf [Gramineae; Coicis Semen] 12 g, Peucedanum decursivum (Miq.) Maxim. [Apiaceae; Peucedani Radix], Trichosanthes kirilowii Maxim. [Cucurbitaceae; Trichosanthis Radix] 9 g, Magnolia denudata Desr. [Magnoliaceae; Magnoliae Flos], Saposhnikovia divaricata Schiskin [Apiaceae; Saposhnikovia Radix], Platycodon grandiflorum (Jacq.) A. DC. [Campanulaceae; Platycodi Radix] 6 g, Glycyrrhiza uralensis Fisch. [Leguminosae; Glycyrrhizae Radix] 3 g | - nasal congestion: Asarum sieboldii Miq. [Aristolochiaceae; Asari Herba Cum Radix] 3 g, Agastache rugosa (Fisch. et Meyer) O. Kuntze [Lamiaceae; Agastachis Herba] 6 g - clear nasal discharge: Prunus armeniaca L. var. ansu Maxim. [Rosaceae; Armeniacae Semen], Fritillaria thunbergii Miq. [Liliaceae; Fritillariae Thunbergii Bulbus] 9 g - yellow thick nasal discharge: Fructus trichosanthis [Trichosanthes Hisrilowii] 9 g - mucosal edema: Poria cocos (Schw.) Wolf [Polyporaceae; Poria(Hoelen)], Alisma orientalis (Sam) Juzep [Alismataceae; Alismatis Rhizoma] 10 g - mucosal red and swelling: Paeonia lactiflora Pall. [Paeoniaceae; Paeoniae Radix Rubra], Paeonia suffruticosa Andrews [Ranunculaceae; Moutan Cortex] 10 g | 12wks | 9mo | N / N |
| Zhang 2020 | Shenlingbaizhu powder | Decoction | Coix lachryma-jobi var. ma-yeun (Roman.) Stapf [Gramineae; Coicis Semen] 24 g, Codonopsis pilosulae (Fr.) Nannf. [Campunulaceae; Codonopsis Pilosulae Radix], Atractylodes macrocepha-la Koidz [Asteraceae; Atractylodis Rhizoma Alba], Poria cocos (Schw.) Wolf [Polyporaceae; Poria(Hoelen)], Dioscorea batatas Decne. [Dioscoreaceae; Dioscoreae Rhizoma] 15 g, Dolichos lablab L. [Leguminosae; Dolichoris Semen] 12 g, Platycodon grandiflorum (Jacq.) A. DC. [Campanulaceae; Platycodi Radix], Citrus unshiu Markovich [Rutaceae; Citri Pericarpium], Ligusticum chuanxiong Hort [Apiaceae; Ligustici Rhizoma] 9 g, Amomum villosum Lour. [Zingiberaceae; Amomi Fuctus], Magnolia denudata Desr. [Magnoliaceae; Magnoliae Flos] 6 g, Glycyrrhiza uralensis Fisch. [Leguminosae; Glycyrrhizae Radix] 3 g | None | 4wks | None | N / N |
| Zhang 2021 | Qingre lishi quyu decoction | Decoction | Saururus chinensis Baill. [Saururaceae; Houttuyniae Herba] 18 g, Angelica gigas Nakai [Apiaceae; Angelicae Gigantis Radix], Alisma orientalis (Sam) Juzep [Alismataceae; Alismatis Rhizoma] 15 g, Xanthium strumarium L. [Asteraceae; Xanthii Fructus], Angelica dahurica Benth. et Hooker f. [Apiaceae; Angelicae Dahuricae Radix] 12 g, Magnolia denudata Desr. [Magnoliaceae; Magnoliae Flos], Ligusticum chuanxiong Hort [Apiaceae; Ligustici Rhizoma], Scutellaria baicalensis Georgi [Labiatae; Scutellariae Radix], Chrysanthemum morifolium Ramat. [Asteraceae; Chrysanthemi Flos], Agastache rugosa (Fisch. et Meyer) O. Kuntze [Lamiaceae; Agastachis Herba], Gentiana scabra Bunge [Gentianaceae; Gentianae Radix], Gardenia jasminoides var. grandiflora (Lour.) Nakai [Rubiaceae; Gardeniae Fructus] 10 g, Glycyrrhiza uralensis Fisch. [Leguminosae; Glycyrrhizae Radix] 6 g | None | 3mo | None | N / N |
| Zhou 2008 | Bídòu líng tāng | Decoction | Astragalus membranaceus Bunge [Leguminosae; Astragali Radix] 20 g, Saururus chinensis Baill. [Saururaceae; Houttuyniae Herba], Atractylodes macrocepha-la Koidz [Asteraceae; Atractylodis Rhizoma Alba], Codonopsis pilosulae (Fr.) Nannf. [Campunulaceae; Codonopsis Pilosulae Radix] 15 g, Angelica dahurica Benth. et Hooker f. [Apiaceae; Angelicae Dahuricae Radix] 12 g, Xanthium strumarium L. [Asteraceae; Xanthii Fructus], Vitex rotundifolia L. Fil. [Verbenaceae; Viticis Fructus], Saposhnikovia divaricata Schiskin [Apiaceae; Saposhnikovia Radix], Scutellaria baicalensis Georgi [Labiatae; Scutellariae Radix], Platycodon grandiflorum (Jacq.) A. DC. [Campanulaceae; Platycodi Radix] 10 g, Glycyrrhiza uralensis Fisch. [Leguminosae; Glycyrrhizae Radix], Buthus martensi Karsch [Buthidae; Scorpio] 6 g | None | 2wks | 6mo | N / N |
| Zhou 2012 | Biyuanshu oral liquid | Oral liquid | Xanthium strumarium L. [Asteraceae; Xanthii Fructus], Magnolia denudata Desr. [Magnoliaceae; Magnoliae Flos], Mentha arvensis var. piperascens Makinv. [Lamiaceae; Menthae Herba], Angelica dahurica Benth. et Hooker f. [Apiaceae; Angelicae Dahuricae Radix], Scutellaria baicalensis Georgi [Labiatae; Scutellariae Radix], Gardenia jasminoides var. grandiflora (Lour.) Nakai [Rubiaceae; Gardeniae Fructus], Bupleurum falcatum Linne [Apiaceae; Bupleuri Radix], Asarum sieboldii Miq. [Aristolochiaceae; Asari Herba Cum Radix], Ligusticum chuanxiong Hort [Apiaceae; Ligustici Rhizoma], Astragalus membranaceus Bunge [Leguminosae; Astragali Radix], Akebia quinata Decne. [Lardizabalaceae; Akebiae Caulis], Platycodon grandiflorum (Jacq.) A. DC. [Campanulaceae; Platycodi Radix], Poria cocos (Schw.) Wolf [Polyporaceae; Poria(Hoelen)] | None | 15d | None | N / N |
| Zhou 2013 | Longdan Xiegan decoction | Decoction | Rehmannia glutinosa var. purpurea (Makino) Makino et Nemoto [Scrophulariaceae; Rehmanniae Radix] 20 g, Alisma orientalis (Sam) Juzep [Alismataceae; Alismatis Rhizoma] 12 g, Bupleurum falcatum Linne [Apiaceae; Bupleuri Radix] 10 g, Scutellaria baicalensis Georgi [Labiatae; Scutellariae Radix], Gardenia jasminoides var. grandiflora (Lour.) Nakai [Rubiaceae; Gardeniae Fructus], Akebia quinata Decne. [Lardizabalaceae; Akebiae Caulis], Plantago asiatica L. [Plantaginaceae; Plantaginis Semen] 9 g, Angelica gigas Nakai [Apiaceae; Angelicae Gigantis Radix] 8 g, Gentiana scabra Bunge [Gentianaceae; Gentianae Radix], Glycyrrhiza uralensis Fisch. [Leguminosae; Glycyrrhizae Radix] 6 g | None | 2wks | None | N / N |
| Zhu 2014 | Qingbi decoction | Decoction | Lonicera japonica Thunb. [Caprifoliaceae; Lonicerae Flos], Imperata cylindrica var. koenigii (Retz.) Perkins [Gramineae; Imperatae Rhizoma], Acorus gramineus Sol. ex Aiton [Acoraceae; Acori Graminei Rhizoma], Saururus chinensis Baill. [Saururaceae; Houttuyniae Herba] 15 g, Gentiana scabra Bunge [Gentianaceae; Gentianae Radix], Xanthium strumarium L. [Asteraceae; Xanthii Fructus] 12 g, Scutellaria baicalensis Georgi [Labiatae; Scutellariae Radix], Angelica dahurica Benth. et Hooker f. [Apiaceae; Angelicae Dahuricae Radix], Glycyrrhiza uralensis Fisch. [Leguminosae; Glycyrrhizae Radix], Bupleurum falcatum Linne [Apiaceae; Bupleuri Radix], Magnolia denudata Desr. [Magnoliaceae; Magnoliae Flos], Curcuma aromatica Salisb. [Zingiberaceae; Curcumae Radix] 10 g, Ephedra sinica Stapf. [Ephedraceae; Ephedrae Herba], Cinnamomum cassia Blume [Lauraceae; Cinnamomi Ramulus] 6 g, Asarum sieboldii Miq. [Aristolochiaceae; Asari Herba Cum Radix] 3 g | None | 8wks | 1mo | N / N |
| Zou 2007 | Xiangju capsule | Capsule | Platycarya strobilacea Sieb et Zucc. [Juglandaceae; Platycaryae Foluim], Astragalus membranaceus Bunge [Leguminosae; Astragali Radix], Prunella vulgaris var. lilacina Nakai [Lamiaceae; Prunellae Spica], Chrysanthemum indicum L [Asteraceae; Chrysanthemi Indici Flos], Saposhnikovia divaricata Schiskin [Apiaceae; Saposhnikovia Radix], Magnolia denudata Desr. [Magnoliaceae; Magnoliae Flos], Angelica dahurica Benth. et Hooker f. [Apiaceae; Angelicae Dahuricae Radix], Glycyrrhiza uralensis Fisch. [Leguminosae; Glycyrrhizae Radix], Ligusticum chuanxiong Hort [Apiaceae; Ligustici Rhizoma] | None | 10d | 6mo | Y – produced by Shandong Buchang Pharmaceutical Co., Ltd. / N |
